# Global Mapping of H3K4 Trimethylation (H3K4me3) and Transcriptome Analysis Reveal Genes Involved in the Response to Epidemic Diarrhea Virus Infections in Pigs

**DOI:** 10.3390/ani9080523

**Published:** 2019-08-02

**Authors:** Haifei Wang, Li Yang, Huan Qu, Haiyue Feng, Shenglong Wu, Wenbin Bao

**Affiliations:** 1 Key Laboratory for Animal Genetics, Breeding, Reproduction and Molecular Design, College of Animal Science and Technology, Yangzhou University, Yangzhou 225009, China; 2 Joint International Research Laboratory of Agriculture & Agri-Product Safety, Yangzhou University, Yangzhou 225009, China

**Keywords:** pig, PEDV, gene expression, H3K4me3, transcriptional regulation

## Abstract

**Simple Summary:**

Porcine epidemic diarrhea virus seriously threatens the health of suckling pigs. In this study, global mapping of H3K4me3 and transcriptomic analyses in the jejunum of porcine epidemic diarrhea virus (PEDV)-infected and healthy piglets were performed by using chromatin immunoprecipitation sequencing and RNA-seq techniques. A subset of genes and H3K4 trimethylation (H3K4me3) histone modifications that are associated with PEDV infections were identified. The results revealed previously unknown and intriguing elements involved in the regulation of genes responsive to PEDV infections, which may aid in identifying key regulators and genetic resistant markers for PEDV infections.

**Abstract:**

Porcine epidemic diarrhea virus (PEDV) is currently detected as the main pathogen causing severe diarrhea in pig farms. The phenotypic alterations induced by pathogenic infections are usually tightly linked with marked changes in epigenetic modification and gene expression. We performed global mapping of H3K4 trimethylation (H3K4me3) and transcriptomic analyses in the jejunum of PEDV-infected and healthy piglets using chromatin immunoprecipitation sequencing and RNA-seq techniques. A total of 1885 H3K4me3 peaks that are associated with 1723 genes were characterized. Moreover, 290 differentially expressed genes were identified, including 104 up-regulated and 186 down-regulated genes. Several antiviral genes including 2’-5’-oligoadenylate synthetase 1 (*OAS1*), 2’-5’-oligoadenylate synthetase 2 (*OAS2*), ephrin B2 (*EFNB2*), and CDC28 protein kinase regulatory subunit 1B (*CKS1B*) with higher H3K4me3 enrichment and expression levels in PEDV-infected samples suggested the potential roles of H3K4me3 deposition in promoting their expressions. Transcription factor annotation analysis highlighted the potential roles of two transcription factors interferon regulatory factor 8 (IRF8) and Kruppel like factor 4 (KLF4) in modulating the differential expression of genes involved in PEDV infection. The results provided novel insights into PEDV infection from the transcriptomic and epigenetic layers and revealed previously unknown and intriguing elements potentially involved in the host responses.

## 1. Introduction

Porcine epidemic diarrhea, which is caused by the porcine epidemic diarrhea virus (PEDV), results in large economic losses in the pig industry because of the high morbidity and mortality, approaching 100% in neonatal piglets. Since first recognized in feeding and fattening pigs in England in 1971, PEDV had spread throughout much of Europe, Asia, and America by 2013 [1,2]. PEDV is an enveloped, positive-stranded RNA virus that belongs to the group 1 coronavirus [3]. PEDV replicates in the cytoplasm of villus epithelial cells and causes villi atrophy, shortening, and fusion, which leads to watery diarrhea, vomiting, and dehydration of infected animals. Genetic and phylogenetic analyses unveiled the presence of genetic diversity among PEDV prevalent in different countries, even in different regions of the same country [1,4], which increases the difficulties in prevention and control of PEDV infections. In recent years, PEDV is still identified as the main pathogen causing severe diarrhea in pig farms [5,6], highlighting the urgency to genetically improve the ability of pigs to resist PEDV infections. 

Infection of viruses triggers alterations in the host gene expression program mainly at the transcriptional level and may augment host responses to viral infections. Transcriptional profiles as the bridge between genomic portrait and protein expression profile play important roles in revealing the immune regulation mechanisms between host cells and viral infections. Recent studies have demonstrated the proteomic profile changes in the jejunum of PEDV-infected piglets and porcine small intestinal epithelial cell line (IPEC-J2) stimulated with PEDV and identified a subset of proteins and signaling pathways potentially responsible for its pathogenesis [7,8,9]. However, the transcriptional profile changes in the jejunum of PEDV-infected piglets remain poorly understood. Furthermore, whether PEDV infection induces alterations in the patterns of epigenetic mark H3K4 trimethylation (H3K4me3) that positively associates with gene activity is still scant now. It is required to provide biologically relevant insights at the transcriptional and epigenetic levels for fully understanding the virus–host interactions.

To investigate the changes in the patterns of H3K4me3 and transcriptome of the PEDV-infected jejunum and to identify the key regulators involved in PEDV pathogenesis, we characterized the modifications of H3K4me3 and gene expression changes in the jejunum from PEDV-infected piglets using chromatin immunoprecipitation sequencing (ChIP-seq) and RNA-seq techniques. We found alterations in the patterns of H3K4me3 and gene expression, and identified a collection of genes potentially involved in regulating PEDV infections. Our findings provided new insights into the pattern of H3K4me3 and transcriptional programs of PEDV infection, and revealed previously unknown and intriguing elements potentially involved in the host responses.

## 2. Materials and Methods

### 2.1. Ethics Statement

The animal study proposal was approved by the Institutional Animal Care and Use Committee (IACUC) of the Yangzhou University Animal Experiments Ethics Committee (permit number: SYXK (Su) IACUC 2012-0029). All experimental methods were conducted in accordance with the related guidelines and regulations.

### 2.2. Animals and Tissue Collection

Four Large White piglets (6 to 8 days old) naturally infected with PEDV and exhibiting clinical signs of porcine epidemic diarrhea including watery diarrhea and acute vomiting were selected as the experimental samples. In addition, four clinically healthy piglets were used as controls. All the animals were raised under the same conditions and humanely sacrificed for tissue collection. The proximal part of jejunum tissues was collected and rinsed with phosphate buffer saline (PBS), and stored in liquid nitrogen. The jejunum tissues were also fixed in 2.5% glutaraldehyde and 4% paraformaldehyde respectively for histopathological analysis. The intestinal contents and feces samples were collected for quantitative PEDV examination.

### 2.3. PEDV Examination by Quantitative Real-Time PCR (qRT-PCR)

The intestinal contents and feces were diluted by 500 μL PBS, freeze-thawed three times, and then centrifuged for the collection of the supernatant containing viruses. The supernatant was used to extract total RNA using the Trizol reagent (ThermoFisher, Waltham, MA, USA), and cDNA was synthesized with the PrimerScript RT reagent Kit with gDNA Eraser (Takara Biotechnology (Dalian) Co., Ltd., Dalian, China) following the manufacturer’s guidelines. The M gene of PEDV was amplified by qRT-PCR using the primers: F: 5’-GGACACATTCTTGGTGGTCT-3’, R: 5’-GTTTAGACTAAATGAAGCACTTTC-3’ [10]. The reaction conditions were as follows: 95 °C for 5 min, 35 cycles of 95 °C for 30 s, 55 °C for 30 s, 72 °C for 30 s, and 72 °C for 10 min. 

### 2.4. Histopathological Analysis

The glutaraldehyde-fixed jejunum segments were sheared into 1 cm^3^ fragments, washed several times with PBS, fixed in 1% osmic acid for 2 h, and dehydrated by washing with gradient ethanol. After infiltration and embedding procedures, samples were examined by transmission electron microscopic (TEM). Furthermore, the tissues fixed in 4% paraformaldehyde were routinely made into paraffin. A slice (about 5 μm thickness) of the tissue was cut for hematoxylin and eosin (H&E) staining. The histopathological differences of the jejunum tissues were analyzed under an optical microscope.

### 2.5. RNA-Seq Library Preparation and Sequencing

Total RNA of each jejunum sample was isolated using the Trizol reagent (ThermoFisher, Waltham, MA, USA) following the manufacturer’s instructions. RNA degradation and contamination were monitored on 1% agarose gels. The RNA concentration and integrity were checked using the Qubit RNA Assay Kit in Qubit 2.0 Fluorometer (Life Technologies, Carlsbad, CA, USA) and the RNA Nano 6000 Assay Kit of the Bioanalyzer 2100 system (Agilent Technologies, Palo Alto, CA, USA), respectively.

A total of 3 μg of RNA of each sample was used for library preparation. The mRNA was purified from total RNA with the poly-T oligo-attached magnetic beads, and then randomly cleaved into small fragments using RNA fragmentation buffer (NEB, Beijing, China). The first-strand cDNA was synthesized using random hexamer primer (NEB, Beijing, China) and M-MulV Reverse Transcriptase (NEB, Beijing, China), and the second strand was synthesized using DNA Polymerase I (NEB, Beijing, China) and RNase H (NEB, Beijing, China). The library fragments were purified, adenylated at 3’ ends, and ligated with sequencing adaptor. The cDNA fragments (250–300 bp) were then selected. PCR was performed with Phusion High-Fidelity DNA polymerase (NEB, Beijing, China), and the products were purified using AMPure XP system (Beckman Coulter, Beverly, USA). After cluster generation of the index-coded samples, the library preparations were sequenced on an Illumina PE150 Hiseq platform (Illumina, San Diego, CA, USA). 

### 2.6. RNA-Seq Data Analysis

Raw reads were first processed through in-house Perl scripts to remove reads containing adapter or ploy-N or with a base quality score lower than 20. The clean reads were aligned to the pig reference genome (Sscrofa11.1) using TopHat2 [11]. The HTSeq program was utilized to count the read numbers mapped to each gene [12]. The fragments per kilobase of transcript sequence per million base pairs (FPKM) of each gene were determined by the length of the gene and read count mapped to this gene. Differential expression genes between the infected and control groups were identified with the DESeq of R package [13]. The Benjamini and Hochberg’s method was applied to adjust the resulting *p* values for controlling the false discovery rate. Genes with an adjusted *p* < 0.05 were defined as differentially expressed. The cluster analysis was conducted for the differential expression genes using the K-means clustering analysis method.

### 2.7. Functional Annotation of Differentially Expressed Genes

Gene ontology (GO) enrichment analysis of differential expression genes was performed with GOseq [14]. We utilized the KOBAS software [15] to examine the statistical enrichment of differential expression genes in the KEGG database. The GO terms and pathways with *p* < 0.001 were considered to be statistically significant.

### 2.8. Validation of RNA-Seq Data by qRT-PCR

Total RNA of the samples was purified and reversely transcribed into cDNA using the PrimerScript RT reagent Kit with gDNA Eraser following the manufacturer’s protocols (Takara Biotechnology (Dalian) Co., Ltd., Dalian, China). Gene expression was quantified using qRT-PCR, with a volume of 20 μL containing 10 μL SYBR Green Mixture, 1 μL of each primer, 0.4 μL 50× ROX Reference Dye II, 1 μL cDNA, and 6.6 μL deionized water. The thermal conditions were as follows: 95 °C for 15 s, 40 cycles of 95 °C for 5 s, 60 °C for 30 s. The *GAPDH* gene was used as an internal control. Primer sequences are listed in Table S1. Each qRT-PCR assay was carried out in triplicate and the relative gene expression was calculated using the 2^−ΔΔCt^ method [16].

### 2.9. Transcription Factor Annotation and Motif Occurrences Analysis

The AnimalTFDB 2.0 (http://bioinfo.life.hust.edu.cn/AnimalTFDB/) is a feasible and useful tool to explore the expression of transcription factors from RNA-seq data [17]. Based on the Entrez Gene ID, identification of transcription factors was conducted by matching differential expression genes with the porcine transcription factors deposited in the AnimalTFDB 2.0 database according to the pipelines provided by the authors. The occurrences of transcription factor binding site motifs in the promoters of differential expression genes were scanned with the FIMO software [18], which computes a log-likelihood ratio score for each motif corresponding to each sequence position and converts this score to a *p*-value. The *p*-value refers to the probability of a random sequence of the same length as the motif matching that position of the sequence with as good or better a score. False discovery rate analysis was then applied to estimate a *q*-value for each position in the given sequences, and the motif occurrences with a *q*-value less than 0.05 were accepted as statistically significant. The Chi-square test was performed for transcription factor binding site distributions in different gene clusters at the significance level of α = 0.05.

### 2.10. ChIP-Seq Analysis

Chromatin immunoprecipitation was performed using the Pierce Agarose ChIP kit (ThermoFisher, Waltham, MA, USA) following the manufacturer’s instructions. Briefly, an amount of 60 mg of jejunum sample was put into cold PBS, sheared into small pieces, and fixed with formaldehyde. The crosslinked sample was digested with Micrococcal Nuclease for chromatin fragmentation. Immunoprecipitation was conducted by overnight incubating the chromatin fragments with 5 ug H3K4me3 antibody (Abcam, Shanghai, China) at 4 °C on a rocking platform. A portion of the digested chromatin without the immunoprecipitation procedure was used as the input. The recovered DNA was used for library construction and sequenced on an Illumina PE150 Hiseq platform. Raw reads were trimmed to remove the low-quality reads using the skewer software [19]. Clean reads were aligned to the porcine reference genome (Sscrofa11.1) using BWA [20]. Peaking calling of the ChIP samples was conducted by comparison with the reads of input using MACS2 [21], with the significance level of *p* < 0.005. Fold changes were calculated for the peak regions by comparing the H3K4me3 peaks of PEDV-infected samples with controls.

## 3. Results

### 3.1. PEDV Detection and Histopathological Analysis

**Figure 1:**
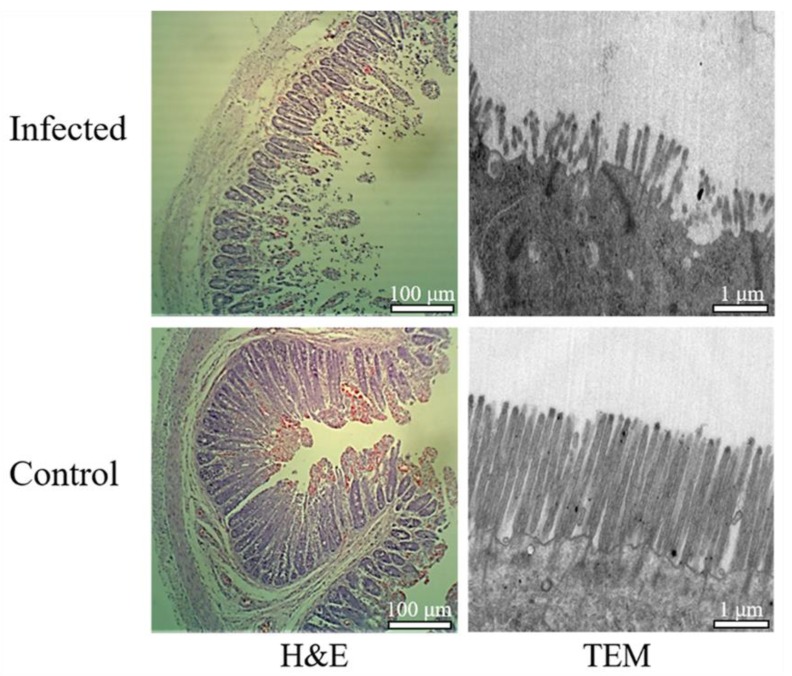
Histopathological analyses of the jejunum tissues derived from porcine epidemic diarrhea virus (PEDV)-infected and control animals. H&E: hematoxylin and eosin staining, TEM: Transmission electron microscopic.

The qRT-PCR assay was performed to detect PEDV in the intestinal contents and feces samples. The M gene sequence of PEDV was only amplified in the infected samples and the sequence was consistent with classic PEDV strain CV777 deposited in the NCBI database (Figure S1). Pathological changes were also observed in PEDV-infected jejunum samples. Compared to the controls, the infected samples demonstrated obvious lesions including villi shortening, atrophy and fusion, abnormity and desquamation of the epithelial cells, and an irregular striated border (Figure 1). The PEDV-infected and control jejunum samples were used for further transcriptomic analyses.

### 3.2. Overview of the RNA-Seq Data

Using the Illumina PE150 RNA-seq platform, sequencing of the eight jejunum samples generated a total of approximately 424.06 million raw reads, from which 412.42 million clean reads were obtained, with an average of 51.55 million clean reads (7.73 Gb of sequence) per sample (Table S2). Through alignment with the pig assembly Sscrofa11.1, 369.56 million reads (89.6%) were mapped to the genome, of which 353.87 million reads (85.8%) were uniquely mapped (Table S3). Genomic distribution analysis demonstrated that on average 90.7% (range, 86.4% to 92.5%) of the mapped reads were located in the exons, 5.3% (range, 3.4% to 9.4%) fell into the introns, and 4% (range, 3.6% to 4.3%) were mapped to intergenic regions (Figure S2). We further performed a Pearson correlation analysis based on the gene expression levels between the samples within each group. The results showed an average correlation coefficient of R^2^ = 0.93 between the infected samples and an average correlation coefficient of R^2^ = 0.94 between the control samples, which indicated the high reproducibility and reliability of our experimental data (Figure S3).

### 3.3. Differential Gene Expression Analysis

**Figure 2:**
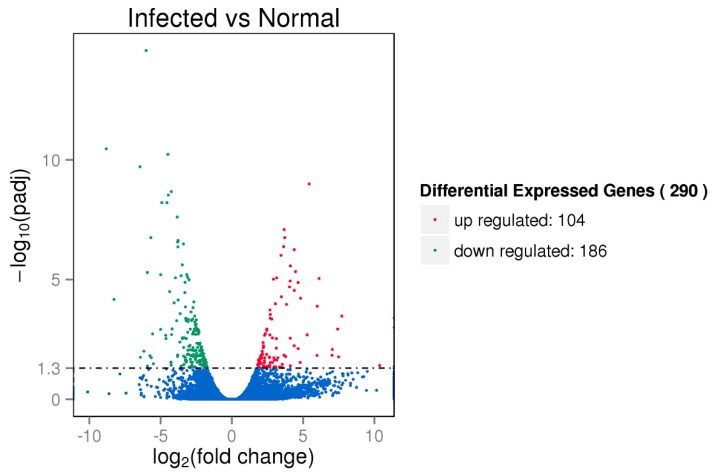
Volcano plot of differentially expressed genes. The red and green dots indicate the differentially up-regulated and down-regulated genes (adjusted *p* < 0.05), respectively.

To reveal the transcriptomic differences between the infected and control samples, we performed differential gene expression analysis. In total, 290 differential expression genes were identified (adjusted *p <* 0.05), with 104 up-regulated genes and 186 down-regulated genes (Figure 2). The differential expression genes are listed in Table S4. The K-means clustering analysis was then conducted to classify genes with similar biological functions. The differential expression genes were clustered into six clusters (clusters 1 to 6), which consisted of 3, 9, 26, 157, 66, and 29 genes respectively (Figure S4; Table S5). To gain insight into the biological functions of differential expression genes, we performed gene ontology annotations and found that these genes were significantly enriched in chemokine activity, chemokine receptor binding, and G-protein coupled receptor binding (Table S6). Pathway analysis revealed the genes were significantly enriched in pathways such as linoleic acid metabolism, mineral absorption, and galactose metabolism (Table S7). 

To gain further insight into the associations of the differential expression genes with PEDV infections, we compared these genes with the findings of proteomic analyses of PEDV-infected samples at the individual and cellular level [7,8,9]. In total, 19 differential expression genes were also found to show differential expression at the protein level (Table 1). Among these genes, 16 genes showed a consistent expression trend at mRNA and protein levels, of which nine genes (2’-5’-oligoadenylate synthetase like, *OASL*; annexin A4, *ANXA4*; hexokinase 2, *HK2*; ISG15 ubiquitin like modifier, *ISG15*; purine nucleoside phosphorylase, *PNP*; glucosaminyl (N-acetyl) transferase 3, *GCNT3*; nucleophosmin/nucleoplasmin 3, *NPM3*; uridine phosphorylase 1, *UPP1*; anterior gradient 2, *AGR2*) were up-regulated and seven genes (apolipoprotein C3, *APOC3*; apolipoprotein A1, *APOA1*; ectonucleoside triphosphate diphosphohydrolase 5, *ENTPD5*; cytochrome P450, family 2, subfamily J, polypeptide 34, *CYP2J34*; epoxide hydrolase 1, *EPHX1*; catalase, *CAT*; glycerophosphodiester phosphodiesterase domain containing 2, *GDPD2*) were down-regulated. The remaining three genes (glycerol-3-phosphate dehydrogenase 1, *GPD1*; ATP binding cassette subfamily D member 3, *ABCD3*; enoyl-CoA hydratase and 3-hydroxyacyl CoA dehydrogenase, *EHHADH*) displayed different patterns between the protein and mRNA expression levels (Table 1). In addition, the two genes hook microtubule tethering protein 2 (*HOOK2*) and solute carrier family 40 member 1 (*SLC40A1*) showing differential down-regulation in our results were also previously detected to show genomic differences between dead and recovered piglets after porcine epidemic diarrhea outbreaks [22].

**Figure 3:**
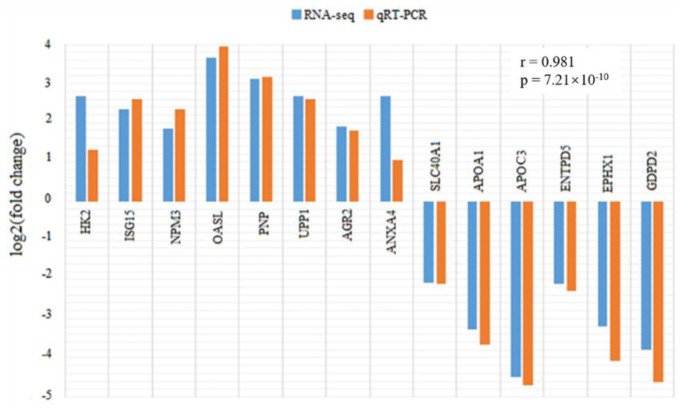
Comparison of expression fold changes in 14 differential expression genes (*HK2*, *ISG15*, *NPM3*, *OASL*, *PNP*, *UPP1*, *AGR2*, *ANXA4*, *SLC40A1*, *APOA1*, *APOC3*, *ENTPD5*, *EPHX1*, and *GDPD2*) between RNA-seq and qRT-PCR data. Fold changes are expressed as the ratio of gene expression levels in PEDV-infected and control groups. The blue and orange bars indicate the RNA-seq and qRT-PCR data, respectively. *HK2*: hexokinase 2; *ISG15*: ISG15 ubiquitin like modifier; *NPM3*: nucleophosmin/nucleoplasmin 3; *OASL*: 2’-5’-oligoadenylate synthetase like; *PNP*: purine nucleoside phosphorylase; *UPP1*: uridine phosphorylase 1; *AGR2*: anterior gradient 2; *ANXA4*: annexin A4; *SLC40A1*: solute carrier family 40 member 1; *APOA1*: apolipoprotein A1; *APOC3*: apolipoprotein C3; *ENTPD5*: ectonucleoside triphosphate diphosphohydrolase 5; *EPHX1*: epoxide hydrolase 1; *GDPD2*: glycerophosphodiester phosphodiesterase domain containing 2.

To validate the differential expression analysis of RNA-seq data, 14 genes including *HK2*, *ISG15*, *NPM3*, *OASL*, *PNP*, *UPP1*, *AGR2*, *ANKA4*, *SLC40A1*, *APOA1*, *APOC3*, *ENTPD5*, *EPHX1*, and *GDPD2* were selected to detect their expression differences between the infected and control groups using qRT-PCR (Figure 3). The expression patterns of the tested genes showed high concordance with the differential analysis results of RNA-seq, with a correlation coefficient of 0.981 (*p* = 7.21 × 10^−10^), indicating the high accuracy and reliability of the RNA-seq analysis. 

### 3.4. Annotation of Differentially Expressed Transcription Factors

**Figure 4:**
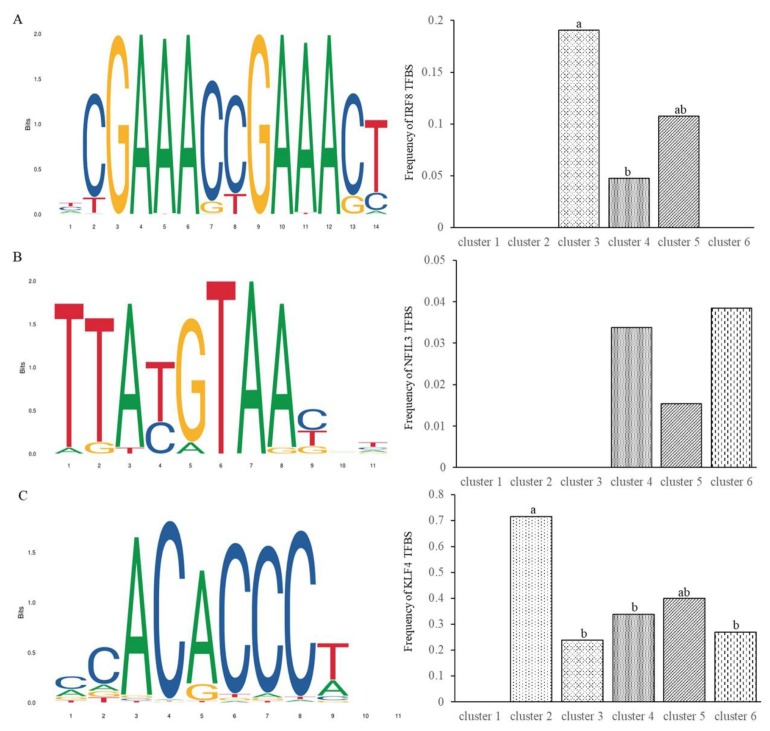
Occurrences of putative transcription factor binding sites (TFBSs) motifs of transcription factors (**A**) IRF8, (**B**) NFIL3, and (**C**) KLF4 in the promoter regions of various gene clusters. Bars with different letters (a, b, ab) indicate significant differences at the significance level of α = 0.05.

It has been shown that the over-representation of transcription factor binding sites (TFBSs) is associated with transcriptional regulation of unique gene clusters [23]. We thus conducted transcription factor annotation analysis of the differential expression genes. After comparative analyses with the AnimalTF 2.0 database [17], a total of ten transcription factors in 6 families, 15 transcription cofactors, and two chromatin remodeling factors of pigs were identified (Table 2). Among these transcription factors, interferon regulatory factor 8 (IRF8), nuclear factor, interleukin 3 regulated (NFIL3), and Kruppel like factor 4 (KLF4) have been identified to play important roles in pathogenic infections and host responses [24,25,26]. We, therefore, analyzed the occurrences of their TFBSs motifs in the promoter regions of differential expression gene clusters. The prevalence of predicted TFBSs displayed significant differences between the genes from different clusters. In clusters 3, the putative TFBSs of IRF8 demonstrated a higher frequency than in cluster 4 (Figure 4A). The genes in clusters 3 and 5 were obviously up-regulated, while those in cluster 4 were obviously down-regulated, suggesting that IRF8 has a role in transcriptional up-regulation of genes relevant to PEDV infections. There were no occurrences of the putative TFBSs in clusters 1, 2, and 6. For transcription factor NFIL3, the putative TFBSs were detected at a frequency lower than 4% in all gene clusters and there were no remarkable differences between these gene clusters (Figure 4B). In cluster 2, the putative TFBSs of KLF4 exhibited a frequency of 71.4%, which was significantly higher than that in other gene clusters (Figure 4C). Particularly, the log2 fold changes (ranging from 5.3 to 10.9) of genes in cluster 2 were relatively higher, which indicates the potentially significant effects of KLF4 on up-regulating the expression of these genes. The full set of significant motif occurrences is listed in Table S8.

### 3.5. Alterations in H3K4me3 Patterns in the PEDV-Infected Jejunum

**Figure 5:**
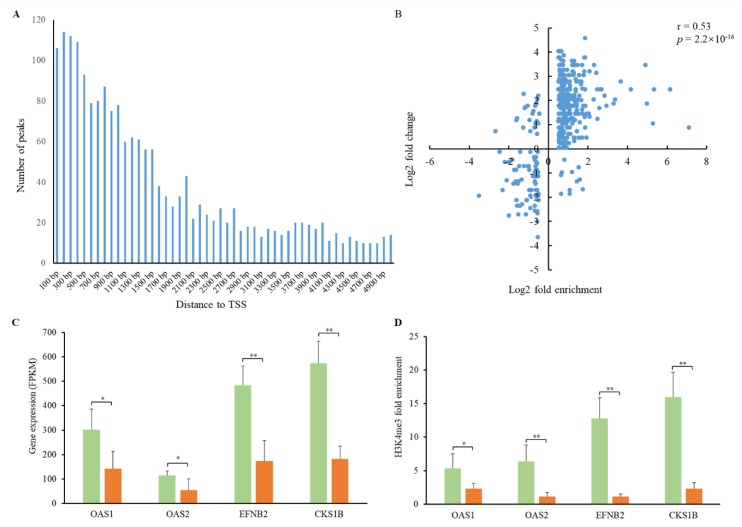
H3K4me3 peak distribution and associations with gene expression. (**A**) Distribution of H3K4me3 peaks with the distance to the transcription start site (TTS). (**B**) Associations between H3K4me3 enrichment and gene expression. Pearson’s coefficient r = 0.53, *p* = 2.2 × 10^−16^. (**C**) Expression of the *OAS1*, *OAS2*, *EFNB2*, and *CKS1B* genes in PEDV-infected and control samples. (**D**) H3K4me3 fold enrichment of the *OAS1*, *OAS2*, *EFNB2*, and *CKS1B* genes in PEDV-infected and control samples. Green and oranges bars represent PEDV-infected and control samples, respectively. * *p* < 0.05, ** *p* < 0.01.

To investigate the alterations of H3K4me3 patterns in the PEDV-infected jejunum, we performed global mapping of H3K4me3 modification using ChIP-seq. The H3K4me3 peak distributions near the transcription start site (±5 kb) of the annotated pig genes were analyzed. The H3K4me3 peaks showed higher enrichment in the regions close to the transcription start sites (Figure 5A). Compared to the controls, 1885 peaks associated with 1723 genes were identified (Table S9). We then performed GO analysis for the genes associated with H3K4me3 modification. These genes were highly enriched in categories including regulation of cell morphogenesis involved in differentiation, cytosolic transport, and regulation of stress-activated MAPK cascade (Table S10). Integrative analysis of H3K4me3 enrichment and gene expression data of the PEDV-infected jejunum showed that most promoters of the up-regulated genes demonstrated higher H3K4me3 enrichment, and those of down-regulated genes displayed lower H3K4me3 enrichment (Figure 5B), indicating the associations of H3K4me3 with transcriptional activity. A subset of genes including *OAS1*, *OAS2*, *EFNB2*, and *CKS1B* involved in the immune response to viral infection showed a higher expression level (Figure 5C) and H3K4me3 enrichment (Figure 5D) in PEDV-infected samples, suggesting the role of H3K4me3 deposition in promoting their expression.

## 4. Discussion

In recent years, porcine epidemic diarrhea outbreaks have caused mass epidemics and threatened the pig industry worldwide. Previous studies mainly focused on the functions of the receptor aminopeptidase N gene during PEDV cell entry [27] and on proteomic changes in response to PEDV challenge [7,8,9]. As phenotypic alterations induced by pathogenic infections are usually tightly linked with marked changes in gene expression, we explored the differences in gene expression profiling and H3K4me3 distribution between PEDV-infected and normal jejunum samples for providing novel insights into PEDV-host interactions. We showed the down-regulation of the *SLC40A1* and *HOOK2* genes in the PEDV-infected animals. Genetic variants within these two genes were reported to be associated with PEDV resilience [22]. SLC40A1 is critical for intestinal iron absorption and metabolism homeostasis [28]. HOOK2 functions in multiple cellular processes including endosome/lysosome processing and polarized Golgi re-orientation [29]. Impairing intestinal ion transport is an important pathophysiologic mechanism involved in viral diarrhea in pigs and iron homeostasis is necessary for intestine functions [30,31]. Furthermore, the endosome/lysosome system participates in the regulation of viral infections [32]. Alterations in the expression of these two genes indicated their possible involvement in PEDV pathogenesis, while the mechanisms for their functions and transcriptional regulation remain to be further explored. 

Intriguingly, we observed significant up-regulation of the *OASL* gene in the infected samples, consistent with the changes in OASL protein expression shown by previous studies on proteomic analyses of PEDV-infected piglets and IPEC-J2 cells [8,9]. These highlighted an important role of OASL in responses to PEDV infections. OASL has been shown to be a crucial regulator in controlling antiviral innate immunity. It is rapidly induced by viral infections through interferon regulatory factor 3 and by interferon signaling [33], and exerts antiviral activity by enhancing signaling of the RIG-I RNA sensor [34]. In addition, previous epidemiological studies have revealed the associations of SNPs in the human *OASL* gene with altered susceptibility to West Nile and hepatitis C virus infections [35,36]. The *OASL* gene has also been shown to be obviously up-regulated in response to porcine virus infections such as swine influenza virus and fever virus [37,38]. In addition, our comparative analysis identified other genes related to host-pathogen interactions such as ISG15 that is an antiviral molecule with activity against both DNA and RNA viruses [39] and AGR2 that regulates the production of intestinal mucus [40]. These findings provided us with an important clue to identify genetic markers and develop therapeutic drug targets to combat porcine viral diseases. 

It has been demonstrated that PEDV infections could subvert type I interferon response [41]. Activation of the interferon regulatory factors is essential for controlling the transcriptional activity of type I interferon genes and a large number of interferon-stimulated genes [42]. IRF8 has been characterized as an important modulator in response to various pathogenic infections such as the Epstein–Barr virus [25], herpes simplex virus 1 [43], and Helicobacter pylori [44]. Moreover, IRF8-dependent dendritic cells are crucial for the maintenance of intestinal T cell homeostasis [45]. Here we found that IRF8 showed a relatively higher frequency of putative TFBSs in the promoters of genes in the cluster 3, which includes the aconitate decarboxylase 1 (*ACOD1*) gene whose expression can be induced by bacterial or viral infections [46,47]. Particularly, in the promoter of the *ISG15* gene, four putative TFBSs of IRF8 were predicted. These genes were differentially up-regulated, which indicated the potentially positive regulatory role of IRF8 in response to PEDV infections by targeting the promoter region of the genes. In addition, increased prevalence of KLF4 binding motifs was predicted in the promoters of genes in cluster 2. KLF4 plays crucial roles in regulating viral infections and host immune responses by binding to the promoters of immune genes such as interferon β (*IFNβ*) [48], Z transactivator (*BZLF1*), and R transactivator (*BRLF1*) [49]. Our data suggested that KLF4 is likely to be involved in modulating PEDV infections by affecting the expression of target genes. Nonetheless, identification of transcription factor motif by motif-based sequence analysis only denotes the potential for the physical binding of transcription factors to the regulatory regions, which do not necessarily mean the biological functions. Mechanistic studies of the roles of the identified transcription factors in gene expression need to be further investigated.

H3K4me3 is an active histone modification mark that positively associates with gene expression [50]. It is reported that pathogenic viruses can control the global interferon-stimulated gene responses through altered histone modifications [51]. We herein identified the increased H3K4me3 levels at the promoter regions of the *OAS1* and *OAS2* genes that serve as antiviral effectors by repressing all steps of viral replication [52]. Expression of the *OAS2* and *OAS2* genes was found to be epigenetically regulated in response to pathogenic infections [53,54]. Increased expression of these two genes was also detected in the PEDV-infected jejunum samples, which indicated that the expression of the two genes in intestinal epithelial cells may be controlled by H3K4me3 modifications in response to PEDV infections.

## 5. Conclusions

In conclusion, this study presented the first report of changes in H3K4me3 enrichment and gene expression patterns associated with PEDV infection in pigs. A subset of genes involved in the regulation of PEDV infections was found to be enriched for H3K4me3. Furthermore, our findings of the putative TFBSs prevalence of transcription factors IRF8 and KLF4 in the promoter regions of different gene clusters provided us a useful clue for clarifying the mechanisms modulating the differential expression of gene clusters. These findings may aid in the detection of key regulators and genetic markers resistance to PEDV infections and in the development of diagnostic and therapeutic strategies to fight porcine epidemic diarrhea.

## Figures and Tables

**Table 1:** Overlapped genes between our differential expression analysis and previously identified proteomic expression changes in PEDV-infected and control samples.

Gene Symbol	Log2 (Fold Change)	Ratio (Infected/Control)
*APOC3*	−4.4897	0.2225 ^a^
*OASL*	3.6703	3.8880 ^a^/2.256 ^b^
*APOA1*	−3.2846	0.4873 ^a^
*ANXA4*	2.6891	1.8863 ^a^
*HK2*	2.6892	2.7343 ^a^
*ISG15*	2.3519	2.4863 ^a^
*PNP*	3.1261	2.1248 ^a^
*GCNT3*	2.2081	2.0617 ^a^
*GPD1*	−3.0855	1.8292 ^a^
*ENTPD5*	−2.0974	0.7424 ^a^
*CYP2J34*	−1.9876	0.7187 ^a^
*ABCD3*	−2.2627	1.2734 ^a^
*EPHX1*	−3.1905	0.5197 ^a^
*CAT*	−1.8051	0.9870 ^a^
*NPM3*	1.8512	2.6182 ^a^
*EHHADH*	−1.8011	1.3862 ^a^/1.399 ^b^
*GDPD2*	−3.7889	0.735 ^b^
*UPP1*	2.6795	1.348 ^b^
*AGR2*	1.8933	1.334 ^b^

The superscripts a and b represent the proteomic analysis results from previous studies [8] and [9], respectively. Log2 fold change smaller than zero represents down-expression of the genes and greater than zero represents up-regulation. The ratio value smaller than 1 represents down-expression of the proteins and greater than 1 represents up-regulation. *APOC3*: apolipoprotein C3; *OASL*: 2’-5’-oligoadenylate synthetase like; *APOA1*: apolipoprotein A1; *ANXA4*: annexin A4; *HK2*: hexokinase 2; *ISG15*: ISG15 ubiquitin like modifier; *PNP*: purine nucleoside phosphorylase; *GCNT3*: glucosaminyl (N-acetyl) transferase 3; *GPD1*: glycerol-3-phosphate dehydrogenase 1; *ENTPD5*: ectonucleoside triphosphate diphosphohydrolase 5; *CYP2J34*: cytochrome P450, family 2, subfamily J, polypeptide 34; *ABCD3*: ATP binding cassette subfamily D member 3; *EPHX1*: epoxide hydrolase 1; *CAT*: catalase; *NPM3*: nucleophosmin/nucleoplasmin 3; *EHHADH*: enoyl-CoA hydratase and 3-hydroxyacyl CoA dehydrogenase; *GDPD2*: glycerophosphodiester phosphodiesterase domain containing 2; *UPP1*: uridine phosphorylase 1; *AGR2*: anterior gradient 2.

**Table 2:** Identification of transcription factors from differential expression genes.

Transcription Factor Family	Gene Symbol
zf-C2H2	*KLF4, PRDM16, ZNF852, ZKSCAN7*
TF-bZIP	*NFIL3, DBP*
THR-like	*NR1I3*
IRF	*IRF8*
ETS	*ETV4*
Homeobox	*HOXD1*
Transcription cofactors	*HELZ2, IL31RA, ADRB2, YWHAB, CDK2, MAPK9, RBM39, PPARGC1, WNT5A, ASB4, LOC100514979, SCAND1, NPM1, MAP3K10, USP27X*
Chromatin remodeling factors	*JAK2, NPM2*

zf-C2H2: zinc finger Cys2His2-like; TF-bZIP: transcription factor basic leucine zipper; THR-like: threonine like; IRF: interferon regulatory factor; ETS: transcription factor Ets; *KLF4*: Kruppel like factor 4; *PRDM16*: PR/SET domain 16; *ZNF852*: zinc finger protein 852; *ZKSCAN7*: zinc finger with KRAB and SCAN domains 7; *NFIL3*: nuclear factor, interleukin 3 regulated; *DBP*: D-box binding PAR bZIP transcription factor; *NR1I3*: nuclear receptor subfamily 1 group I member 3; *IRF8*: interferon regulatory factor 8; *ETV4*: ETS variant transcription factor 4; *HOXD1*: homeobox D1; *HELZ2*: helicase with zinc finger 2; *IL31RA*: interleukin 31 receptor A; *ADRB2*: adrenoceptor beta 2; *YWHAB*: tyrosine 3-monooxygenase/tryptophan 5-monooxygenase activation protein beta; *CDK2*: cyclin dependent kinase 2; *MAPK9*: mitogen-activated protein kinase 9; *RBM39*: RNA binding motif protein 39; *PPARGC1*: PPARG coactivator 1 alpha; *WNT5A*: Wnt family member 5A; *ASB4:* ankyrin repeat and SOCS box containing 4; *LOC100514979*: tripartite motif-containing protein 65-like; *SCAND1*: SCAN domain containing 1; *NPM1*: nucleophosmin 1; *MAP3K10*: mitogen-activated protein kinase kinase kinase 10; *USP27X*: ubiquitin specific peptidase 27 X-linked; *JAK2*: Janus kinase 2; *NPM2*: nucleophosmin/nucleoplasmin 2.

## Supplementary Materials

The following are available online at https://www.mdpi.com/2076-2615/9/8/523/s1, Table S1: Primer sequences used for qRT-PCR, Table S2: Statistics for RNA-seq data of all samples, Table S3: Summary of RNA-seq mapping results, Table S4: Differentially expressed genes between PEDV-infected and control groups, Table S5: List of genes in different gene clusters, Table S6: Gene Ontology analyses for differentially expressed genes, Table S7: Pathway analyses for differentially expressed genes, Table S8: The full set of significant motif occurrences, Table S9: List of gene associated with H3K4me3 enrichment, Table S10: Gene Ontology analyses for genes associated with H3K4me3 modifications, Figure S1: Amplification of PEDV M gene and PCR sequencing, Figure S2: Percent of reads mapped to the genomic regions, Figure S3: Pearson correlation coefficients between the samples within each group, Figure S4: Clusters of the differential expression genes by K-means clustering analysis.
