# Supplementary material for: Global Mapping of H3K4 Trimethylation (H3K4me3) and Transcriptome Analysis Reveal Genes Involved in the Response to Epidemic Diarrhea Virus Infections in Pigs
Source: Animals (Basel). 2019 Aug 2;9(8):523. doi: 10.3390/ani9080523 (PMC6719071; doi:10.3390/ani9080523)
Supplement: Supplementary file 1 [file animals-09-00523-s001.pdf]

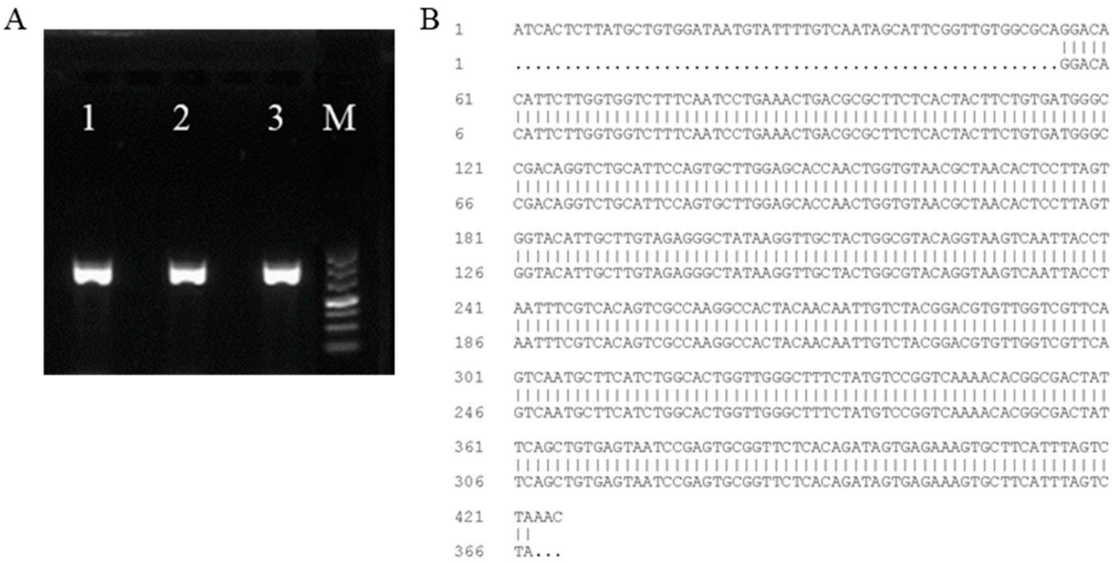

Figure S1. Amplification of PEDV M gene and PCR sequencing. (A) PCR products of PEDV M gene. Lanes 1, 2, and 3 represent the PCR products of PEDV-infected samples; M: 50 bp DNA ladder. (B) Alignment of the M gene sequence (upper line) and PCR product sequence (lower line).

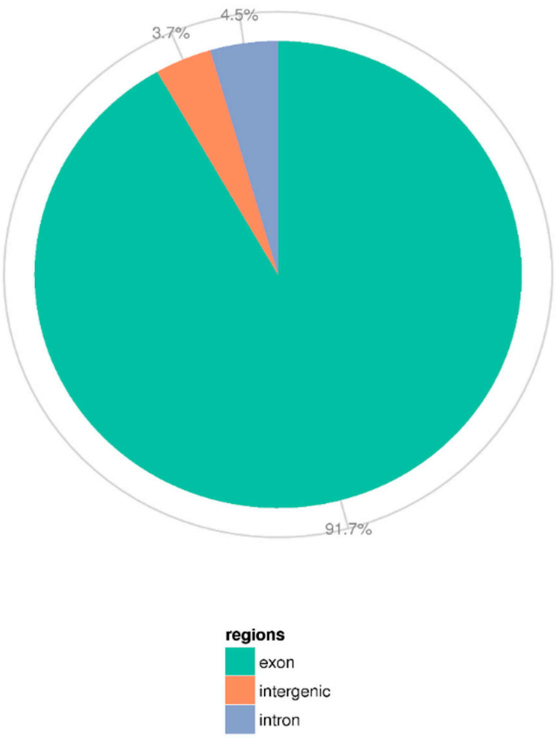

Figure S2. Percent of reads mapped to the genomic regions. Shown is an example of alignment analysis results of one randomly selected sample.

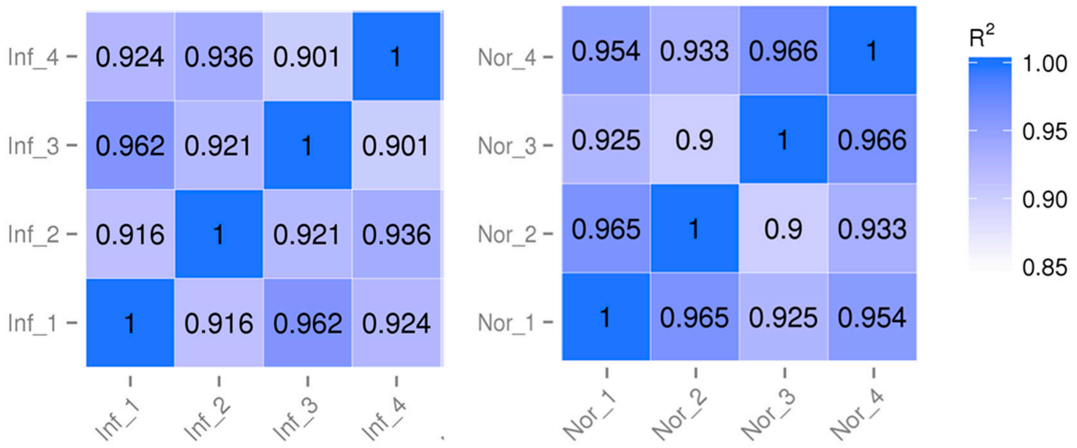

Figure S3. Pearson correlation coefficients between the samples within each group. Correlation analysis was performed based on the log10 (FPKM+1) that represent the gene expression levels. Inf_1, Inf_2, Inf_3, and Inf_4 denote the infected samples, and Nor_1, Nor_2, Nor_3, and Nor_4 denote the control samples.

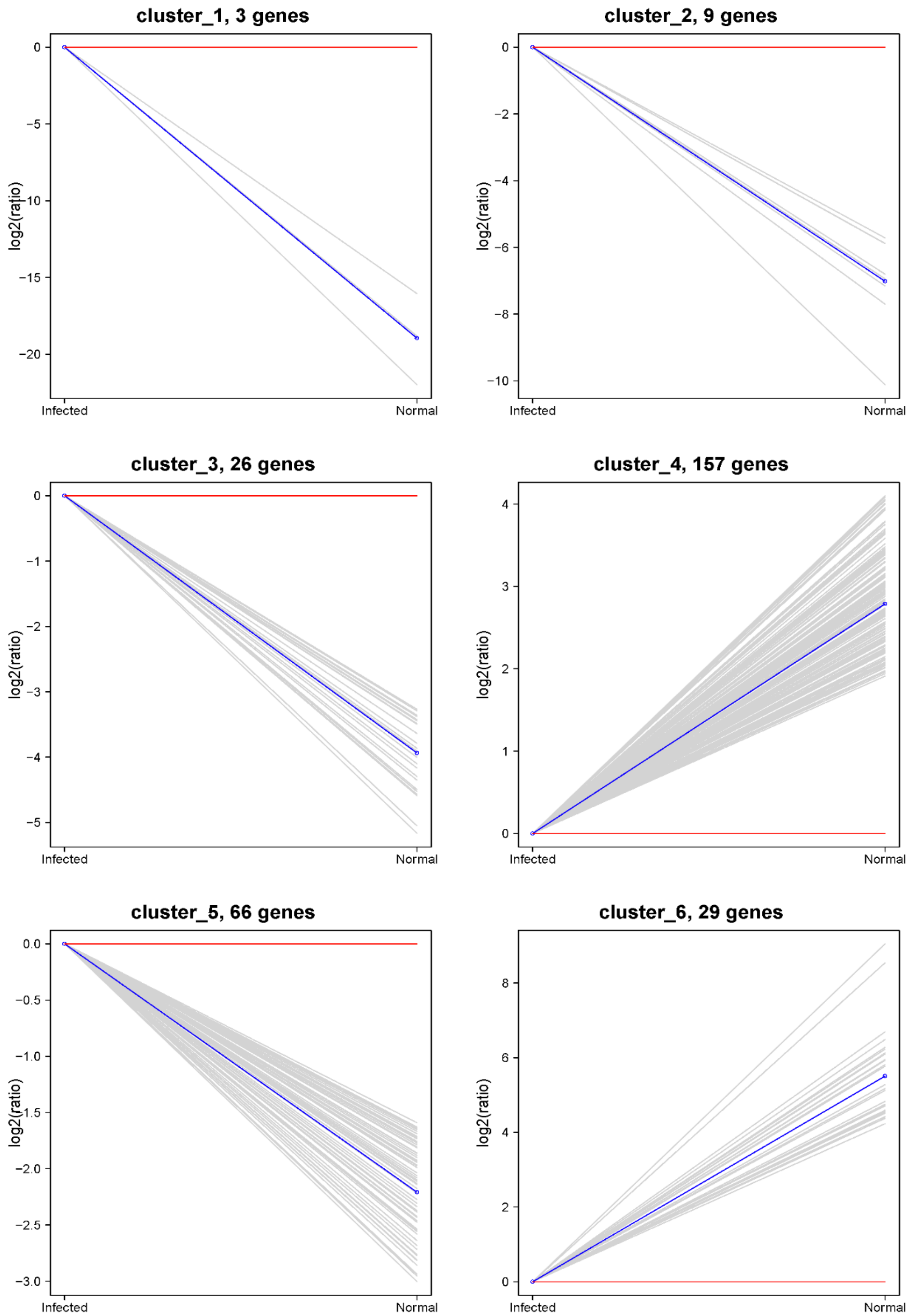

Figure S4. Clusters of the differential expression genes by K-means clustering analysis. The genes were clustered into six clusters (cluster 1 to cluster 6) which consisted of 3, 9, 26, 157, 66, and 29 genes, respectively. The gray lines denote the relative gene expression level between the infected and control, and the blue line denotes the means of expression level of all genes in the cluster.

Table S1. Primer sequences used for qRT-PCR.

Gene symbol	Forward (5'-3')	Reverse (5'-3')	Annealing temperature (℃)	Reference/Accession
APOC3	AGGACACCTCCCTTCTGGAC	GTCGGTGAACTTGCCCTTGA	60	[1]
ANXA4	GCGAAGAGCCATGAAGGGA	GCCACCAGCGGACAGAGAC	60	NM_001167639.1
APOA1	GTTCTGGGACAACCTGGAAA	GCTGCACCTTCTTCTTCACC	60	[2]
AGR2	TGCTTGTGGCCCTCTCTTAT	GGGTTTGTTGCTTGTCTTGG	60	NM_001244968.1
ENTPD5	CTTGGAAAACTCAGGGATGGA	TGGGTGGAGACAAATGTAGGA	60	XM_021099509.1
EPHX1	CTGCTGACAGTCATCATGCTCTACT	GTGGACCTTGATCGCCTCAT	60	NM_214355.1
GDPD2	CCACAGCCCCATTCCTTC	GCCCCTCGGTGTCCCATC	60	XM_003135152.4
HK2	CATCCGCTTCCTCCGCTCT	CCTCGCTCCATTTCCACCT	60	NM_001122987.1
NPM3	GCTCCAACCACCTGTAACCT	ACTCCCCTCTTCTTCACTCT	60	XM_003359343.4
OASL	CCTATGGCTACAGATGGGAC	GGACTGGGCTCTTGTTGTT	60	[3]
PNP-2	ACAGTTTGTCTCCATCCTTA	AATCTTCCTCTCCTTTTTGC	60	XM_013989064.2
ISG15	GATGCTGGGAGGCAAGGA	CAGGATGCTCAGTGGGTCTCT	60	[4]
SLC40A1	CTGGAATAATGGGAACTGTGG	TGGCTCTGTCTGAATGAACCT	60	XM_003483701.4
UPP1	CGGCTATGTTTGGAGATGTG	ATGATGGCAATGGAGGGAAT	60	XM_021078790.1
GAPDH	ACATCATCCCTGCTTCTACTGG	CTCGGACGCCTGCTTCAC	60	AF017079.1

References

[1] Ogłuszka, M.; Szostak, A.; Te Pas, M.F.W.; Poławska, E.; Urbański, P.; Blicharski, T.; Pareek, C.S.; Juszczuk-Kubiak, E.; Dunkelberger, J.R.;

Horbańczuk, J.O.; et al. A porcine gluteus medius muscle genome-wide transcriptome analysis: dietary effects of omega-6 and omega-3 fatty acids on biological mechanisms. *Genes Nutr.* **2017**, *12*, 4.
[2] Skovgaard, K.; Mortensen, S.; Boye, M.; Poulsen, K. T.; Campbell, F. M.; Eckersall, P.D.; Heegaard, P.M.H. Rapid and widely disseminated acute phase protein response after experimental bacterial infection of pigs. *Vet. Res.* **2009**, *40*, 336-336.
[3] Zheng, S.; Zhu, D.; Lian, X.; Liu, W.; Cao, R.; Chen, P. Porcine 2′, 5′-oligoadenylate synthetases inhibit japanese encephalitis virus replication in vitro. *J. Med. Virol.* **2016**, *88*, 760-768.
[4] Huang, J.; Zhao, S.; Zhu, M.; Wu, Z.; Yu, M. Sequence and expression analyses of porcine isg15 and isg43 genes. *Comp. Biochem. Physiol. B Biochem. Mol. Biol.* **2009**, *153*, 0-309.

Table S2. Statistics for RNA-seq data of all samples.

Sample name	Raw reads	Clean reads	Clean bases	Error rate(%)	Q20(%)	Q30(%)	GC content(%)
Inf_1	51964190	50110508	7.52G	0.02	96.36	91.25	55.72
Inf_2	51882108	50528298	7.58G	0.02	96.98	92.45	51.62
Inf_3	58170206	56681794	8.5G	0.02	96.81	92.16	52.9
Inf_4	49020254	47251880	7.09G	0.02	96.81	92.1	52.94
Nor_1	55848424	54458148	8.17G	0.01	97.44	93.53	54.6
Nor_2	54855556	53478346	8.02G	0.02	96.94	92.35	54.97
Nor_3	46307770	45274664	6.79G	0.02	96.99	92.42	50.97
Nor_4	56014286	54632550	8.19G	0.02	96.88	92.22	52.21

Inf_1, Inf_2, Inf_3, and Inf_4 represent the PEDV-infected samples; Nor_1, Nor_2, Nor_3, and Nor_4 represent the control samples.

Table S3. Summary of RNA-seq mapping results.

Sample name	Inf_1	Inf_2	Inf_3	Inf_4	Nor_1	Nor_2	Nor_3	Nor_4
Total reads	50110508	50528298	56681794	47251880	54458148	53478346	45274664	54632550
Total mapped	43558318 (86.92%)	45516333 (90.08%)	50162599 (88.5%)	42075049 (89.04%)	48510542 (89.08%)	47746114 (89.28%)	42220781 (93.25%)	49774246 (91.11%)
Multiple mapped	4365414 (8.71%)	1240103 (2.45%)	3767843 (6.65%)	1237691 (2.62%)	1522978 (2.8%)	1302712 (2.44%)	888181 (1.96%)	1361862 (2.49%)
Uniquely mapped	39192904 (78.21%)	44276230 (87.63%)	46394756 (81.85%)	40837358 (86.42%)	46987564 (86.28%)	46443402 (86.85%)	41332600 (91.29%)	48412384 (88.61%)
Read-1	19874852 (39.66%)	22297418 (44.13%)	23392841 (41.27%)	20626485 (43.65%)	23698386 (43.52%)	23398204 (43.75%)	20825946 (46%)	24384164 (44.63%)
Read-2	19318052 (38.55%)	21978812 (43.5%)	23001915 (40.58%)	20210873 (42.77%)	23289178 (42.77%)	23045198 (43.09%)	20506654 (45.29%)	24028220 (43.98%)
Reads map to '+'	19631733 (39.18%)	22094958 (43.73%)	23166487 (40.87%)	20386058 (43.14%)	23474911 (43.11%)	23200167 (43.38%)	20628114 (45.56%)	24162536 (44.23%)
Reads map to '-'	19561171 (39.04%)	22181272 (43.9%)	23228269 (40.98%)	20451300 (43.28%)	23512653 (43.18%)	23243235 (43.46%)	20704486 (45.73%)	24249848 (44.39%)
Non-splice reads	21007802 (41.92%)	23596762 (46.7%)	25775118 (45.47%)	22607173 (47.84%)	25722127 (47.23%)	26510122 (49.57%)	24744453 (54.65%)	27221483 (49.83%)
Splice reads	18185102 (36.29%)	20679468 (40.93%)	20619638 (36.38%)	18230185 (38.58%)	21265437 (39.05%)	19933280 (37.27%)	16588147 (36.64%)	21190901 (38.79%)

Inf_1, Inf_2, Inf_3, and Inf_4 represent the PEDV-infected samples; Nor_1, Nor_2, Nor_3, and Nor_4 represent the control samples.

Table S4. Differential expression genes between PEDV-infected and control groups.

Gene ID	Read count Infected	Read count Normal	Log2 Fold Change	P value	Adjusted P value	Gene Name
414380	7.062879945	458.674154	-6.0211	1.10E-19	2.71E-15	DIO1
403104	2.648180345	1196.14734	-8.8192	2.79E-15	3.45E-11	CYP2B22
406187	4502.053242	101144.3495	-4.4897	7.14E-15	5.87E-11	APOC3
110261001	1.517730265	132.2506364	-6.4452	3.15E-14	1.94E-10	LOC110261001
100523844	114.532665	2.664185773	5.4259	2.06E-13	1.02E-09	PIK3C2G
100515970	29.60257566	561.7352392	-4.2461	5.13E-13	2.11E-09	OSBP2
110255314	11.69745033	255.8717125	-4.4512	8.44E-13	2.98E-09	LOC110255314
100155204	8.234480785	193.2503158	-4.5526	2.25E-12	6.16E-09	ZBBX
100157319	14.79516526	449.6201503	-4.9255	2.09E-12	6.16E-09	SDR16C5
100738360	46.09527069	655.6525309	-3.8302	9.90E-12	2.44E-08	PDIA2
595119	2119.218907	166.4622486	3.6703	3.61E-11	8.10E-08	OASL
100525396	536.8132439	41.11941225	3.7065	9.37E-11	1.78E-07	LOC100525396
397677	87.85229669	4517.724589	-5.6844	9.18E-11	1.78E-07	FOLH1B
100628018	600.3133545	8232.409101	-3.7775	1.31E-10	2.31E-07	SLC46A1
100623097	542.0145186	7576.734489	-3.8052	1.62E-10	2.67E-07	SLC30A10
100156419	145.5578175	1534.254198	-3.3979	2.11E-10	3.26E-07	HOMER2
100510974	413.2052509	33.33126825	3.6319	2.91E-10	4.23E-07	COL28A1
100516309	40.90502095	565.3841177	-3.7889	3.12E-10	4.27E-07	GDPD2
100156489	95.62438278	4.590001807	4.3808	4.33E-10	5.63E-07	TGM1
100515798	542.8801212	49.54044244	3.454	7.87E-10	9.71E-07	ARL14
100517307	29.602324	331.0605854	-3.4833	2.07E-09	2.43E-06	SLC6A4
100157318	739.5415214	43.02581697	4.1034	2.40E-09	2.69E-06	APOD
733625	791.4893522	35.82577461	4.4655	4.33E-09	4.65E-06	SFN
100627655	0.82490317	50.37163329	-5.9322	4.89E-09	5.03E-06	LOC100627655
397373	176.0306336	1585.765997	-3.1713	6.32E-09	6.24E-06	MYO7A
100620627	20.42009613	649.7789361	-4.9919	6.64E-09	6.31E-06	LOC100620627
692188	61.12237917	722.9782049	-3.5642	7.55E-09	6.90E-06	APOM
110256150	137.9489397	1184.956602	-3.1026	9.20E-09	8.11E-06	KCNJ12
100153752	14.88519008	232.5892431	-3.9658	1.03E-08	8.50E-06	CLDN10
106505478	942.1261064	106.2673261	3.1482	1.00E-08	8.50E-06	LOC106505478
733608	7657.057496	1013.129757	2.918	1.25E-08	9.65E-06	S100A6
100125551	169.7073795	1358.534046	-3.0009	1.37E-08	1.03E-05	SLC2A8

106505337	75.50729955	4.436794567	4.089	1.58E-08	1.15E-05	LOC106505337
397691	65343.70832	636753.8988	-3.2846	1.85E-08	1.31E-05	APOA1
100738704	82.35121334	3.291872103	4.6448	1.93E-08	1.32E-05	TNIP3
100737113	47.98680973	2.298964935	4.3836	4.39E-08	2.85E-05	LOC100737113
100126844	118.5235199	2440.529245	-4.3639	4.99E-08	3.16E-05	GATM
100512452	1197.402086	107.4162796	3.4786	8.69E-08	5.23E-05	GALNT12
100625897	5384.632347	34459.373	-2.678	1.55E-07	8.52E-05	LOC100625897
100625192	14.23478634	236.1401625	-4.0522	1.73E-07	9.31E-05	GCKR
100156922	282.9634985	34.03812745	3.0554	1.96E-07	0.0001032	MAT1A
100521889	56.32759432	3.976850743	3.8241	2.11E-07	0.00010851	ADGRE1
414904	342.7626871	5.410987817	5.9852	2.58E-07	0.00012984	CXCL2
110257275	11.20839585	107.368773	-3.2599	2.81E-07	0.00013852	LOC110257275
100134962	261.0502181	1723.426346	-2.7229	3.06E-07	0.00014822	PHYH
100625277	2427.471387	378.9067256	2.6795	3.90E-07	0.00018496	UPP1
100520068	40.19530006	293.3411389	-2.8675	4.84E-07	0.00022522	PCDH12
100156404	50.66066643	625.7451999	-3.6266	5.82E-07	0.00026583	PGF
100312960	12614.08746	1955.88924	2.6891	6.41E-07	0.00028767	ANXA4
100518950	601.4062675	2.861991888	7.7152	7.64E-07	0.00033427	FGF19
100521912	143.2750411	871.5852239	-2.6049	7.72E-07	0.00033427	WNT11
100739839	13.82622965	130.4343503	-3.2378	1.01E-06	0.00041396	SPATC1
494561	391.8121505	60.74969244	2.6892	9.90E-07	0.00041396	HK2
100135678	169.3444522	24.37646512	2.7964	1.12E-06	0.00044696	PDE6C
100512737	1010.480369	6784.614331	-2.7472	1.11E-06	0.00044696	SLC13A2
100514218	11.73876786	96.81290643	-3.0439	1.16E-06	0.00045493	GJC2
100158243	340.4624586	1916.692834	-2.4931	1.26E-06	0.00048441	SLC16A3
102161066	21.82008332	155.6475917	-2.8346	1.34E-06	0.00050976	LOC102161066
100516363	40.1712932	370.2857648	-3.2044	1.40E-06	0.00051999	NAT8
100626731	12.4038322	104.9812892	-3.0813	1.57E-06	0.00057029	LOC100626731
100524747	58.7583808	354.3702728	-2.5924	1.64E-06	0.00058677	DBP
100516578	145.1269753	821.0362954	-2.5001	2.09E-06	0.00073866	CCDC80
100151843	169.9459462	1012.457821	-2.5747	2.19E-06	0.00076002	SNTA1
110262031	93.41387646	539.1338626	-2.5289	2.79E-06	0.00095486	LOC110262031
397207	41.59337535	0	Inf	3.00E-06	0.0010156	HAMP
100155341	101.8776285	587.5995942	-2.528	3.38E-06	0.001129	NEIL1

100144620	176.31037	3233.62509	-4.197	3.55E-06	0.0011539	AQP10
100522927	189.4055852	1071.315422	-2.4998	3.55E-06	0.0011539	CGREF1
100153155	14.1703251	107.1046604	-2.9181	3.81E-06	0.0011702	XCL1
100155893	526.1751633	96.11947137	2.4526	3.68E-06	0.0011702	ARG2
100157662	38.09419094	0.221801735	7.4242	3.77E-06	0.0011702	HOXD1
106505853	29.10957067	179.5889912	-2.6251	3.84E-06	0.0011702	LOC106505853
397358	1396.869704	7341.483186	-2.3939	3.72E-06	0.0011702	SLC9A3
100171400	34.08652859	1093.797307	-5.004	3.99E-06	0.0012014	TRPV6
110256043	976.0847854	177.5844433	2.4585	4.46E-06	0.001327	LOC110256043
100523209	592.8822791	2978.985942	-2.329	4.59E-06	0.0013497	COL7A1
100151790	243.3490291	1367.727192	-2.4907	4.65E-06	0.0013506	PMM1
100513308	965.6841669	6276.514351	-2.7003	5.22E-06	0.0014994	TM4SF5
100624476	474.7413285	2327.982142	-2.2939	5.39E-06	0.0015297	PRDM16
100157774	413.0628608	2098.418441	-2.3449	5.55E-06	0.001557	SLC25A27
100523600	76.66483625	658.1421013	-3.1018	5.72E-06	0.0015865	PCP4L1
100145895	10966.67191	2148.166602	2.3519	6.58E-06	0.0018039	ISG15
100519006	2.533973993	119.443043	-5.5588	6.85E-06	0.0018568	DUSP9
100515460	19.57145	132.0847003	-2.7546	7.09E-06	0.0019025	USP27X
100152247	509.9493411	2640.159201	-2.3722	7.25E-06	0.0019097	TP53INP2
100511370	1649.510322	323.5339706	2.35	7.27E-06	0.0019097	HSH2D
100739671	33.3272125	195.4511454	-2.552	7.49E-06	0.0019473	ESM1
106505256	9.73146266	182.8252913	-4.2317	7.73E-06	0.001988	LOC106505256
100627807	6065.433252	952.1357064	2.6714	8.09E-06	0.0020426	RBM3
100127358	9473.133327	1935.817034	2.2909	8.61E-06	0.0021393	IFITM1
100523325	8.389476546	208.9731832	-4.6386	8.67E-06	0.0021393	ASB4
100737774	6.484872216	71.39080687	-3.4606	1.11E-05	0.0026914	LRRC17
654317	65.81102037	668.0629696	-3.3436	1.11E-05	0.0026914	NR1I3
110256498	92.6564798	12.96652134	2.8371	1.14E-05	0.0027408	CBS
100156756	14.94665065	165.6888275	-3.4706	1.24E-05	0.0029128	ACVR1C
100157138	3232.912053	370.3044663	3.1261	1.25E-05	0.0029128	PNP
100519115	4.725979052	43.46979927	-3.2013	1.28E-05	0.0029254	ADAMTS16
102161637	7.207162494	177.8711782	-4.6253	1.27E-05	0.0029254	LOC102161637
102164363	2.800451896	35.15656681	-3.6501	1.80E-05	0.0040732	LOC102164363
100738302	439.0498	96.54872451	2.1851	1.91E-05	0.0042847	CEACAM19

100523389	359.2645303	1688.024985	-2.2322	2.03E-05	0.0045111	SLC37A2
397013	56.57283153	310.1053672	-2.4546	2.06E-05	0.0045352	PPARGC1A
100515408	68.6391872	356.8997324	-2.3784	2.11E-05	0.0046004	SHPK
100158214	209.7059571	1008.345277	-2.2655	2.45E-05	0.005313	FLRT3
100522832	7.176178137	54.81016978	-2.9332	2.62E-05	0.0056178	GPR156
100512564	579.2349771	125.4192832	2.2074	2.75E-05	0.0058324	FAM117B
100623615	22.05380402	1.062929658	4.3749	2.76E-05	0.0058324	LY6D
100514979	23.98471332	194.3124127	-3.0182	2.93E-05	0.0061213	LOC100514979
100141405	6044.156959	26182.02079	-2.115	3.13E-05	0.0064922	CTSZ
100511895	621.0209614	2777.401892	-2.161	3.34E-05	0.0068096	SLC25A15
100512800	25.44146335	134.1593691	-2.3987	3.37E-05	0.0068096	SCN4B
396900	476.796436	101.7173123	2.2288	3.32E-05	0.0068096	AMCF-II
100516107	3.096039296	32.41574316	-3.3882	3.44E-05	0.0069095	LRRC3C
100516755	691.5906154	150.3544636	2.2016	3.47E-05	0.0069167	ETV4
100153917	64.86025854	426.8400739	-2.7183	3.52E-05	0.0069272	SLITRK6
100522112	5696.55909	1232.839977	2.2081	3.54E-05	0.0069272	GCNT3
100521587	45.12202169	5.221298311	3.1114	3.77E-05	0.0073318	ECEL1
100515264	358.4501221	1584.003638	-2.1437	4.04E-05	0.0077267	CAMK2N1
110256469	17.94667455	0.718039279	4.6435	4.01E-05	0.0077267	LOC110256469
100511886	735.5754444	5.540820063	7.0526	4.39E-05	0.0083279	FGFBP1
100048965	730.8634264	3079.732671	-2.0751	4.79E-05	0.008958	PGRMC2
100153250	420.00645	3565.119585	-3.0855	4.84E-05	0.0089677	GPD1
110255503	8.806904054	60.47101287	-2.7795	4.90E-05	0.0089677	LOC110255503
110259489	15.6332355	88.56731144	-2.5022	4.87E-05	0.0089677	TIFAB
100623751	82.66254851	397.0831191	-2.2641	5.20E-05	0.0093738	RPS6KA5
397166	11.39774721	71.37140408	-2.6466	5.20E-05	0.0093738	MS4A2
100192320	11.78894389	847.8293013	-6.1683	5.52E-05	0.0098341	BCO1
399532	1270.938292	292.104155	2.1213	5.54E-05	0.0098341	FABP3
100154506	2201.110364	9419.203557	-2.0974	5.65E-05	0.0099617	ENTPD5
403215	137.4240362	763.4250569	-2.4739	6.85E-05	0.011989	CYP2C49
100524750	605.8267097	2402.549581	-1.9876	7.16E-05	0.012442	CYP2J34
100152368	282.0109906	1187.263877	-2.0738	7.33E-05	0.012659	EGLN3
100187577	265.9503026	59.97532994	2.1487	7.62E-05	0.012888	LRP8
100627912	560.6016393	2570.24453	-2.1969	7.57E-05	0.012888	GDPD5

397200	750.135762	3138.344518	-2.0648	7.62E-05	0.012888	SLC28A1
102165025	5.25065098	44.09174326	-3.0699	7.75E-05	0.013013	LOC102165025
100623590	73.9878581	13.73264075	2.4297	8.09E-05	0.013368	PKP1
110257555	7.575735922	61.59810511	-3.0234	8.03E-05	0.013368	LOC110257555
100514726	572.8322276	3707.548527	-2.6943	8.38E-05	0.01361	ENPP7
100521274	849.6270036	3665.863621	-2.1093	8.33E-05	0.01361	FAM131C
100152827	7.33097467	102.4765852	-3.8051	8.83E-05	0.014147	FCER1A
497235	242.9409619	1090.978547	-2.1669	8.79E-05	0.014147	SLC5A10
100524951	23.56593908	1.873750439	3.6527	9.09E-05	0.01447	ACOD1
100625050	1182.670272	279.6946268	2.0801	9.14E-05	0.01447	EPSTI1
100522244	25.81875615	0.197669717	7.0292	9.24E-05	0.014491	SCNN1G
100622812	351.7361089	84.86818193	2.0512	9.28E-05	0.014491	DUSP2
397687	616.8098004	4116.087607	-2.7384	9.38E-05	0.014567	CYP2D25
100151780	155.956284	1078.962561	-2.7904	9.68E-05	0.014757	KCNJ13
399542	15.19405889	0.241943648	5.9727	9.62E-05	0.014757	SLC5A5
100144410	297.3597858	1186.873626	-1.9969	9.77E-05	0.014788	TPPP
100157026	480.2612192	112.0736648	2.0994	9.96E-05	0.014949	MTFR2
102159476	154.1478021	1001.593329	-2.6999	9.99E-05	0.014949	LOC102159476
102165634	472.8300002	1945.280686	-2.0406	0.00010075	0.014982	LOC102165634
106509607	29.0095569	135.7216352	-2.2261	0.00010151	0.015005	LOC106509607
100144591	2094.979824	14887.69734	-2.8291	0.00010217	0.015013	CYBRD1
100515143	20.08467835	147.5994045	-2.8775	0.00010371	0.015138	RNF207
100626343	1161.497253	5574.009335	-2.2627	0.00010425	0.015138	ABCD3
106508195	0.264757499	14.2413282	-5.7493	0.00010502	0.01516	TAS2R40
100737517	3347.802551	14292.34056	-2.094	0.00010645	0.015278	SLC40A1
100515955	47.58790973	226.7998842	-2.2528	0.00010845	0.015386	PTPRU
100627632	22.06275973	111.0958561	-2.3321	0.00010824	0.015386	ZKSCAN7
100519539	152.6369392	832.090423	-2.4466	0.00011026	0.015553	KLHL3
110259946	2.967481256	31.13344386	-3.3912	0.00011129	0.015609	LOC110259946
100294675	380.5537124	5362.424784	-3.8167	0.00011702	0.01632	DGAT2
100621352	1059.07813	76.98765812	3.782	0.00011812	0.016381	LOC100621352
110260940	7.626111177	49.97213525	-2.7121	0.00011907	0.01642	LOC110260940
100739218	6757.85641	1690.290061	1.9993	0.00012571	0.017144	LOC100739218
110259210	89.12761609	14.39446505	2.6304	0.0001274	0.01728	MUC5AC

100158162	394.5254686	96.76357109	2.0276	0.00013172	0.017685	ATP10A
100625759	11.16209921	63.79112034	-2.5147	0.00013221	0.017685	SMIM1
110259705	28.10216831	2410.519864	-6.4225	0.00013254	0.017685	TH
106506279	67.39686297	653.8894402	-3.2783	0.00013592	0.018039	LOC106506279
100512686	504.0663441	81.40127355	2.6305	0.00013889	0.018281	IRS1
100515902	866.9411348	112.2186208	2.9496	0.00014188	0.018281	LOC100515902
100622375	594.3928187	2290.161801	-1.946	0.00014196	0.018281	SHANK3
110255508	104.6192004	21.27835514	2.2977	0.00013961	0.018281	CER1
110260216	221.0892283	23.61761646	3.2267	0.00014219	0.018281	LOC110260216
100155467	1562.213023	391.7909536	1.9954	0.00014728	0.018837	IFIT2
100515422	93.70943933	374.949557	-2.0004	0.00015291	0.019389	MFSD4B
100515639	142.0268003	33.23348577	2.0955	0.00015316	0.019389	PCDH19
100037938	361.5500544	89.84802012	2.0086	0.00015488	0.019506	CD14
403324	2155.222011	8684.428581	-2.0106	0.00016234	0.020342	CYP3A29
397645	16992.6644	4516.589052	1.9116	0.0001723	0.021373	SAT1
110256586	13.89285143	82.44506693	-2.5691	0.00017718	0.021759	LOC110256586
100514482	10023.63523	2698.358014	1.8933	0.00018375	0.022344	AGR2
397182	33.75899299	146.130702	-2.1139	0.0001832	0.022344	IGFBP5
100518544	9569.15744	2532.268539	1.918	0.00018682	0.022539	IFITM3
397134	1685.372701	6100.035918	-1.8558	0.00018718	0.022539	DAO
100037966	138.5566187	543.1278249	-1.9708	0.00018835	0.02257	SOCS2
100520832	81845.56028	9207.749961	3.152	0.00019202	0.022899	LOC100520832
396645	4300.068263	1117.446065	1.9442	0.00019457	0.023091	IRF8
100622336	259.3471014	32.84139438	2.9813	0.00019629	0.023184	CSF3R
102167936	4.030427747	39.00214983	-3.2745	0.00020068	0.023477	LOC102167936
595111	4783.933998	1310.453413	1.8681	0.00020055	0.023477	KLF4
100625807	3419.223635	15588.51731	-2.1887	0.00020209	0.023531	LCT
100736742	17.94295728	86.92780491	-2.2764	0.00020825	0.02391	GJA8
397639	1179.435935	10767.65863	-3.1905	0.00020792	0.02391	EPHX1
404698	929.0959798	3613.018015	-1.9593	0.00020743	0.02391	HYAL1
100153504	276.0777243	56.61329387	2.2859	0.00021285	0.024242	F13A1
100738133	17.97843869	85.10074069	-2.2429	0.00021311	0.024242	LOC100738133
100316849	7210.921095	1855.727433	1.9582	0.00021795	0.024679	ZFP36
100510947	15.34332977	77.7839019	-2.3419	0.00023156	0.025982	PTGDR2

768107	114.2785482	455.4921463	-1.9949	0.00023104	0.025982	RGN
100158055	456.1780182	1663.182452	-1.8663	0.00023628	0.026392	OLFML3
100514091	22.80209036	108.0243122	-2.2441	0.0002442	0.027153	OTOG
100233171	866.9551194	3108.551561	-1.8422	0.00025102	0.027663	AGPAT3
100157065	170.4170669	3277.895143	-4.2656	0.00025674	0.028168	ASAH2
100519179	12.51371594	115.9679587	-3.2121	0.00025869	0.028256	USP51
100155159	68.24241834	468.0860862	-2.778	0.00026249	0.028419	PYROXD2
102163819	1180.492775	6350.825045	-2.4276	0.00026226	0.028419	MS4A10
100157844	3815.951828	1033.049484	1.8851	0.00026681	0.028636	PIM1
100519488	20.40262486	397.0625873	-4.2825	0.0002665	0.028636	MOV10L1
100152428	1444.57619	201.2347481	2.8437	0.00027288	0.02891	LOC100152428
100156914	394.9367636	1365.212127	-1.7894	0.00027592	0.029108	SLC24A4
100514743	381.5275952	1502.293605	-1.9773	0.00028234	0.029532	WIPF3
110256820	181.9357766	37.35058958	2.2842	0.00028133	0.029532	LOC110256820
100514493	966.4977771	3416.808393	-1.8218	0.00029529	0.030627	ATP1B3
102159800	216.1804276	788.5414963	-1.867	0.00029411	0.030627	MVB12B
100523320	1710.728287	406.688303	2.0726	0.00030122	0.031112	SOWAHB
102164898	1229.962097	4416.184739	-1.8442	0.00031306	0.0322	SEC16B
100519529	882.1852765	4065.975569	-2.2044	0.00031863	0.032636	ABCD1
100511161	245.9354104	63.47788325	1.954	0.00033121	0.033646	PLA2G3
100156455	842.8232605	237.4107108	1.8278	0.00034782	0.035045	TC2N
100623476	62.74123826	5.437441252	3.5284	0.00034664	0.035045	HTR6
397376	480.2198741	2261.369685	-2.2354	0.00035096	0.035218	SLC5A4
100622126	2.29140883	23.6761392	-3.3691	0.00035845	0.035823	CX3CR1
100126286	2071.887616	592.6216575	1.8058	0.00036308	0.036033	KRT18
100623809	1340.591308	4734.773665	-1.8204	0.00036347	0.036033	TMC5
100522261	4907.787562	1358.929121	1.8526	0.00037651	0.037177	PGM2
100738062	19.34275318	371.5610028	-4.2637	0.00038127	0.037444	ACOT12
110260714	34.56396482	140.7341254	-2.0256	0.00038226	0.037444	ETFBKMT
396998	13.46511072	93.97928415	-2.8031	0.0003856	0.037623	HPX
396764	200.3987486	31.82829953	2.6545	0.00038791	0.037699	SLC11A1
100514568	34.98548326	141.1495026	-2.0124	0.00039014	0.037767	TMEM38A
397568	2327.273261	8132.841789	-1.8051	0.00039238	0.037835	CAT
100624487	20.80699628	90.22216106	-2.1164	0.00039416	0.037859	SLC2A10

100624671	84.26914401	325.8855905	-1.9513	0.00040769	0.039007	CMTM8
100622156	2538.883969	550.0559698	2.2065	0.00041149	0.039068	TNFAIP3
110260202	1877.043081	1.417553186	10.371	0.00041043	0.039068	ARC
100624791	688.3092863	2342.248125	-1.7668	0.00041325	0.039085	PLXNA2
100511552	15.24435691	0.889391959	4.0993	0.00042669	0.040201	CIB3
110256050	73.16987362	273.277125	-1.901	0.00043806	0.041116	MMP28
100155871	6.387619763	387.7366804	-5.9237	0.00044253	0.041222	SLC16A9
100622227	73.81862221	867.3584972	-3.5546	0.00044204	0.041222	LOC100622227
100520717	40.51546745	7.1731357	2.4978	0.00044836	0.041452	YPEL4
100522981	365.0265404	1291.584317	-1.8231	0.000448	0.041452	HOOK2
100628009	190.7510925	655.2142124	-1.7803	0.00046066	0.042117	XYLB
396934	417.6566396	1511.845878	-1.8559	0.00045956	0.042117	RENBP
397018	842.5857094	2881.928565	-1.7741	0.00045773	0.042117	SCARB1
100738064	957.1500616	0	Inf	0.00046926	0.042745	PLA2G2A
100522516	616.7283834	4196.422871	-2.7665	0.00047664	0.043099	GLYCTK
110260118	304.0286983	82.21296243	1.8868	0.00047538	0.043099	SOWAHC
100522021	305.4248124	1087.50218	-1.8321	0.00048053	0.043237	LRRC66
100739719	44.97617777	322.2472227	-2.8409	0.00048167	0.043237	LOC100739719
397508	104.9002461	13.36180689	2.9728	0.00049262	0.044059	SELE
100515339	47136.17971	9946.57151	2.2446	0.00050216	0.04459	KRT19
100624628	329.0548392	62.18236069	2.4038	0.00050146	0.04459	LOC100624628
100141314	1785.589127	10720.37123	-2.5859	0.000514	0.044785	RARRES2
100516761	151.8693748	536.6607688	-1.8212	0.00050996	0.044785	SLC35A5
102158002	60.65630294	235.5545784	-1.9573	0.00050797	0.044785	LOC102158002
102159603	31.62776592	4.997447955	2.6619	0.00051224	0.044785	LOC102159603
110255963	26.0389622	2.537107024	3.3594	0.00051009	0.044785	LOC110255963
780409	179.2426363	21.10899429	3.086	0.00051525	0.044785	CCL26
100522330	193.0975107	1557.023394	-3.0114	0.00052823	0.045752	SLC7A8
100627227	1646.589954	456.361517	1.8512	0.00053846	0.046476	NPM3
100524265	340.1559041	29.36426244	3.5341	0.00054853	0.04718	CXCL13
100524940	1105.632176	3630.525197	-1.7153	0.00055352	0.047444	LOC100524940
397195	2360.798695	299.6819444	2.9778	0.00056408	0.048181	ADM
448846	891.1766531	3105.707502	-1.8011	0.00056823	0.048368	EHHADH
Novel00365	37.60012025	1.335180448	4.8156	1.03E-07	6.07E-05	-//-

Novel00382	0.264757499	81.96500331	-8.2742	1.18E-07	6.79E-05	-//-
Novel00537	4.632167406	63.42110771	-3.7752	1.27E-07	7.14E-05	--
Novel00251	32.31354463	0.463745383	6.1227	1.13E-08	8.96E-06	-//-
Novel00202	106.4137234	6.424173296	4.05	3.03E-08	2.02E-05	-//-
Novel00495	19.49959134	191.7022351	-3.2974	5.66E-08	3.50E-05	
Novel00078	52.24573973	8.139832967	2.6822	0.00027094	0.028828	-//-
Novel00533	23.99244273	0.865259941	4.7933	0.00027043	0.028828	-//-
Novel00482	0.529514999	24.08282943	-5.5072	0.00032129	0.032773	-//-
Novel00256	4.85765667	35.63236038	-2.8749	0.0002484	0.027497	--
Novel00425	0.264757499	13.28355317	-5.6488	0.00014213	0.018281	-//-
Novel00679	5.124610259	38.63407584	-2.9144	0.0001687	0.021032	--
Novel00257	12.67334323	74.41025704	-2.5537	0.00017637	0.021759	-//-
Novel00280	35.39557215	0.197669717	7.4843	0.00012338	0.01692	--
Novel00255	4.739932283	41.44515844	-3.1283	8.12E-05	0.013368	-//-
Novel00441	13.53391937	77.5882313	-2.5193	9.64E-05	0.014757	-//-
Novel00496	7.243275604	54.89840706	-2.9221	4.59E-05	0.0086434	--
Novel00615	24.79301937	0.637283082	5.2819	8.11E-06	0.0020426	-//-
Novel00535	39.43096643	2.305140059	4.0964	1.18E-05	0.002801	--
Novel00217	17.8130773	0	Inf	9.33E-07	0.00039703	--
Novel00584	10.14141087	149.0529914	-3.8775	1.41E-06	0.00051999	-//-

Table S5. List of genes in different gene clusters.

Gene clusters	Gene ID	Name	Log2 Fold Change	P value	Adjusted P value
Cluster 1	100738064	PLA2G2A	--	4.69E-04	0.042745
	397207	HAMP	--	3.00E-06	0.0010156
	Novel00217	--	--	9.33E-07	0.00039703
Cluster 2	100157662	HOXD1	7.4242	3.77E-06	0.0011702
	100511886	FGFBP1	7.0526	4.39E-05	0.0083279
	100518950	FGF19	7.7152	7.64E-07	0.00033427
	100522244	SCNN1G	7.0292	9.24E-05	0.014491
	110260202	ARC	10.371	4.10E-04	0.039068
	399542	SLC5A5	5.9727	9.62E-05	0.014757
	414904	CXCL2	5.9852	2.58E-07	0.00012984
	Novel00251	--	6.1227	1.13E-08	8.96E-06
	Novel00280	--	7.4843	1.23E-04	0.01692
Cluster 3	595119	OASL	3.6703	3.61E-11	8.10E-08
	733625	SFN	4.4655	4.33E-09	4.65E-06
	100156489	TGM1	4.3808	4.33E-10	5.63E-07
	100157318	APOD	4.1034	2.40E-09	2.69E-06
	100510974	COL28A1	3.6319	2.91E-10	4.23E-07
	100511552	CIB3	4.0993	4.27E-04	0.040201
	100512452	GALNT12	3.4786	8.69E-08	5.23E-05
	100515798	ARL14	3.454	7.87E-10	9.71E-07
	100521889	ADGRE1	3.8241	2.11E-07	0.00010851
	100523844	PIK3C2G	5.4259	2.06E-13	1.02E-09
	100524265	CXCL13	3.5341	5.49E-04	0.04718
	100524951	ACOD1	3.6527	9.09E-05	0.01447
	100525396	LOC100525396	3.7065	9.37E-11	1.78E-07
	100621352	LOC100621352	3.782	1.18E-04	0.016381
	100623476	HTR6	3.5284	3.47E-04	0.035045
	100623615	LY6D	4.3749	2.76E-05	0.0058324
	100737113	LOC100737113	4.3836	4.39E-08	2.85E-05
	100738704	TNIP3	4.6448	1.93E-08	1.32E-05

	106505337	LOC106505337	4.089	1.58E-08	1.15E-05
	110255963	LOC110255963	3.3594	5.10E-04	0.044785
	110256469	LOC110256469	4.6435	4.01E-05	0.0077267
	Novel00202	--	4.05	3.03E-08	2.02E-05
	Novel00365	--	4.8156	1.03E-07	6.07E-05
	Novel00533	--	4.7933	2.70E-04	0.028828
	Novel00535	--	4.0964	1.18E-05	0.002801
	Novel00615	--	5.2819	8.11E-06	0.0020426
Cluster 4	396934	RENBP	-1.8559	4.60E-04	0.042117
	396998	HPX	-2.8031	3.86E-04	0.037623
	397013	PPARGC1A	-2.4546	2.06E-05	0.0045352
	397018	SCARB1	-1.7741	4.58E-04	0.042117
	397134	DAO	-1.8558	1.87E-04	0.022539
	397166	MS4A2	-2.6466	5.20E-05	0.0093738
	397182	IGFBP5	-2.1139	1.83E-04	0.022344
	397200	SLC28A1	-2.0648	7.62E-05	0.012888
	397358	SLC9A3	-2.3939	3.72E-06	0.0011702
	397373	MYO7A	-3.1713	6.32E-09	6.24E-06
	397376	SLC5A4	-2.2354	3.51E-04	0.035218
	397568	CAT	-1.8051	3.92E-04	0.037835
	397639	EPHX1	-3.1905	2.08E-04	0.02391
	397687	CYP2D25	-2.7384	9.38E-05	0.014567
	397691	APOA1	-3.2846	1.85E-08	1.31E-05
	403215	CYP2C49	-2.4739	6.85E-05	0.011989
	403324	CYP3A29	-2.0106	1.62E-04	0.020342
	404698	HYAL1	-1.9593	2.07E-04	0.02391
	448846	EHHADH	-1.8011	5.68E-04	0.048368
	497235	SLC5A10	-2.1669	8.79E-05	0.014147
	654317	NR1I3	-3.3436	1.11E-05	0.0026914
	692188	APOM	-3.5642	7.55E-09	6.90E-06
	768107	RGN	-1.9949	2.31E-04	0.025982
	100037966	SOCS2	-1.9708	1.88E-04	0.02257
	100048965	PGRMC2	-2.0751	4.79E-05	0.008958

100125551	SLC2A8	-3.0009	1.37E-08	1.03E-05
100134962	PHYH	-2.7229	3.06E-07	0.00014822
100141314	RARRES2	-2.5859	0.000514	0.044785
100141405	CTSZ	-2.115	3.13E-05	0.0064922
100144410	TPPP	-1.9969	9.77E-05	0.014788
100144591	CYBRD1	-2.8291	1.02E-04	0.015013
100151780	KCNJ13	-2.7904	9.68E-05	0.014757
100151790	PMM1	-2.4907	4.65E-06	0.0013506
100151843	SNTA1	-2.5747	2.19E-06	0.00076002
100152247	TP53INP2	-2.3722	7.25E-06	0.0019097
100152368	EGLN3	-2.0738	7.33E-05	0.012659
100152827	FCER1A	-3.8051	8.83E-05	0.014147
100153155	XCL1	-2.9181	3.81E-06	0.0011702
100153250	GPD1	-3.0855	4.84E-05	0.0089677
100153752	CLDN10	-3.9658	1.03E-08	8.50E-06
100153917	SLITRK6	-2.7183	3.52E-05	0.0069272
100154506	ENTPD5	-2.0974	5.65E-05	0.0099617
100155159	PYROXD2	-2.778	2.62E-04	0.028419
100155341	NEIL1	-2.528	3.38E-06	0.001129
100156404	PGF	-3.6266	5.82E-07	0.00026583
100156419	HOMER2	-3.3979	2.11E-10	3.26E-07
100156756	ACVR1C	-3.4706	1.24E-05	0.0029128
100156914	SLC24A4	-1.7894	2.76E-04	0.029108
100157774	SLC25A27	-2.3449	5.55E-06	0.001557
100158055	OLFML3	-1.8663	2.36E-04	0.026392
100158214	FLRT3	-2.2655	2.45E-05	0.005313
100158243	SLC16A3	-2.4931	1.26E-06	0.00048441
100233171	AGPAT3	-1.8422	2.51E-04	0.027663
100294675	DGAT2	-3.8167	1.17E-04	0.01632
100510947	PTGDR2	-2.3419	2.32E-04	0.025982
100511895	SLC25A15	-2.161	3.34E-05	0.0068096
100512737	SLC13A2	-2.7472	1.11E-06	0.00044696
100512800	SCN4B	-2.3987	3.37E-05	0.0068096

100513308	TM4SF5	-2.7003	5.22E-06	0.0014994
100514091	OTOG	-2.2441	2.44E-04	0.027153
100514218	GJC2	-3.0439	1.16E-06	0.00045493
100514493	ATP1B3	-1.8218	2.95E-04	0.030627
100514568	TMEM38A	-2.0124	3.90E-04	0.037767
100514726	ENPP7	-2.6943	8.38E-05	0.01361
100514743	WIPF3	-1.9773	2.82E-04	0.029532
100514979	LOC100514979	-3.0182	2.93E-05	0.0061213
100515143	RNF207	-2.8775	1.04E-04	0.015138
100515264	CAMK2N1	-2.1437	4.04E-05	0.0077267
100515408	SHPK	-2.3784	2.11E-05	0.0046004
100515422	MFSD4B	-2.0004	1.53E-04	0.019389
100515460	USP27X	-2.7546	7.09E-06	0.0019025
100515955	PTPRU	-2.2528	1.08E-04	0.015386
100516107	LRRC3C	-3.3882	3.44E-05	0.0069095
100516309	GDPD2	-3.7889	3.12E-10	4.27E-07
100516363	NAT8	-3.2044	1.40E-06	0.00051999
100516578	CCDC80	-2.5001	2.09E-06	0.00073866
100516761	SLC35A5	-1.8212	5.10E-04	0.044785
100517307	SLC6A4	-3.4833	2.07E-09	2.43E-06
100519115	ADAMTS16	-3.2013	1.28E-05	0.0029254
100519179	USP51	-3.2121	2.59E-04	0.028256
100519529	ABCD1	-2.2044	3.19E-04	0.032636
100519539	KLHL3	-2.4466	1.10E-04	0.015553
100520068	PCDH12	-2.8675	4.84E-07	0.00022522
100521274	FAM131C	-2.1093	8.33E-05	0.01361
100521912	WNT11	-2.6049	7.72E-07	0.00033427
100522021	LRRC66	-1.8321	4.81E-04	0.043237
100522330	SLC7A8	-3.0114	5.28E-04	0.045752
100522516	GLYCTK	-2.7665	4.77E-04	0.043099
100522832	GPR156	-2.9332	2.62E-05	0.0056178
100522927	CGREF1	-2.4998	3.55E-06	0.0011539
100522981	HOOK2	-1.8231	4.48E-04	0.041452

100523209	COL7A1	-2.329	4.59E-06	0.0013497
100523389	SLC37A2	-2.2322	2.03E-05	0.0045111
100523600	PCP4L1	-3.1018	5.72E-06	0.0015865
100524747	DBP	-2.5924	1.64E-06	0.00058677
100524750	CYP2J34	-1.9876	7.16E-05	0.012442
100524940	LOC100524940	-1.7153	5.54E-04	0.047444
100622126	CX3CR1	-3.3691	3.58E-04	0.035823
100622227	LOC100622227	-3.5546	4.42E-04	0.041222
100622375	SHANK3	-1.946	1.42E-04	0.018281
100623097	SLC30A10	-3.8052	1.62E-10	2.67E-07
100623751	RPS6KA5	-2.2641	5.20E-05	0.0093738
100623809	TMC5	-1.8204	3.63E-04	0.036033
100624476	PRDM16	-2.2939	5.39E-06	0.0015297
100624487	SLC2A10	-2.1164	3.94E-04	0.037859
100624671	CMTM8	-1.9513	4.08E-04	0.039007
100624791	PLXNA2	-1.7668	4.13E-04	0.039085
100625759	SMIM1	-2.5147	1.32E-04	0.017685
100625807	LCT	-2.1887	2.02E-04	0.023531
100625897	LOC100625897	-2.678	1.55E-07	8.52E-05
100626343	ABCD3	-2.2627	1.04E-04	0.015138
100626731	LOC100626731	-3.0813	1.57E-06	0.00057029
100627632	ZKSCAN7	-2.3321	1.08E-04	0.015386
100627912	GDPD5	-2.1969	7.57E-05	0.012888
100628009	XYLB	-1.7803	4.61E-04	0.042117
100628018	SLC46A1	-3.7775	1.31E-10	2.31E-07
100736742	GJA8	-2.2764	2.08E-04	0.02391
100737517	SLC40A1	-2.094	1.06E-04	0.015278
100737774	LRRC17	-3.4606	1.11E-05	0.0026914
100738133	LOC100738133	-2.2429	2.13E-04	0.024242
100738360	PDIA2	-3.8302	9.90E-12	2.44E-08
100739671	ESM1	-2.552	7.49E-06	0.0019473
100739719	LOC100739719	-2.8409	4.82E-04	0.043237
100739839	SPATC1	-3.2378	1.01E-06	0.00041396

102158002	LOC102158002	-1.9573	5.08E-04	0.044785
102159476	LOC102159476	-2.6999	9.99E-05	0.014949
102159800	MVB12B	-1.867	2.94E-04	0.030627
102161066	LOC102161066	-2.8346	1.34E-06	0.00050976
102163819	MS4A10	-2.4276	2.62E-04	0.028419
102164363	LOC102164363	-3.6501	1.80E-05	0.0040732
102164898	SEC16B	-1.8442	3.13E-04	0.0322
102165025	LOC102165025	-3.0699	7.75E-05	0.013013
102165634	LOC102165634	-2.0406	1.01E-04	0.014982
102167936	LOC102167936	-3.2745	2.01E-04	0.023477
106505853	LOC106505853	-2.6251	3.84E-06	0.0011702
106506279	LOC106506279	-3.2783	1.36E-04	0.018039
106509607	LOC106509607	-2.2261	1.02E-04	0.015005
110255503	LOC110255503	-2.7795	4.90E-05	0.0089677
110256050	MMP28	-1.901	4.38E-04	0.041116
110256150	KCNJ12	-3.1026	9.20E-09	8.11E-06
110256586	LOC110256586	-2.5691	1.77E-04	0.021759
110257275	LOC110257275	-3.2599	2.81E-07	0.00013852
110257555	LOC110257555	-3.0234	8.03E-05	0.013368
110259489	TIFAB	-2.5022	4.87E-05	0.0089677
110259946	LOC110259946	-3.3912	1.11E-04	0.015609
110260714	ETFBKMT	-2.0256	3.82E-04	0.037444
110260940	LOC110260940	-2.7121	1.19E-04	0.01642
110262031	LOC110262031	-2.5289	2.79E-06	0.00095486
Novel00255	--	-3.1283	8.12E-05	0.013368
Novel00256	--	-2.8749	2.48E-04	0.027497
Novel00257	--	-2.5537	1.76E-04	0.021759
Novel00441	--	-2.5193	9.64E-05	0.014757
Novel00495	--	-3.2974	5.66E-08	3.50E-05
Novel00496	--	-2.9221	4.59E-05	0.0086434
Novel00537	--	-3.7752	1.27E-07	7.14E-05
Novel00584	--	-3.8775	1.41E-06	0.00051999
Novel00679	--	-2.9144	1.69E-04	0.021032

	396645	IRF8	1.9442	1.95E-04	0.023091
	396764	SLC11A1	2.6545	3.88E-04	0.037699
	396900	AMCF-II	2.2288	3.32E-05	0.0068096
	397195	ADM	2.9778	5.64E-04	0.048181
	397508	SELE	2.9728	4.93E-04	0.044059
	397645	SAT1	1.9116	1.72E-04	0.021373
	399532	FABP3	2.1213	5.54E-05	0.0098341
	494561	HK2	2.6892	9.90E-07	0.00041396
	595111	KLF4	1.8681	2.01E-04	0.023477
	733608	S100A6	2.918	1.25E-08	9.65E-06
	780409	CCL26	3.086	5.15E-04	0.044785
	100037938	CD14	2.0086	1.55E-04	0.019506
	100126286	KRT18	1.8058	3.63E-04	0.036033
	100127358	IFITM1	2.2909	8.61E-06	0.0021393
	100135678	PDE6C	2.7964	1.12E-06	0.00044696
	100145895	ISG15	2.3519	6.58E-06	0.0018039
Cluster 5	100152428	LOC100152428	2.8437	2.73E-04	0.02891
	100153504	F13A1	2.2859	2.13E-04	0.024242
	100155467	IFIT2	1.9954	1.47E-04	0.018837
	100155893	ARG2	2.4526	3.68E-06	0.0011702
	100156455	TC2N	1.8278	3.48E-04	0.035045
	100156922	MAT1A	3.0554	1.96E-07	0.0001032
	100157026	MTFR2	2.0994	9.96E-05	0.014949
	100157138	PNP	3.1261	1.25E-05	0.0029128
	100157844	PIM1	1.8851	2.67E-04	0.028636
	100158162	ATP10A	2.0276	1.32E-04	0.017685
	100187577	LRP8	2.1487	7.62E-05	0.012888
	100312960	ANXA4	2.6891	6.41E-07	0.00028767
	100316849	ZFP36	1.9582	2.18E-04	0.024679
	100511161	PLA2G3	1.954	3.31E-04	0.033646
	100511370	HSH2D	2.35	7.27E-06	0.0019097
	100512564	FAM117B	2.2074	2.75E-05	0.0058324
	100512686	IRS1	2.6305	1.39E-04	0.018281

100514482	AGR2	1.8933	1.84E-04	0.022344
100515339	KRT19	2.2446	5.02E-04	0.04459
100515639	PCDH19	2.0955	1.53E-04	0.019389
100515902	LOC100515902	2.9496	1.42E-04	0.018281
100516755	ETV4	2.2016	3.47E-05	0.0069167
100518544	IFITM3	1.918	1.87E-04	0.022539
100520717	YPEL4	2.4978	4.48E-04	0.041452
100520832	LOC100520832	3.152	1.92E-04	0.022899
100521587	ECEL1	3.1114	3.77E-05	0.0073318
100522112	GCNT3	2.2081	3.54E-05	0.0069272
100522261	PGM2	1.8526	3.77E-04	0.037177
100523320	SOWAHB	2.0726	3.01E-04	0.031112
100622156	TNFAIP3	2.2065	4.11E-04	0.039068
100622336	CSF3R	2.9813	1.96E-04	0.023184
100622812	DUSP2	2.0512	9.28E-05	0.014491
100623590	PKP1	2.4297	8.09E-05	0.013368
100624628	LOC100624628	2.4038	5.01E-04	0.04459
100625050	EPSTI1	2.0801	9.14E-05	0.01447
100625277	UPP1	2.6795	3.90E-07	0.00018496
100627227	NPM3	1.8512	5.38E-04	0.046476
100627807	RBM3	2.6714	8.09E-06	0.0020426
100738302	CEACAM19	2.1851	1.91E-05	0.0042847
100739218	LOC100739218	1.9993	1.26E-04	0.017144
102159603	LOC102159603	2.6619	5.12E-04	0.044785
106505478	LOC106505478	3.1482	1.00E-08	8.50E-06
110255508	CER1	2.2977	1.40E-04	0.018281
110256043	LOC110256043	2.4585	4.46E-06	0.001327
110256498	CBS	2.8371	1.14E-05	0.0027408
110256820	LOC110256820	2.2842	2.81E-04	0.029532
110259210	MUC5AC	2.6304	1.27E-04	0.01728
110260118	SOWAHC	1.8868	4.75E-04	0.043099
110260216	LOC110260216	3.2267	1.42E-04	0.018281
Novel00078	--	2.6822	2.71E-04	0.028828

	397677	FOLH1B	-5.6844	9.18E-11	1.78E-07
	403104	CYP2B22	-8.8192	2.79E-15	3.45E-11
	406187	APOC3	-4.4897	7.14E-15	5.87E-11
	414380	DIO1	-6.0211	1.10E-19	2.71E-15
	100126844	GATM	-4.3639	4.99E-08	3.16E-05
	100144620	AQP10	-4.197	3.55E-06	0.0011539
	100155204	ZBBX	-4.5526	2.25E-12	6.16E-09
	100155871	SLC16A9	-5.9237	4.43E-04	0.041222
	100157065	ASAH2	-4.2656	2.57E-04	0.028168
	100157319	SDR16C5	-4.9255	2.09E-12	6.16E-09
	100171400	TRPV6	-5.004	3.99E-06	0.0012014
	100192320	BCO1	-6.1683	5.52E-05	0.0098341
	100515970	OSBP2	-4.2461	5.13E-13	2.11E-09
	100519006	DUSP9	-5.5588	6.85E-06	0.0018568
Cluster 6	100519488	MOV10L1	-4.2825	2.67E-04	0.028636
	100523325	ASB4	-4.6386	8.67E-06	0.0021393
	100620627	LOC100620627	-4.9919	6.64E-09	6.31E-06
	100625192	GCKR	-4.0522	1.73E-07	9.31E-05
	100627655	LOC100627655	-5.9322	4.89E-09	5.03E-06
	100738062	ACOT12	-4.2637	3.81E-04	0.037444
	102161637	LOC102161637	-4.6253	1.27E-05	0.0029254
	106505256	LOC106505256	-4.2317	7.73E-06	0.001988
	106508195	TAS2R40	-5.7493	1.05E-04	0.01516
	110255314	LOC110255314	-4.4512	8.44E-13	2.98E-09
	110259705	TH	-6.4225	1.33E-04	0.017685
	110261001	LOC110261001	-6.4452	3.15E-14	1.94E-10
	Novel00382	--	-8.2742	1.18E-07	6.79E-05
	Novel00425	--	-5.6488	1.42E-04	0.018281
	Novel00482	--	-5.5072	3.21E-04	0.032773

Table S6. Gene Ontology analyses for differential expression genes.

GO accession	Description	Term type	P value	Gene ID
GO:0008009	chemokine activity	Molecular function	1.21E-05	780409,396900,100525396,414904,100524265, 100153155
GO:0042379	chemokine receptor binding	Molecular function	1.21E-05	100524265,396900,100525396,414904,780409, 100153155
GO:0001664	G-protein coupled receptor binding	Molecular function	1.74E-05	100524265,100525396,396900,414904,780409, 100153155,397200
GO:0055114	oxidation-reduction process	Biological process	0.00026513	595119,110259210,100520717,100153504,399542,100157026, 100627632,414380,397687,100144591,448846,100626731, 100152368,100153250,397134,397013,403215,403324,403104,100517307,100625192,397568,100524940,100155159,397166,100738360,100524750,100516578,100515970,110259705
GO:0005335	serotonin:sodium symporter activity	Molecular function	0.00040681	100517307, 102164898
GO:0008504	monoamine transmembrane transporter activity	Molecular function	0.00040681	100517307, 102164898
GO:0015222	serotonin transmembrane transporter activity	Molecular function	0.00040681	100517307, 102164898
GO:1901618	organic hydroxy compound transmembrane transporter activity	Molecular function	0.00040681	100623590,100518544,100522244,110256043,100737113,100152428,100126286,399542,100515339,397358,100737517,100626343,100153917,100515422,102164898,397013,100523389,100624487,100155871,100628018,397376,397691,100620627,100514568,406187,100516578,100623097,100125551,100514493,448846,110256150,497235,100158243,100522330, 100512737,100151780,100156419,100171400,100517307,100515970,100519529,100516761,100624671,100156914
GO:1902578	single-organism localization	Biological process	0.00045122	100623590,100518544,100522244,110256043,100737113,100152428,100126286,399542,100515339, 397358,100737517 ,100626343,100153917,100515422,102164898,397013,100523389,100624487,100155871,100628018,397376,397691,1006206

				27,100514568,406187,100516578,100623097,100125551,100514493,448846,110256150,497235,100158243,100522330,100512737,100151780,100156419,100171400,100517307,100515970,100519529,100516761,100624671,100156914
GO:0044765	single-organism transport	Biological process	0.00049795	399542,100515339,100737113,110256043,100152428,100126286,100518544,100522244,100623590, 100515970,100519529, 100156914,100516761,100624671,100151780,100156419,100517307,100171400,497235,100158243,100512737,100522330,110256150,100514493,448846,100620627,100514568,397376,397691,100125551,100623097,406187,100516578,100155871,100628018,102164898,397013,100624487,100523389,100737517,100626343,397358,100515422
GO:0005125	cytokine activity	Molecular function	0.0006886	414904,396900,100525396,100524265,780409, 100153155
GO:0016491	oxidoreductase activity	Molecular function	0.00085566	399542,100157026,595119,110259210,100153504,100520717,100157319,403104,100625192,397568,100738360,100155159,397166,100524940,100516578,100524750,100625807,110259705,100515970,414380,100627632,448846,397687,100626731,100152368,397134,100153250,403324,100625897,403215
GO:0055085	transmembrane transport	Biological process	0.0015613	399542,100623590,100152428, 100516578,100623097,100125551,100156914,397376,100519529,100620627,100171400, 100628018,100155871,100512737,100522330,100624487,100523389,100158243,497235,100515422,397358,100626343,100737517
GO:0016746	transferase activity, transferring acyl groups	Molecular function	0.0017606	397645,100156489,100153504,100521587, 100233171,106506279,100514493,100516363,100294675

GO:0020037	heme binding	Molecular function	0.0020672	403324,403215,100519529,100524750,100524940,397568,397687,403104
GO:0046906	tetrapyrrole binding	Molecular function	0.0028868	100524940,100524750,100519529,403215,403324,397687,403104,397568
GO:0005126	cytokine receptor binding	Molecular function	0.0028969	100524265,414904,396900,100525396,780409 2 100153155,100156914
GO:0003906	DNA-(apurinic or apyrimidinic site) lyase activity	Molecular function	0.004691	100516761,100155341
GO:0005215	transporter activity	Molecular function	0.004932	100518544,100522244,399542,396764,100152428,100623590,497235,100192320,100522330,100512737,110256150,100519529,100516761,100624671,100151780,100171400,100517307,100233171,102164898,397013,100144620,100624487,100523389,397358,100737517,100626343,397376,100620627,100514568,100516578,100623097,100125551,100628018
GO:0008519	ammonium transmembrane transporter activity	Molecular function	0.0067175	102164898,100517307
GO:0019050	suppression by virus of host apoptotic process	Biological process	0.0068383	100624487,100513308
GO:0033668	negative regulation by symbiont of host apoptotic process	Biological process	0.0068383	100624487,100513308
GO:0052041	negative regulation by symbiont of host programmed cell death	Biological process	0.0068383	100624487,100513308
GO:0052490	negative regulation by organism of programmed cell death in other organism involved in symbiotic interaction	Biological process	0.0068383	100624487,100513308
GO:0019054	modulation by virus of host process	Biological process	0.0076563	110260940,100513308,100624487
GO:0044068	modulation by symbiont of host cellular process	Biological process	0.0076563	100513308,110260940,100624487
GO:0016705	oxidoreductase activity, acting on paired donors, with incorporation or reduction of molecular oxygen	Molecular function	0.0078364	403104,397687,100524750,100524940,403324,403215,110259705

GO:0006858	extracellular transport	Biological process	0.0082705	100515339,100126286
GO:0016855	racemase and epimerase activity, acting on amino acids and derivatives	Molecular function	0.0084302	110259210, 448846
GO:0036361	racemase activity, acting on amino acids and derivatives	Molecular function	0.0084302	110259210, 448846
GO:0003824	catalytic activity	Molecular function	0.00885	100156922,100511370,100522112,100156489,100737113,100135678,100152428,100622812,100523844,397645,100625277,100511161,100623590,100520717,100153504,110259210,595119,733625,494561,399542,100157844,100515798,100126286,100522261,100510974,100738704,100521587,100157138,100157026,100524951,100521889,100622336,396645,100621352,100516309,100519529,100156914,100155159,100519179,100514743,100625192,100233171,100628009,110256050,100519115,106509607,100515955,102161066,100514726,100523209,100515143,100523325,100141405,106506279,448846,100514493,397691,397166,100516578,100625807,100524750,100156756,100157319,397373,100516363,100154506,403324,100625897,100515460,100152247,100294675,100519488,100626343,414380,397687,100515970,100519006,100516761,100151780,397568,100156419,404698,403215,106505256,100153250,397134,100627912,100516107,100626731,100623751,100627632,100151790,110259705,100514568,110260714,100738360,100524940,100144410,100157065,403104,100511895,397013,100152368,100515408,100155341
GO:0019988	charged-tRNA amino acid modification	Biological process	0.0093132	100622812, 100519006
GO:0043399	tRNA A64-2'-O-ribosylphosphate transferase activity	Molecular function	0.0093132	100622812, 100519006

Table S7. Pathway analyses for differentially expressed genes.

Term	Database	ID	P-Value	Gene ID
Linoleic acid metabolism	KEGG PATHWAY	ssc00591	0.000278519	100524940|403324|100511161|100524750|403215
Mineral absorption	KEGG PATHWAY	ssc04978	0.00086121	100144591|396764|100514493|100171400|100737517
Galactose metabolism	KEGG PATHWAY	ssc00052	0.001567163	100625807|494561|100522261|100625897
Retinol metabolism	KEGG PATHWAY	ssc00830	0.002408319	100192320|403324|100157319|403104|403215
Metabolic pathways	KEGG PATHWAY	ssc01100	0.002837742	100514726|768107|100294675|100151790|100522261|100625277|100628009|404698|403324|100511161|100625807|100157065|100522112|110259705|397645|100126844|494561|100524750|100625897|403104|100156922|100522516|100233171|100157138|100523844|100192320|403215|100524940|100155893|100512452|397677|397134
Serotonergic synapse	KEGG PATHWAY	ssc04726	0.002904062	403215|100524940|110259705|397687|100517307|100623476|100524750
Fat digestion and absorption	KEGG PATHWAY	ssc04975	0.002986019	100294675|100511161|397018|397691
Arachidonic acid metabolism	KEGG PATHWAY	ssc00590	0.004192787	100524940|100511161|100524750|403104|403215
Aldosterone-regulated sodium reabsorption	KEGG PATHWAY	ssc04960	0.005527373	100512686|100514493|733625|100522244
Carbohydrate digestion and absorption	KEGG PATHWAY	ssc04973	0.005979542	100625807|494561|100514493|100625897
Vitamin digestion and absorption	KEGG PATHWAY	ssc04977	0.006996254	397677|397018|397691
Chemokine signaling pathway	KEGG PATHWAY	ssc04062	0.008410048	100524265|414904|780409|396900|100153155|100525396|110256043|100622126
Asthma	KEGG PATHWAY	ssc05310	0.00869264	110255503|100152827|397166
Amino sugar and nucleotide sugar metabolism	KEGG PATHWAY	ssc00520	0.009843896	494561|396934|100151790|100522261

Table S8. The full set of significant motif occurrences.

Gene clusters	Gene ID	Symbol	TF	Strand	Start	End	p-value	q-value	Matched Sequence
	100521889	ADGRE1	IRF8	-	852	865	7.20E-07	0.0038	CTGAAACTGAAACA
Cluster 3	100521889	ADGRE1	IRF8	-	858	871	1.79E-05	0.0477	AAAAAACTGAAACT
	100510974	COL28A1	IRF8	-	1979	1992	2.10E-06	0.0106	CTGAAAGTGAAACA
	100524951	ACOD1	IRF8	+	1733	1746	7.71E-06	0.041	CCAAAACTGAAAGC
	100145895	ISG15	IRF8	+	2070	2083	5.91E-07	0.00317	AGGAAACCGAAACT
	100145895	ISG15	IRF8	+	1836	1849	5.94E-06	0.0159	TGGAAAGTGAAAGT
	100145895	ISG15	IRF8	-	2090	2103	1.53E-05	0.0269	CCGAAATCGAAATC
Cluster 5	100145895	ISG15	IRF8	+	2076	2089	2.00E-05	0.0269	CCGAAACTGAAGCC
	110256043	LOC110256043	IRF8	+	58	71	1.49E-06	0.00798	AAGAAACCGAAAGC
	100512686	IRS1	IRF8	+	692	705	4.94E-06	0.0206	CTGAAACTGAAATC
	595111	KLF4	IRF8	+	928	941	9.12E-06	0.049	TAGAAAGCGATACC
	397013	PPARGC1A	IRF8	-	51	64	1.39E-06	0.00747	AGGAAACTGAAACT
	110256586	LOC110256586	IRF8	+	934	947	2.50E-06	0.0127	CTGAAACCGAAATC
	100626731	LOC100626731	IRF8	+	996	1009	2.70E-06	0.0145	TTGAAACCGAAATC
Cluster 4	100519539	KLHL3	IRF8	-	2454	2467	6.25E-06	0.0312	ATGAAACTGATACA
	100517307	SLC6A4	IRF8	+	2282	2295	8.60E-06	0.0462	GAGAAAGCGATACT
	110260714	ETFBKMT	IRF8	+	12	25	8.73E-06	0.0466	ATAAAAGCGAAACT
	100625897	LOC100625897	IRF8	+	1460	1473	9.00E-06	0.0466	CAGATACTGAAACC
Cluster 5	100520717	YPEL4	NFIL3	+	1684	1694	2.25E-06	0.0121	TTATGTAATGT
	397166	MS4A2	NFIL3	+	710	720	2.25E-06	0.011	TTATGTAATGT
	448846	EHHADH	NFIL3	-	1628	1638	3.91E-06	0.021	TTATGTAATAT
Cluster 4	100233171	AGPAT3	NFIL3	+	513	523	3.91E-06	0.021	TTATGTAATAT
	100156419	HOMER2	NFIL3	-	2291	2301	4.66E-06	0.0251	TTACGTAACCT
	100522021	LRRC66	NFIL3	+	2170	2180	5.42E-06	0.0268	TTATGTAATCT
Cluster 6	100625192	GCKR	NFIL3	+	2358	2368	4.36E-06	0.0234	TTATGTAACTT
	100518950	FGF19	KLF4	+	825	835	4.21E-06	0.019	CCACACCCAGG
	399542	SLC5A5	KLF4	+	113	123	4.52E-06	0.022	CCACACCCACG
Cluster 2	110260202	ARC	KLF4	+	950	960	7.62E-06	0.0308	CCACACCCATT
	100518950	FGF19	KLF4	-	1924	1934	1.28E-05	0.028	ACACACCCACT
	110260202	ARC	KLF4	+	1375	1385	1.35E-05	0.0308	ACACACCCAGA
Cluster 3	100623615	LY6D	KLF4	+	1290	1300	2.76E-07	0.00128	CCACACCCTCC
	100521889	ADGRE1	KLF4	+	2240	2250	8.89E-07	0.00474	CCACACCCTCT

	106505337	LOC106505337	KLF4	+	1380	1390	1.90E-06	0.00987	CCACACCCTTG
	733625	SFN	KLF4	-	2046	2056	2.27E-06	0.0114	CCACACCCTTT
	100623615	LY6D	KLF4	-	2193	2203	8.44E-06	0.0195	CCACGCCCTCC
	100523389	SLC37A2	KLF4	+	398	408	1.38E-07	0.00071	CCACACCCTGC
	397166	MS4A2	KLF4	+	2270	2280	1.38E-07	0.000743	CCACACCCTGC
	100738360	PDIA2	KLF4	-	83	93	2.76E-07	0.00124	CCACACCCTCC
	100514493	ATP1B3	KLF4	+	58	68	4.14E-07	0.00213	CCACACCCTGG
	396934	RENBP	KLF4	+	1029	1039	7.21E-07	0.00324	CCACACCCTCG
	106509607	LOC106509607	KLF4	+	2348	2358	7.21E-07	0.00361	CCACACCCTCG
	100516578	CCDC80	KLF4	-	892	902	8.89E-07	0.00478	CCACACCCTCT
	100513308	TM4SF5	KLF4	-	1005	1015	8.89E-07	0.00466	CCACACCCTCT
	100738360	PDIA2	KLF4	+	798	808	2.07E-06	0.00464	ACACACCCTCC
	100294675	DGAT2	KLF4	+	414	424	2.41E-06	0.0115	CCACACCCAGC
	100155159	PYROXD2	KLF4	+	717	727	2.55E-06	0.0121	CCACACCCACC
	102159476	LOC102159476	KLF4	+	2686	2696	2.55E-06	0.0131	CCACACCCACC
	110257275	LOC110257275	KLF4	+	2053	2063	3.13E-06	0.0167	CCACACCCTAT
	100737517	SLC40A1	KLF4	-	1055	1065	3.33E-06	0.0168	CCACACCCTAA
	100514218	GJC2	KLF4	-	569	579	3.50E-06	0.0146	ACACACCCTGG
Cluster 4	100738133	LOC100738133	KLF4	+	1576	1586	3.87E-06	0.0205	ACACACCCTCG
	100154506	ENTPD5	KLF4	-	562	572	4.21E-06	0.0212	CCACACCCAGG
	397687	CYP2D25	KLF4	-	1604	1614	4.21E-06	0.0206	ACACACCCTCT
	100158243	SLC16A3	KLF4	+	423	433	4.21E-06	0.0194	CCACACCCAGG
	397013	PPARGC1A	KLF4	-	10	20	4.52E-06	0.0238	CCACACCCAGT
	397018	SCARB1	KLF4	-	512	522	4.52E-06	0.0221	CCACACCCACG
	100627632	ZKSCAN7	KLF4	-	840	850	4.52E-06	0.0238	CCACACCCACG
	102161066	LOC102161066	KLF4	+	894	904	4.52E-06	0.0217	CCACACCCACG
	448846	EHHADH	KLF4	-	1127	1137	5.64E-06	0.03	CCACACCCAGA
	100515955	PTPRU	KLF4	-	1533	1543	5.81E-06	0.03	CCACACCCACA
	100233171	AGPAT3	KLF4	+	327	337	5.81E-06	0.027	CCACACCCACA
	100151843	SNTA1	KLF4	+	2169	2179	5.81E-06	0.0288	CCACACCCACA
	100514218	GJC2	KLF4	-	1122	1132	5.95E-06	0.0146	GCACACCCTGC
	100516578	CCDC80	KLF4	+	2356	2366	6.08E-06	0.0164	GCACACCCTCC
	100626343	ABCD3	KLF4	+	983	993	6.29E-06	0.0326	ACACACCCTAC
	110262031	LOC110262031	KLF4	+	1548	1558	6.46E-06	0.0301	CCACACCCAAC

	100153250	GPD1	KLF4	-	513	523	7.25E-06	0.0358	CCACACCCATG
	100521912	WNT11	KLF4	-	943	953	7.25E-06	0.0343	CCACACCCATG
	100623809	TMC5	KLF4	-	1366	1376	7.25E-06	0.0379	CCACACCCATG
	100156914	SLC24A4	KLF4	-	1336	1346	7.42E-06	0.0371	ACACACCCACC
	100512737	SLC13A2	KLF4	+	723	733	7.42E-06	0.036	ACACACCCACC
	396934	RENBP	KLF4	+	1316	1326	8.44E-06	0.0176	CCACGCCCTCC
	100519179	USP51	KLF4	-	1692	1702	8.91E-06	0.0479	GCACACCCTCT
	100144591	CYBRD1	KLF4	+	575	585	9.08E-06	0.0347	TCACACCCTGC
	102161066	LOC102161066	KLF4	+	828	838	1.13E-05	0.0217	CAACACCCTGC
	396934	RENBP	KLF4	-	2283	2293	1.18E-05	0.0176	CAACACCCTCC
	100141405	CTSZ	KLF4	+	578	588	1.22E-05	0.0432	ACACACCCAGG
	100144591	CYBRD1	KLF4	-	487	497	1.35E-05	0.0347	ACACACCCAGA
	397687	CYP2D25	KLF4	-	1653	1663	1.41E-05	0.0345	ACACACCCACA
	100738360	PDIA2	KLF4	+	2020	2030	1.42E-05	0.0213	CCACGCCCTCT
	100737517	SLC40A1	KLF4	-	2068	2078	1.46E-05	0.0366	CCACGCCCTTC
	397013	PPARGC1A	KLF4	+	2496	2506	1.59E-05	0.0418	GCACACCCAGC
	100141405	CTSZ	KLF4	-	512	522	1.93E-05	0.0432	CCACACCCCGT
	100512737	SLC13A2	KLF4	+	1194	1204	2.10E-05	0.036	CCACACCCCCA
	100512737	SLC13A2	KLF4	+	1547	1557	2.19E-05	0.036	ACACACCCATG
	100515339	KRT19	KLF4	+	183	193	1.38E-07	0.00068	CCACACCCTGC
	100625050	EPSTI1	KLF4	+	138	148	4.14E-07	0.0021	CCACACCCTGG
	100511161	PLA2G3	KLF4	+	2260	2270	8.89E-07	0.00445	CCACACCCTCT
	100515339	KRT19	KLF4	-	2138	2148	1.73E-06	0.00426	ACACACCCTGC
	100126286	KRT18	KLF4	-	1484	1494	1.90E-06	0.00953	CCACACCCTTG
	733608	S100A6	KLF4	-	2439	2449	2.07E-06	0.005577	ACACACCCTCC
	494561	HK2	KLF4	+	1672	1682	2.07E-06	0.0104	ACACACCCTCC
Cluster 5	733608	S100A6	KLF4	-	438	448	2.41E-06	0.00577	CCACACCCAGC
	100156455	TC2N	KLF4	-	2501	2511	2.41E-06	0.0125	CCACACCCAGC
	100622336	CSF3R	KLF4	-	2142	2152	3.50E-06	0.0178	ACACACCCTGG
	100158162	ATP10A	KLF4	+	223	233	4.21E-06	0.0222	CCACACCCAGG
	733608	S100A6	KLF4	+	734	744	4.73E-06	0.00754	ACACACCCTTC
	100512686	IRS1	KLF4	-	233	243	5.10E-06	0.0235	CCACACCCACT
	100623590	PKP1	KLF4	-	1005	1015	5.95E-06	0.0238	GCACACCCTGC
	396764	SLC11A1	KLF4	+	975	985	7.25E-06	0.0306	CCACACCCATG

	494561	HK2	KLF4	+	1213	1223	7.62E-06	0.0192	CCACACCCATT
	100153504	F13A1	KLF4	-	1931	1941	7.98E-06	0.0391	CCACGCCCTGC
	100515902	LOC100515902	KLF4	-	1941	1951	7.98E-06	0.0419	CCACGCCCTGC
	100511161	PLA2G3	KLF4	-	967	977	9.25E-06	0.0231	GCACACCCTTC
	100623590	PKP1	KLF4	-	820	830	9.59E-06	0.0238	TCACACCCTCC
	110260216	LOC110260216	KLF4	-	193	203	1.01E-05	0.0419	CGACACCCTGC
	396764	SLC11A1	KLF4	-	809	819	1.19E-05	0.0306	CCACACCCCCC
	110259210	MUC5AC	KLF4	+	2039	2049	1.32E-05	0.0423	ACACACCCATC
	110260216	LOC110260216	KLF4	+	52	62	1.59E-05	0.0419	TCACACCCTCT
	100625050	EPSTI1	KLF4	+	2087	2097	1.89E-05	0.047	CCACACCCCGG
	110259210	MUC5AC	KLF4	-	1643	1653	1.89E-05	0.0423	CCACACCCCGG
	406187	APOC3	KLF4	+	1318	1328	1.23E-06	0.00619	CCACACCCTGA
	100192320	BCO1	KLF4	+	1542	1552	1.23E-06	0.00643	CCACACCCTGA
	403104	CYP2B22	KLF4	-	2417	2427	2.07E-06	0.0108	ACACACCCTCC
Cluster 6	100171400	TRPV6	KLF4	+	456	466	5.10E-06	0.0255	CCACACCCACT
	403104	CYP2B22	KLF4	-	1640	1650	7.08E-06	0.0185	ACACACCCAGC
	100519006	DUSP9	KLF4	+	2568	2578	8.91E-06	0.0396	GCACACCCTCT
	110259705	TH	KLF4	+	357	367	1.09E-05	0.0486	CCACACCCAAT

Table S9. List of gene associated with H3K4me3 enrichment.

Chromosome	Peak Start	Peak End	Strand	Log 2 fold enrichment of H3K4me3	Distance	Closest TSS ID	Symbol	Gene Start	Gene End
NC_010456.5	98908619	98908850	+	-1.44	149	100155074	A1CF	98908585	98990362
NC_010456.5	98909294	98909665	+	2.05	894	100155074	A1CF	98908585	98990362
NC_010456.5	98908272	98908561	+	-0.76	-168	100155074	A1CF	98908585	98990362
NC_010451.4	12278683	12278971	+	0.40	300	100737883	AAMDC	12278527	12305217
NC_010450.4	55644792	55645073	-	0.05	588	100627475	AASDH	55604394	55645521
NC_010444.4	77373995	77374283	-	1.46	932	100623892	ABCA7	77357161	77375071
NC_010456.5	60305062	60305350	+	0.78	-4687	100153026	ABCB10	60309893	60346283
NC_010448.4	36464866	36465097	+	-0.95	815	110260969	ABCC11	36464166	36546989
NC_010445.4	112000220	112000526	-	0.78	1742	100525041	ABHD1	111996138	112002115
NC_010455.5	147775286	147775599	-	-0.05	2628	100623142	ABHD10	147760742	147778071
NC_010445.4	11004009	11004297	-	2.46	-364	100525863	ABHD11	11000993	11003789
NC_010443.5	225191222	225191453	-	0.46	1991	100155547	ABHD17B	225149922	225193329
NC_010449.5	49541720	49542008	+	1.78	1166	100522555	ABHD17C	49540698	49599323
NC_010455.5	27006966	27007306	+	-1.27	805	497624	ABHD5	27006331	27053760
NC_010443.5	270762880	270763111	+	0.20	1166	100524544	ABL1	270761829	270906708
NC_010455.5	23019953	23020312	-	2.73	5	100515577	ACAA1	22964844	23020138
NC_010451.4	60807769	60808057	+	0.95	452	100627341	ACAD8	60807461	60828379
NC_010452.4	48921782	48922212	-	0.73	-667	100514676	ACBD5	48883116	48921330
NC_010451.4	11004967	11005255	+	1.78	407	100524160	ACER3	11004704	11167386
NC_010455.5	73774435	73774671	+	0.37	691	100037292	ACKR4	73773862	73777770
NC_010448.4	55539630	55539958	+	1.68	4388	100620403	ACPT	55535406	55541929
NC_010447.5	64038419	64038707	+	0.61	1753	397317	ACRBP	64036810	64047846
NC_010454.4	26714801	26715089	+	3.78	1155	100512859	ACSF2	26713790	26752352
NC_010457.5	124742941	124743331	+	-1.29	1147	100233169	ACSL3	124741989	124816955
NC_010456.5	122776624	122776912	+	1.73	-376	100157521	ACSL5	122777144	122828054
NC_010445.4	4088277	4088593	-	2.46	1948	414396	ACTB	4084275	4090383
NC_010458.4	36471929	36472217	-	3.20	-297	100522578	ACTBL2	36469062	36471776
NC_010443.5	136279644	136279875	+	-0.71	-1359	100152267	ACTC1	136281119	136286551
NC_010455.5	117597564	117597852	+	2.95	-707	100523570	ACTL6A	117598415	117627311
NC_010456.5	113498694	113499145	-	1.27	-126	100156619	ACTR1A	113480196	113498793
NC_010443.5	113288749	113289037	+	4.05	-587	397345	ADAM10	113289480	113407940

NC_010454.4	18597789	18598077	-	1.46	-519	100525073	ADAM11	18575727	18597414
NC_010457.5	47210199	47210492	+	-0.71	2618	397006	ADAM2	47207727	47278315
NC_010456.5	8485417	8485648	+	-1.35	1141	100157954	ADAM7	8484391	8554879
NC_010450.4	1628259	1628490	+	-2.71	1228	100517353	ADD1	1627146	1710653
NC_010450.4	121267435	121267723	+	1.14	785	100513555	ADH5	121266794	121282313
NC_010456.5	10734110	10734645	-	2.46	-999	100144471	ADRA1A	10623391	10733378
NC_010447.5	53688227	53688510	-	-0.22	3118	100522805	AEBP2	53617362	53691487
NC_010443.5	1696237	1696539	-	3.05	2086	100514741	AFDN	1569652	1698474
NC_010450.4	69709853	69710226	+	1.88	3216	100628071	AFM	69706823	69728268
NC_010457.5	39713502	39713877	-	2.24	876	100152203	AGA	39702900	39714566
NC_010444.4	14935372	14935603	+	-1.44	2299	100514151	AGBL2	14933188	14971416
NC_010457.5	128898557	128898986	+	1.10	-34	100514990	AGFG1	128898806	128980782
NC_010444.4	85891238	85891469	+	-0.12	930	100520064	AGGF1	85890423	85941314
NC_010448.4	92082836	92083175	+	-1.12	1241	100499509	AGO3	92081764	92225811
NC_010448.4	91975811	91976099	+	1.46	4632	100499507	AGO4	91971323	92013030
NC_010457.5	37801239	37801642	+	2.68	319	100233172	AGPAT5	37801121	37853472
NC_010446.5	110131636	110131924	-	2.20	1046	100512899	AHCYL1	110092983	110132826
NC_010449.5	100513583	100513992	+	1.31	3191	100626607	AHSA1	100510596	100520353
NC_010449.5	100511256	100511487	+	-1.12	775	100626607	AHSA1	100510596	100520353
NC_010452.4	11468003	11468343	-	2.78	-1794	100518326	AIDA	11430758	11466379
NC_010444.4	5049093	5049402	-	1.61	-362	100511065	AIP	5042883	5048885
NC_010448.4	89316016	89316304	-	-1.44	1880	100620859	AK2	89295166	89318040
NC_010451.4	71895356	71895690	+	2.05	-103	396908	AKAP9	71895626	72054898
NC_010451.4	71896205	71896581	+	0.73	767	396908	AKAP9	71895626	72054898
NC_010443.5	56070390	56070621	-	-0.93	-323	100519991	AKIRIN2	56047188	56070182
NC_010455.5	34334627	34335411	+	3.20	1756	100307125	ALAS1	34333263	34347435
NC_010461.5	47892305	47892593	-	1.46	3592	100518817	ALAS2	47871519	47896041
NC_010451.4	39620051	39620295	-	0.56	-274	100519965	ALG9	39531280	39619899
NC_010450.4	110367640	110368101	-	1.22	130	100514116	ALPK1	110241220	110368001
NC_010443.5	76841420	76841757	+	-1.27	2140	100155925	AMD1	76839448	76863745
NC_010448.4	29362288	29362541	+	0.46	-4397	100516197	AMFR	29366812	29417800
NC_010461.5	92649013	92649301	-	3.46	-2761	100158213	AMOT	92581995	92646396
NC_010454.4	11894721	11895009	-	1.20	616	100626953	AMZ2	11885682	11895481
NC_010456.5	75082438	75082753	+	2.88	-130	100155019	ANAPC16	75082726	75093078

NC_010456.5	31259943	31260700	+	1.56	594	100152087	ANAPC5	31259727	31301153
NC_010456.5	31790304	31790590	-	-1.12	-697	100155743	ANAPC7	31744729	31789750
NC_010451.4	32622746	32622982	-	-1.12	4473	100519911	ANGPTL5	32610041	32627337
NC_010448.4	98813336	98813813	-	0.00	1117	100519490	ANKRD12	98697220	98814692
NC_010456.5	100771466	100771754	-	1.88	802	100152630	ANKRD22	100747955	100772412
NC_010447.5	17249843	17250131	+	1.46	2647	100522737	ANKRD33	17247340	17251099
NW_018084979.1	1265398	1265698	-	2.20	-1318	100739812	ANKRD9	1257830	1264230
NC_010445.4	24849244	24849547	-	-1.12	3420	100525512	ANKS4B	24836446	24852816
NC_010456.5	21315891	21316181	-	2.20	-776	100627945	ANXA10	21249020	21315260
NC_010450.4	102389396	102389684	+	1.88	1707	100521982	ANXA5	102387833	102420868
NC_010448.4	14807290	14807748	-	2.20	2302	100514713	AP1G1	14719949	14809821
NC_010444.4	87141087	87141418	-	0.07	1469	100049670	AP3B1	86875922	87142722
NC_010450.4	32303886	32304174	-	1.78	-1793	100514856	APBB2	31921587	32302237
NC_010444.4	18993773	18994128	-	3.05	284	100522006	API5	18962262	18994235
NC_010447.5	62816032	62816320	-	0.46	4353	100512412	APOBEC1	62811819	62820529
NC_010454.4	37614402	37614690	+	2.05	776	100525889	APPBP2	37613770	37678710
NC_010452.4	33470310	33470598	+	0.88	365	387596	APTX	33470089	33495584
NC_010451.4	12067697	12067928	+	-0.71	2262	100127151	AQP11	12065550	12076339
NC_010455.5	39428443	39428731	-	3.31	1590	595108	ARF4	39410094	39430177
NC_010460.4	20697445	20697747	-	1.20	1039	100270722	ARF5	20695706	20698635
NC_010456.5	89326590	89326821	-	-0.44	4923	102159729	ARHGAP22	89243015	89331629
NC_010451.4	31721863	31722152	+	0.73	1421	100519555	ARHGAP42	31720586	31999730
NC_010449.5	67592850	67593296	-	-0.86	1170	100520849	ARHGAP5	67494237	67594243
NC_010447.5	22761035	22761598	-	2.61	-901	100518732	ARHGAP9	22747346	22760415
NC_010446.5	94054420	94054781	+	2.05	-1843	100145887	ARHGEF2	94056444	94082206
NC_010446.5	94053878	94054186	+	3.05	-2412	100145887	ARHGEF2	94056444	94082206
NC_010450.4	116200031	116200319	-	2.20	-901	100738766	ARHGEF38	116063725	116199274
NC_010453.5	77428936	77429210	+	-0.12	1721	100516509	ARHGEF7	77427352	77522698
NC_010447.5	76635054	76635632	+	1.46	-2042	100625757	ARID2	76637385	76800155
NC_010447.5	82796898	82797280	+	2.05	1348	595113	ARL1	82795741	82812153
NC_010451.4	82226247	82226632	+	-0.76	440	595121	ARL4A	82225999	82228951
NC_010452.4	55700979	55701279	-	-1.12	-740	100627052	ARL5B	55675129	55700389
NC_010452.4	55698655	55698943	-	1.78	1590	100627052	ARL5B	55675129	55700389
NC_010445.4	17109349	17109637	-	2.20	-970	110255213	ARMC5	17100612	17108523

NC_010457.5	120299095	120299569	+	2.20	2210	100153175	ARPC2	120297122	120328795
NC_010457.5	120298285	120298573	+	0.14	1307	100153175	ARPC2	120297122	120328795
NC_010444.4	98441802	98442469	-	1.63	2544	100520233	ARRDC3	98430451	98444680
NC_010443.5	67656038	67656326	-	2.20	898	100625273	ASCC3	67331939	67657080
NC_010443.5	67656581	67656920	-	2.29	329	100625273	ASCC3	67331939	67657080
NC_010444.4	66260982	66261270	-	3.46	9	100520362	ASNA1	66251381	66261135
NC_010445.4	42192020	42192251	-	-0.12	766	100511749	ASPN	42165553	42192902
NC_010456.5	131580110	131580763	-	1.34	-674	100512816	ATE1	131412628	131579762
NC_010447.5	16618038	16618326	+	0.46	-948	102158891	ATF1	16619130	16681391
NC_010457.5	81075953	81076261	-	1.05	441	100513441	ATF2	80984969	81076548
NC_010448.4	54942973	54943218	+	-2.12	-2285	100523675	ATF5	54945381	54950142
NC_010447.5	18837411	18837645	-	-1.71	891	100518609	ATF7	18726329	18838419
NC_010444.4	119964955	119965186	-	-1.71	631	100462745	ATG12	119948443	119965702
NC_010443.5	184747993	184748496	-	1.73	-4492	100155666	ATG14	184701020	184743752
NC_010444.4	8361216	8361456	+	-1.93	-556	100512265	ATL3	8361892	8408919
NC_010456.5	71401595	71401883	-	1.88	740	100624800	ATOH7	71398939	71402479
NC_010450.4	37412322	37412669	+	1.56	4818	100520087	ATP10D	37407677	37531707
NC_010455.5	120728906	120729194	+	1.20	1388	100624673	ATP11B	120727662	120873907
NC_010461.5	114570900	114571131	-	-1.35	4642	100523658	ATP11C	114394436	114575658
NC_010455.5	82681509	82681758	+	-0.44	1191	100514493	ATP1B3	82680442	82727052
NC_010451.4	64335858	64336158	+	2.46	-460	100623420	ATP2B4	64336468	64445669
NC_010448.4	45057578	45058009	-	1.10	2	397552	ATP4A	44984015	45057796
NC_010445.4	6395938	6396351	-	2.63	706	100037979	ATP5J2	6389338	6396851
NC_010443.5	180068746	180069034	+	2.46	-267	100157404	ATP5S	180069157	180081322
NC_010443.5	180069744	180070110	+	3.22	770	100157404	ATP5S	180069157	180081322
NC_010443.5	255151933	255152245	+	3.78	180	100154379	ATP6V1G1	255151909	255159299
NC_010443.5	121700274	121700562	+	1.46	2656	100154720	ATP8B4	121697762	121914633
NC_010455.5	31163896	31164184	+	1.78	4191	100523399	ATRIP	31159849	31178716
NC_010447.5	37978106	37978562	+	-2.58	-378	100524374	ATXN7L3B	37978712	37982432
NC_010456.5	2542969	2543200	-	-0.86	3244	100155020	AUH	2398456	2546329
NC_010456.5	2545300	2545568	-	-1.35	895	100155020	AUH	2398456	2546329
NC_010448.4	63657156	63657470	-	3.78	216	110261341	AURKAIP1	63656120	63657529
NC_010445.4	13561533	13561846	+	-0.12	-3749	100515476	AUTS2	13565439	14794365
NC_010461.5	56263503	56264038	+	2.73	688	100517507	AWAT1	56263082	56273169

NC_010454.4	12342780	12343068	-	2.20	-44	100739604	AXIN2	12311293	12342880
NC_010455.5	100815827	100816115	-	1.20	-4622	397634	B3GALNT1	100772892	100811349
NC_010452.4	609474	609705	+	-1.44	-3874	100525885	B3GALT2	613464	623587
NC_010445.4	79687948	79688236	-	0.88	1586	100524195	B3GNT2	79671393	79689678
NC_010449.5	28683002	28683233	-	-0.35	-2620	100522554	BAG2	28668587	28680497
NC_010445.4	5566250	5566481	-	-0.54	1560	100521439	BAIAP2L1	5472305	5567926
NC_010455.5	34552638	34552926	-	0.88	727	100154798	BAP1	34544570	34553509
NC_010448.4	54221706	54221994	+	3.20	-491	396633	BAX	54222341	54228150
NC_010446.5	105992837	105993136	+	0.33	428	100154006	BCAS2	105992558	106003160
NC_010446.5	106676394	106676770	+	-1.00	3027	100517031	BCL2L15	106673555	106683103
NC_010451.4	39872065	39872559	+	1.56	281	100517547	BCO2	39872031	39916491
NC_010458.4	47801847	47802213	+	-1.41	757	100517687	BDP1	47801273	47909513
NC_010454.4	20042039	20042327	+	2.46	269	733576	BECN1	20041914	20054249
NC_010447.5	42142380	42142700	-	3.68	1127	100511132	BICD1	41897611	42143667
NC_010456.5	6923283	6923635	-	0.14	3491	100153227	BIN3	6874281	6926950
NC_010446.5	81639425	81639713	-	1.46	219	100622972	BLZF1	81597312	81639788
NC_010443.5	183418994	183419742	-	2.18	3520	100113425	BMP4	183415989	183422888
NC_010457.5	105972786	105973420	+	-0.05	1526	100127483	BMPR2	105971577	106132496
NC_010444.4	58684479	58684802	+	1.88	132	100620218	BORCS8	58684508	58696586
NC_010445.4	1908723	1908954	+	-1.12	3190	100623177	BRAT1	1905648	1918229
NC_010449.5	25149363	25149668	+	-1.12	-2791	100141307	BRD2	25152307	25162576
NC_010444.4	140202092	140202380	-	1.88	11	100518182	BRD8	140182195	140202247
NC_010454.4	36498103	36498518	+	1.88	172	100525359	BRIP1	36498138	36639847
NC_010455.5	66018501	66018789	+	1.20	-2403	100155061	BRPF1	66021048	66037446
NC_010455.5	202896279	202896652	-	2.46	79	100738890	BRWD1	202776040	202896545
NC_010444.4	9052787	9053086	+	1.46	205	100144883	BSCL2	9052731	9064890
NC_010456.5	103531115	103531422	+	2.05	521	100156116	BTAF1	103530747	103641082
NC_010456.5	103530581	103531045	+	1.46	66	100156116	BTAF1	103530747	103641082
NC_010449.5	51878015	51878605	+	-0.54	864	100154013	BTBD1	51877446	51925886
NC_010459.5	20846741	20847053	+	-0.61	2114	100156598	BTBD3	20844783	20878217
NC_010448.4	160135299	160135587	-	2.85	763	100736651	BTF3L4	160109492	160136206
NC_010451.4	64035039	64035327	+	1.46	1330	100048932	BTG2	64033853	64037795
NC_010451.4	64036938	64037226	+	1.88	3229	100048932	BTG2	64033853	64037795
NC_010451.4	39284195	39284483	-	1.05	-3012	100514308	BTG4	39253116	39281327

NC_010451.4	85888300	85888598	+	0.88	616	100514864	BZW2	85887833	85947410
NC_010452.4	32429779	32430067	+	0.61	-1819	100522632	C10H9orf24	32431742	32435622
NC_010454.4	50728648	50729117	-	2.78	-114	100521293	C12H17orf100	50644783	50728768
NC_010455.5	182409282	182409513	-	0.65	-335	100736918	C13H21orf91	182374587	182409062
NC_010455.5	182407706	182407938	-	-0.12	1240	100736918	C13H21orf91	182374587	182409062
NC_010455.5	182412017	182412362	-	3.27	-3127	100736918	C13H21orf91	182374587	182409062
NC_010455.5	141073247	141073535	-	2.46	-4655	100511595	C13H3orf30	141064655	141068736
NC_010455.5	141066139	141066458	-	2.78	2437	100511595	C13H3orf30	141064655	141068736
NC_010455.5	84635653	84635941	+	3.27	1081	100517858	C13H3orf58	84634716	84656112
NC_010456.5	13890552	13891089	+	2.78	474	100152195	C14H8orf74	13890346	13916783
NC_010458.4	17848349	17848637	+	2.46	221	100627703	C16H5orf22	17848272	17868151
NC_010458.4	17849616	17849904	+	3.46	1488	100627703	C16H5orf22	17848272	17868151
NC_010459.5	14610759	14611078	+	1.88	-1763	100739819	C17H20orf196	14612682	14706082
NC_010443.5	175147064	175147352	+	2.05	1290	100736797	C1H14orf28	175145918	175156597
NC_010443.5	223157386	223157617	+	1.46	3822	100514445	C1H9orf135	223153679	223315883
NC_010448.4	80584101	80584389	+	2.46	-2035	445461	C1QA	80586280	80589213
NC_010451.4	46321741	46322029	+	1.20	124	100518141	C2CD2L	46321761	46331078
NC_010444.4	136299260	136299732	-	0.05	-371	100511071	C2H5orf15	136286507	136299125
NC_010444.4	137039172	137039403	+	2.14	1316	100627041	C2H5orf24	137037971	137049449
NC_010444.4	108734381	108734848	+	0.73	-4217	100625150	C2H5orf30	108738832	108753016
NC_010449.5	24068237	24068599	+	2.46	399	445467	C4A	24068019	24083060
NC_010451.4	67731133	67731364	+	0.14	140	100520761	C4BPA	67731108	67774369
NC_010446.5	51468437	51468750	+	-0.97	-560	100522371	C4H8orf59	51469154	51479506
NC_010447.5	18515336	18515624	+	0.56	-1467	100516439	C5H12orf10	18516947	18526069
NC_010447.5	79575912	79576200	-	3.46	-58	100623156	C5H12orf45	79562697	79575998
NC_010447.5	28820272	28820503	-	-2.71	-1251	100737998	C5H12orf56	28716366	28819136
NC_010448.4	155569826	155570057	+	-2.44	2945	100512236	C6H1orf168	155566996	155684814
NC_010449.5	76033556	76034067	+	-0.60	61	100626253	C7H14orf93	76033750	76061877
NC_010449.5	58388375	58388682	-	4.27	1588	100154113	C7H15orf39	58380602	58390117
NC_010446.5	98829952	98830247	-	1.98	-1165	100153371	CA14	98821577	98828934
NC_010448.4	1495507	1495947	+	2.46	-60	110260889	CA5A	1495787	1539389
NC_010456.5	49787712	49788193	-	2.05	-239	100157110	CABIN1	49688101	49787713
NC_010444.4	66103178	66103474	-	0.29	-1252	100381266	CALR	66098263	66102074
NC_010444.4	66100862	66101150	-	2.46	1068	100381266	CALR	66098263	66102074

NC_010460.4	19874757	19875045	-	2.78	903	397301	CALU	19841718	19875804
NC_010450.4	108916229	108916617	+	0.61	404	397674	CAMK2D	108916019	109221317
NC_010447.5	31656720	31657008	+	3.78	-644	100514460	CAND1	31657508	31700163
NC_010457.5	139556153	139556441	+	2.20	-1347	724069	CAPN10	139557644	139568764
NC_010455.5	2448362	2449120	+	3.16	-3644	100037936	CAPN7	2452385	2503575
NC_010447.5	43041034	43041322	+	3.46	-1732	100624034	CAPRIN2	43042910	43087644
NC_010446.5	107819474	107819804	-	0.24	2167	100037957	CAPZA1	107769570	107821806
NC_010457.5	106410263	106410551	+	1.68	162	100627358	CARF	106410245	106466716
NC_010457.5	106409897	106410157	+	-1.00	-218	100627358	CARF	106410245	106466716
NC_010444.4	2163642	2163936	-	2.05	-419	100516312	CARS	2119579	2163370
NC_010453.5	77269614	77270089	-	3.05	84	100515851	CARS2	77231866	77269936
NC_010451.4	74276131	74276473	+	0.78	-231	100521674	CASD1	74276533	74347608
NC_010443.5	57833478	57833948	+	2.14	-924	100523759	CASP8AP2	57834637	57864837
NC_010455.5	153481200	153481488	+	1.46	-398	100621690	CBLB	153481742	153700089
NC_010451.4	107455968	107456256	+	1.14	-533	100623482	CBLL1	107456645	107474615
NC_010459.5	56681693	56681988	-	3.05	1420	100141408	CBLN4	56674872	56683261
NC_010455.5	206232104	206232392	-	2.20	-1979	110256498	CBS	206205942	206230269
NC_010447.5	19482752	19483026	-	-1.35	1626	100519426	CBX5	19446083	19484515
NC_010444.4	65252601	65252889	-	3.05	-264	100511068	CC2D1A	65235757	65252481
NC_010443.5	153928947	153929255	-	0.46	3995	106506845	CCDC102B	153687961	153933096
NC_010451.4	102919340	102919626	-	-0.35	275	100737427	CCDC146	102784299	102919758
NC_010451.4	52146489	52146832	+	1.14	350	100525001	CCDC15	52146310	52217679
NC_010457.5	100538854	100539187	+	3.27	-372	100154260	CCDC150	100539393	100634578
NC_010457.5	76005310	76005598	-	1.05	1652	100520768	CCDC173	75980102	76007106
NC_010446.5	124009196	124009626	-	2.46	165	100518781	CCDC18	123837581	124009576
NC_010450.4	102259852	102260240	+	2.01	-300	100415929	CCNA2	102260346	102266274
NC_010443.5	66499228	66499504	-	-2.44	388	100153639	CCNC	66472622	66499754
NC_010447.5	66116478	66116709	-	-0.76	-2018	397162	CCND2	66092483	66114575
NC_010443.5	128268484	128268892	-	1.68	846	106509015	CCNDBP1	128253111	128269534
NC_010450.4	72789190	72789421	+	1.14	-1088	100523979	CCNG2	72790394	72800553
NC_010449.5	120487835	120488066	+	-1.71	810	100157799	CCNK	120487140	120514223
NC_010455.5	97002678	97002966	-	1.68	1579	100626293	CCNL1	96986274	97004401
NC_010457.5	110873208	110873744	+	3.14	-467	100520645	CCNYL1	110873943	110910929
NC_010455.5	29399353	29399641	+	2.78	-3438	414370	CCRL2	29402935	29406541

NC_010447.5	33859256	33859694	+	1.73	961	100157776	CCT2	33858514	33873596
NC_010446.5	93734257	93734612	+	2.46	467	100154317	CCT3	93733967	93753340
NC_010445.4	79924005	79924293	+	2.78	-189	100521551	CCT4	79924338	79941926
NC_010445.4	79925009	79925536	+	-0.80	934	100521551	CCT4	79924338	79941926
NC_010447.5	63514389	63514677	-	3.20	3	100144477	CD163L1	63488678	63514536
NC_010446.5	103977039	103977327	-	0.78	-212	396662	CD2	103963802	103976971
NC_010445.4	17957399	17957806	+	1.20	-104	100621208	CD2BP2	17957707	17961804
NC_010448.4	54444184	54444472	+	2.92	3589	100624694	CD37	54440739	54448193
NC_010451.4	135077713	135077989	-	-0.76	1059	396922	CD46	135025439	135078910
NC_010455.5	151430473	151430857	+	1.92	1490	397042	CD47	151429175	151484867
NC_010443.5	76180489	76180822	+	-0.93	1486	100511111	CDC40	76179169	76234690
NC_010448.4	80036011	80036348	+	1.14	1144	780428	CDC42	80035035	80089243
NC_010452.4	14939922	14940153	-	0.46	950	100511216	CDC42BPA	14649278	14940988
NC_010446.5	98175566	98175854	+	1.29	3773	100156313	CDC42SE1	98171937	98180577
NC_010449.5	39385391	39385622	+	-1.76	1109	100153900	CDC5L	39384397	39427729
NC_010449.5	39384266	39384556	+	0.73	14	100153900	CDC5L	39384397	39427729
NC_010443.5	159923171	159923402	-	-1.12	-360	100522414	CDH20	159818160	159922926
NC_010453.5	4055326	4055637	+	-0.71	1039	100739805	CDK8	4054442	4161501
NC_010443.5	268276675	268277076	+	4.27	-207	100307051	CDK9	268277083	268280493
NC_010445.4	101086131	101086419	+	2.78	-881	100738632	CDKL4	101087156	101138839
NC_010449.5	32352759	32352990	+	-1.12	-1901	100152215	CDKN1A	32354776	32363771
NC_010457.5	44885916	44886213	+	2.68	442	100513762	CDKN2AIP	44885622	44890504
NC_010450.4	134039600	134039888	-	0.46	1031	733582	CDS1	133959250	134040775
NC_010449.5	75924239	75924527	+	1.88	-2269	100157564	CEBPE	75926652	75929949
NC_010446.5	110844777	110845065	-	1.78	433	106510206	CELSR2	110819422	110845354
NC_010450.4	65315029	65315363	-	2.46	592	100737668	CENPC	65237825	65315788
NC_010450.4	117889200	117889675	+	3.88	469	100626792	CENPE	117888968	117965325
NC_010451.4	129011907	129012195	-	0.88	190	100521032	CENPF	128948386	129012241
NC_010451.4	129010559	129010847	-	0.88	1538	100521032	CENPF	128948386	129012241
NC_010458.4	47353387	47353743	+	2.88	246	100736772	CENPH	47353319	47378746
NC_010449.5	43624581	43624889	+	1.68	1	100513550	CENPQ	43624734	43639907
NC_010457.5	45851407	45851695	-	1.20	-92	100515416	CENPU	45818562	45851459
NC_010443.5	122878449	122878856	+	1.46	2188	100152345	CEP152	122876464	122979851
NC_010448.4	42815042	42815333	-	0.68	98	100620768	CEP89	42733675	42815286

NC_010455.5	157387992	157388280	-	1.78	289	100521822	CEP97	157356248	157388425
NC_010446.5	98252530	98252818	+	2.78	1186	100156737	CERS2	98251488	98260504
NC_010448.4	27632511	27632800	+	1.01	4943	100626873	CES3	27627712	27637827
NC_010444.4	97593362	97593747	-	2.27	-234	574052	CETN3	97574003	97593320
NC_010448.4	12226486	12226774	+	3.05	2929	100144464	CFDP1	12223701	12354180
NC_010449.5	86105226	86105568	-	1.73	2489	100156869	CHD2	85980759	86107886
NC_010455.5	35949089	35949377	-	1.05	-980	100151982	CHDH	35910688	35948253
NC_010455.5	168569759	168570076	-	2.46	-343	100516044	CHMP2B	168534950	168569574
NC_010446.5	54894081	54894475	-	0.46	951	100154336	CHMP4C	54860481	54895229
NC_010456.5	7390182	7390470	+	0.88	260	110256869	CHMP7	7390066	7403657
NC_010443.5	139802304	139802592	-	1.20	2010	100520288	CHSY1	139730703	139804458
NC_010449.5	55798207	55798545	-	1.78	290	100157484	CIB1	55794834	55798666
NC_010443.5	25154507	25154825	+	2.20	-986	100156022	CITED2	25155652	25158517
NC_010448.4	170324976	170325326	+	1.46	364	100525468	CITED4	170324787	170326140
NC_010443.5	268642041	268642329	-	2.46	-121	100157558	CIZ1	268618972	268642064
NC_010453.5	15751234	15751492	+	0.24	-354	100515274	CKAP2	15751717	15767068
NC_010453.5	15750609	15750897	+	1.05	-964	100515274	CKAP2	15751717	15767068
NC_010453.5	15752396	15752684	+	2.46	823	100515274	CKAP2	15751717	15767068
NC_010446.5	94819408	94819742	-	2.78	8	100155110	CKS1B	94815769	94819583
NC_010455.5	19509585	19510008	-	2.14	781	100512630	CLASP2	19305430	19510578
NC_010446.5	129652723	129653274	-	1.92	2386	397284	CLCA1	129621259	129655385
NC_010446.5	111081083	111081371	+	2.27	575	100155876	CLCC1	111080652	111101007
NC_010459.5	481865	482153	-	1.88	2677	100156235	CLDN23	483635	484686
NC_010455.5	160396836	160397194	+	2.46	-429	100154619	CLDND1	160397444	160405675
NC_010454.4	48761779	48762076	-	2.78	-1972	100513375	CLUH	48740117	48759955
NC_010445.4	128900572	128900803	+	-2.12	-2707	100623872	CMPK2	128903395	128917873
NC_010448.4	27392845	27393167	-	1.29	-1528	110260935	CMTM4	27319074	27391478
NC_010446.5	122505450	122505681	+	-1.12	1115	100049656	CNN3	122504450	122528351
NC_010445.4	56811538	56811826	-	0.46	434	100512161	CNNM3	56792389	56812116
NC_010450.4	73304649	73304947	-	2.46	96	100512848	CNOT6L	73191721	73304894
NC_010449.5	68598678	68598966	-	3.05	-3680	100624179	COCH	68580124	68595142
NC_010445.4	22740849	22741137	+	1.46	568	100627695	COG7	22740425	22826589
NC_010446.5	20143517	20143805	-	3.37	79	100622782	COLEC10	19692990	20143740
NC_010445.4	131257798	131258086	-	2.20	-3387	100736753	COLEC11	131226941	131254555

NC_010445.4	79913787	79914075	-	2.20	38	100521368	COMMD1	79770315	79913969
NC_010444.4	58931151	58931611	+	2.78	452	100525632	COPE	58930929	58948466
NC_010454.4	61002323	61002611	+	1.20	1905	100512312	COPS3	61000562	61023916
NC_010446.5	67740499	67740765	+	-1.76	104	100125955	COPS5	67740528	67750351
NC_010447.5	19522805	19523093	+	2.05	382	100156765	COPZ1	19522567	19542373
NC_010457.5	101339095	101339357	+	-0.95	1214	100152647	COQ10B	101338012	101354714
NC_010443.5	268756367	268756752	+	-1.93	-149	100156776	COQ4	268756709	268768355
NC_010443.5	240001303	240001534	-	-1.27	-454	100511910	CORO2A	239947299	240000964
NC_010443.5	90696945	90697188	+	-1.35	320	100038000	COX7A2	90696746	90703407
NC_010450.4	10403558	10403873	+	1.46	18	100462674	CPEB2	10403697	10472141
NC_010456.5	103838550	103838857	-	2.46	-1808	100152117	CPEB3	103675333	103836895
NC_010456.5	103838133	103838421	-	1.88	-1382	100152117	CPEB3	103675333	103836895
NC_010447.5	33263987	33264281	-	1.78	228	100628221	CPM	33170094	33264362
NC_010455.5	131340827	131341115	-	1.05	-3250	100627569	CPN2	131327508	131337721
NC_010457.5	110164517	110164805	+	0.88	-3951	100155330	CPO	110168612	110194156
NC_010445.4	30363195	30363605	+	-0.02	675	100627178	CPPED1	30362725	30484250
NC_010457.5	113213349	113213580	+	-1.71	3426	100157716	CPS1	113210038	113339086
NC_010457.5	113209203	113209665	+	0.82	-604	100157716	CPS1	113210038	113339086
NC_010445.4	6374793	6375081	+	2.46	575	100518830	CPSF4	6374362	6388662
NC_010447.5	33528079	33528310	+	0.63	2526	100152191	CPSF6	33525668	33559941
NC_010455.5	59759630	59759918	-	3.20	-1998	100737786	CRBN	59733503	59757776
NC_010445.4	16619263	16619661	-	0.46	2543	100517396	CRCP	16584304	16622005
NC_010458.4	51212928	51213358	-	3.63	-3194	100523409	CREBRF	51146015	51209949
NC_010445.4	52914216	52914619	+	-1.08	-4057	100517088	CREG2	52918475	52978020
NC_010445.4	93926042	93926363	-	1.05	-286	100524556	CRIPT	93913579	93925916
NC_010445.4	93929789	93930062	-	0.05	-4009	100524556	CRIPT	93913579	93925916
NC_010459.5	14860723	14861241	+	1.46	663	100153088	CRLS1	14860319	14892436
NC_010449.5	53146895	53147183	+	0.88	722	100621206	CRTC3	53146317	53255985
NC_010444.4	16624158	16624389	-	-0.54	-3937	100517750	CRY2	16587101	16620336
NC_010445.4	24814063	24814351	+	2.88	-803	100525333	CRYM	24815010	24835837
NC_010448.4	138434442	138434829	+	0.61	-324	733653	CRYZ	138434960	138461042
NC_010446.5	24852275	24852506	+	-0.35	-2929	100621741	CSMD3	24855320	25871456
NC_010443.5	107689152	107689440	+	2.29	-754	100157196	CSNK1G1	107690050	107884054
NC_010444.4	127017898	127018241	+	2.29	1041	100511866	CSNK1G3	127017028	127129547

NC_010448.4	129175031	129175339	+	-2.67	1786	100523254	CTBS	129173399	129207868
NC_010443.5	145811691	145812037	-	4.05	-191	100158188	CTDP1	145769771	145811673
NC_010443.5	127124615	127124970	-	0.36	954	102162809	CTDSPL2	127029445	127125747
NC_010444.4	48728283	48728659	-	-1.02	1009	100627268	CTR9	48700629	48729480
NC_010457.5	126285661	126285949	-	2.46	1343	100511787	CUL3	126191605	126287148
NC_010448.4	34120836	34121176	-	3.27	-513	100624570	CYLD	34058091	34120493
NC_010456.5	106666975	106667276	+	-0.86	680	403111	CYP2C42	106666445	106701809
NC_010449.5	41424896	41425250	-	-2.71	781	100113410	CYP39A1	41345011	41425854
NC_010449.5	35128129	35128417	+	1.88	1028	100155713	DAAM2	35127245	35285933
NC_010455.5	78969045	78969371	-	-0.24	1042	106505697	DBR1	78958301	78970250
NC_010446.5	117884427	117884735	+	1.78	-172	100156530	DBT	117884753	117924006
NC_010454.4	18344428	18344719	+	1.36	543	100521290	DCAKD	18344030	18362516
NC_010452.4	46815102	46815333	+	-0.35	-4798	100512855	DCLRE1C	46820016	46863848
NC_010444.4	116803910	116804141	-	-1.67	667	100516785	DCP2	116736800	116804693
NC_010444.4	116803571	116803802	-	1.46	1006	100516785	DCP2	116736800	116804693
NC_010444.4	151712861	151713411	-	0.32	787	100626043	DCTN4	151681003	151713923
NC_010449.5	23367425	23367656	+	-0.86	-3817	100144462	DDR1	23371358	23391579
NC_010445.4	121855646	121856013	-	4.05	837	100627404	DDX1	121818400	121856667
NC_010447.5	67364509	67364797	+	3.46	855	100515028	DDX11	67363798	67395739
NC_010447.5	9487086	9487545	+	1.46	1523	100514347	DDX17	9485792	9505157
NC_010456.5	72039970	72040460	+	2.10	230	100153111	DDX21	72039985	72063778
NC_010444.4	64840467	64840794	+	3.05	4590	100515361	DDX39A	64836040	64844996
NC_010454.4	14573819	14574050	+	-0.35	1203	102158045	DDX5	14572731	14580740
NC_010456.5	21083906	21084194	+	4.05	849	100158037	DDX60	21083201	21157001
NC_010446.5	89313089	89313377	+	2.46	1925	100156474	DEDD	89311308	89323692
NC_010448.4	49711808	49712180	+	1.10	1046	100516271	DEDD2	49710948	49730457
NC_010443.5	203599991	203600334	-	1.78	1135	100154094	DENND4C	203449832	203601298
NC_010447.5	42643371	42643659	+	2.05	838	102165384	DENND5B	42642677	42870980
NC_010448.4	144157251	144157984	+	1.78	48	110261209	DEPDC1	144157569	144183977
NC_010447.5	83877353	83877943	+	1.00	-1070	100511197	DEPDC4	83878718	83899408
NC_010444.4	27929026	27929314	-	2.20	-311	100621764	DEPDC7	27909282	27928859
NC_010446.5	19179660	19180251	-	2.20	175	100157658	DEPTOR	18976732	19180131
NC_010446.5	16309820	16310108	+	-0.12	-2672	100626802	DERL1	16312636	16341405
NC_010457.5	84159795	84160136	+	3.31	2101	100518993	DFNB59	84157864	84165763

NC_010456.5	51657491	51657865	-	3.27	-327	110256627	DGCR6L	51644840	51657351
NC_010453.5	24973995	24974301	-	2.20	-3220	100157742	DGKH	24772396	24970928
NC_010455.5	30601777	30602065	+	3.05	-118	100518989	DHX30	30602039	30632506
NC_010455.5	94349372	94349603	-	-1.86	845	100626674	DHX36	94298790	94350333
NC_010451.4	124142304	124142924	+	1.78	646	100514066	DHX9	124141968	124183838
NC_010461.5	78603832	78604063	+	-2.44	1562	100155257	DIAPH2	78602385	79520441
NC_010453.5	32994627	32994915	-	3.78	83	100737986	DIAPH3	32492735	32994854
NC_010455.5	137737106	137737394	-	3.05	1864	100519567	DIRC2	137633471	137739114
NC_010453.5	45075701	45076028	-	2.78	108	100521470	DIS3	45046040	45075973
NC_010443.5	130960526	130961111	-	2.20	1961	100156025	DISP2	130931909	130962780
NC_010451.4	107642728	107642967	+	0.46	718	397129	DLD	107642129	107668443
NC_010443.5	184550487	184550718	-	0.46	-574	100511173	DLGAP5	184500791	184550028
NC_010443.5	184548789	184549433	-	2.66	917	100511173	DLGAP5	184500791	184550028
NC_010445.4	37918409	37918948	-	1.10	425	100624182	DNAJA3	37883233	37919104
NC_010444.4	64762328	64762623	+	1.88	803	396741	DNAJB1	64761672	64764482
NC_010456.5	75188833	75189418	-	1.46	983	100156234	DNAJB12	75169076	75190109
NC_010448.4	135265067	135265355	-	2.46	-4886	100525703	DNAJB4	135243927	135260325
NC_010443.5	235789924	235790237	+	-1.54	-3302	100525553	DNAJB5	235793383	235799385
NC_010452.4	52864588	52864874	+	-0.95	1918	100519794	DNAJC1	52862813	53060747
NC_010452.4	52863689	52863984	+	1.95	1023	100519794	DNAJC1	52862813	53060747
NC_010455.5	118912988	118913276	-	1.88	138	100154656	DNAJC19	118907299	118913270
NC_010445.4	38646562	38646987	-	0.88	77	397051	DNASE1	38590045	38646852
NC_010451.4	114261514	114261802	+	2.27	-1161	100622420	DNM3	114262819	114607232
NC_010446.5	123394991	123395279	+	2.05	364	100157622	DNTTIP2	123394771	123408460
NC_010456.5	6180229	6180517	-	2.46	522	100152025	DOK2	6176487	6180895
NC_010443.5	153396197	153396498	-	1.61	-2915	100517871	DOK6	152990887	153393432
NC_010446.5	94662204	94662589	+	2.22	828	100153065	DPM3	94661568	94662223
NC_010444.4	5946264	5946596	-	1.46	1589	100519350	DPP3	5907251	5948019
NC_010457.5	68742943	68743231	-	1.78	-126	397492	DPP4	68661764	68742961
NC_010446.5	123835000	123835351	-	1.05	699	100152776	DR1	123815088	123835875
NC_010448.4	115401517	115401934	+	1.14	1502	100625833	DSG2	115400223	115453763
NC_010457.5	88549388	88549975	+	1.56	431	100523472	DUSP19	88549250	88574978
NC_010456.5	120906460	120906748	+	3.20	264	100157144	DUSP5	120906340	120919398
NC_010455.5	122076607	122076995	+	1.27	947	100621038	DVL3	122075854	122092975

NC_010451.4	75451391	75451625	+	-0.35	4412	100523985	DYNC1I1	75447096	75760522
NC_010457.5	77803281	77803569	+	0.73	-176	100154566	DYNC1I2	77803601	77859559
NC_010457.5	77804528	77805251	+	1.46	1288	100154566	DYNC1I2	77803601	77859559
NC_010448.4	7661052	7661340	-	2.68	184	102165562	DYNLRB2	7650444	7661380
NC_010457.5	109972902	109973242	-	2.20	3316	100517128	DYTN	109918321	109976388
NC_010453.5	65205787	65206018	-	-0.44	-1608	100519383	DZIP1	65147003	65204294
NC_010455.5	2655595	2655883	+	-0.86	910	100517678	EAF1	2654829	2671140
NC_010445.4	22655604	22655911	+	-0.12	342	100523812	EARS2	22655415	22684246
NC_010461.5	55771121	55771429	+	1.78	317	100518887	EDA	55770958	56103296
NC_010456.5	135046779	135047067	+	0.88	4841	100522106	EDRF1	135042082	135085552
NC_010447.5	90132622	90132910	+	1.78	1059	100153403	EEA1	90131707	90257014
NC_010447.5	90131503	90131903	+	3.05	-4	100153403	EEA1	90131707	90257014
NC_010456.5	134152136	134152367	-	-1.71	2414	100521159	EEF1AKMT2	134134899	134154666
NC_010448.4	164777680	164778077	+	2.05	-97	100526227	EFCAB14	164777976	164821371
NC_010444.4	6454841	6455129	+	3.05	-170	100526031	EFEMP2	6455155	6463493
NC_010446.5	94670055	94670551	-	3.27	2179	100144501	EFNA1	94666457	94672482
NC_010453.5	74233920	74234208	-	3.46	-1296	100135673	EFNB2	74188337	74232768
NC_010446.5	9136602	9136964	-	1.27	469	100627646	EFR3A	9053445	9137252
NC_010456.5	59114785	59115119	+	2.20	1416	100153461	EGLN1	59113536	59168421
NC_010456.5	66289232	66289549	-	1.63	1917	100038004	EGR2	66280665	66291308
NC_010445.4	79256675	79256978	-	-2.86	933	100520668	EHBP1	78907315	79257760
NC_010443.5	131355929	131356160	-	-2.93	-819	100621160	EIF2AK4	131244818	131355225
NC_010456.5	129006066	129006297	-	-0.12	986	100156570	EIF3A	128967645	129007168
NC_010456.5	129005004	129005292	-	2.27	2020	100156570	EIF3A	128967645	129007168
NC_010447.5	11213430	11213859	+	3.63	332	100512835	EIF3D	11213312	11229158
NC_010446.5	29288810	29289102	+	-2.12	78	100156671	EIF3E	29288878	29343593
NC_010448.4	47476302	47476590	+	1.46	258	100517098	EIF3K	47476188	47488933
NC_010444.4	28281906	28282194	-	0.73	762	100513781	EIF3M	28258399	28282812
NC_010455.5	124585128	124585359	+	-1.93	2421	100101928	EIF4A2	124582822	124589296
NC_010455.5	124582323	124583035	+	-0.27	-143	100101928	EIF4A2	124582822	124589296
NC_010445.4	11322753	11323229	+	1.29	1505	100627717	EIF4H	11321486	11348473
NC_010455.5	109527949	109528237	-	3.20	2357	106505742	EIF5A2	109510815	109530450
NC_010444.4	70134954	70135242	-	1.46	-750	100515074	ELAVL3	70114475	70134348
NC_010450.4	87855835	87856173	+	2.88	2977	100514064	ELF2	87853027	87878847

NC_010444.4	102590802	102591090	-	0.29	470	100523777	ELL2	102518605	102591416
NC_010446.5	61970093	61970381	+	1.29	-866	100153381	ELOC	61971103	61989392
NC_010450.4	112038919	112039207	+	4.27	393	100312970	ELOVL6	112038670	112190075
NC_010458.4	39678231	39678462	-	-1.93	1395	100524592	ELOVL7	39588122	39679742
NC_010444.4	29229420	29229651	-	-2.67	556	100514702	ELP4	29119345	29230092
NC_010449.5	110702497	110702815	-	-0.54	2796	100153276	EML5	110511937	110705452
NC_010452.4	13498876	13499180	-	3.05	2911	100525245	ENAH	13439772	13501939
NC_010453.5	23234648	23234888	+	-1.12	4	100525605	ENOX1	23234764	23894763
NC_010446.5	28199106	28199445	-	-0.12	4273	100153859	ENY2	28193634	28203549
NC_010446.5	28203223	28203568	-	1.68	153	100153859	ENY2	28193634	28203549
NC_010447.5	7395797	7396085	-	2.05	-1426	100156226	EP300	7311165	7394515
NC_010447.5	7392135	7392697	-	1.00	2099	100156226	EP300	7311165	7394515
NC_010457.5	3524676	3525119	-	1.27	1325	100518870	EPC2	3417446	3526223
NC_010445.4	93183781	93184221	-	0.29	1220	403163	EPCAM	93169800	93185221
NC_010443.5	95615262	95615539	-	-1.12	1393	100518639	EPG5	95493779	95616794
NC_010448.4	75278108	75278396	-	2.05	1112	100522873	EPHA2	75251465	75279364
NC_010453.5	24121019	24121250	+	-1.12	4143	100625050	EPSTI1	24116991	24216219
NC_010454.4	45082819	45083107	+	1.46	32	100518578	ERAL1	45082931	45088674
NC_010454.4	45085437	45085725	+	4.05	2650	100518578	ERAL1	45082931	45088674
NC_010447.5	21474602	21474892	+	1.88	529	100525045	ERBB3	21474218	21495040
NC_010449.5	93083393	93083765	-	1.78	371	100152505	ERH	93068254	93083950
NC_010448.4	106787435	106788089	-	2.46	1683	100524501	ESCO1	106697975	106789445
NC_010446.5	42061530	42061818	-	1.46	32	100512961	ESRP1	41993944	42061706
NC_010447.5	21518868	21519156	+	2.05	1056	100519485	ESYT1	21517956	21535320
NC_010447.5	60775124	60775407	-	-2.12	1525	100157726	ETV6	60523530	60776791
NC_010447.5	60777258	60777757	-	1.05	-716	100157726	ETV6	60523530	60776791
NC_010446.5	124408579	124408867	+	1.78	4208	100519143	EVI5	124404515	124600580
NC_010456.5	46408523	46408811	+	1.78	31	100156911	EWSR1	46408636	46439357
NC_010443.5	270748651	270749047	+	1.27	315	100523112	EXOSC2	270748534	270757717
NC_010446.5	20649202	20649440	+	-1.54	4319	100157655	EXT1	20645002	20935769
NC_010459.5	9075754	9075985	-	-1.67	-2317	110257404	F11	9045990	9073552
NW_018085100.1	2586336	2586686	+	1.61	4568	100627330	F13B	2581943	2614678
NC_010450.4	104465466	104465697	+	-2.12	1239	595106	FABP2	104464342	104468059
NC_010448.4	161206505	161206793	+	2.14	1296	100518974	FAF1	161205353	161603727

NC_010458.4	69123335	69123623	+	2.27	1734	100511051	FAM114A2	69121745	69159398
NC_010451.4	91821251	91821634	-	1.68	186	100620255	FAM126A	91729044	91821629
NC_010461.5	76150010	76150241	+	1.14	-113	100519754	FAM133A	76150239	76181971
NC_010444.4	89112862	89113281	+	-3.22	3693	100511985	FAM151B	89109378	89144270
NC_010455.5	138148780	138149011	-	-0.93	2816	100627352	FAM162A	138115499	138151712
NC_010444.4	100893880	100894168	-	0.14	-436	100521436	FAM172A	100477770	100893588
NC_010443.5	144783191	144783423	-	-0.95	4784	110261534	FAM189A1	144316119	144788091
NC_010458.4	53935322	53935553	+	-2.12	-4049	100511664	FAM196B	53939487	54057671
NC_010455.5	49445961	49446192	+	-1.35	2350	100522704	FAM19A1	49443726	49970760
NC_010450.4	10990741	10991029	+	-0.54	-263	102160065	FAM200B	10991148	11004815
NC_010455.5	207557618	207557906	+	1.05	-3118	100623984	FAM207A	207560880	207588502
NC_010455.5	29570931	29571260	+	2.68	1039	106505592	FAM240A	29570056	29574895
NC_010455.5	29572028	29572259	+	-0.12	2087	106505592	FAM240A	29570056	29574895
NC_010443.5	82086672	82086960	+	1.05	869	100158219	FAM26F	82085947	82087897
NC_010455.5	204746650	204746983	+	2.68	-2336	100517916	FAM3B	204749153	204796792
NC_010448.4	84308580	84308871	-	2.20	2231	100511570	FAM46B	84303495	84310957
NC_010445.4	120968329	120968617	+	3.78	457	100518011	FAM49A	120968016	121069372
NC_010451.4	27813566	27813854	-	3.46	-292	100517546	FAM76B	27790139	27813418
NC_010445.4	122717533	122717764	-	-0.44	3308	100518964	FAM84A	122716029	122720957
NC_010446.5	13446921	13447296	+	3.05	1825	100623936	FAM84B	13445283	13451329
NC_010443.5	132819123	132819411	-	1.73	24	100522915	FAM98B	132786198	132819291
NC_010452.4	26796856	26797358	+	2.29	255	102158362	FANCC	26796852	27055271
NC_010452.4	26795694	26795925	+	-1.54	-1042	102158362	FANCC	26796852	27055271
NC_010452.4	27584019	27584250	+	-1.35	-4474	397038	FBP1	27588609	27622225
NC_010445.4	17744123	17744411	-	1.61	-456	102159616	FBRS	17731276	17743811
NC_010444.4	113478500	113478801	-	-2.44	1913	100513341	FBXL17	112989134	113480564
NC_010443.5	65934615	65934903	-	2.31	944	100156082	FBXL4	65872119	65935703
NC_010443.5	65937570	65937822	-	-1.35	-1993	100156082	FBXL4	65872119	65935703
NC_010443.5	65936340	65936796	-	2.27	-865	100156082	FBXL4	65872119	65935703
NC_010443.5	65935654	65936039	-	0.36	-143	100156082	FBXL4	65872119	65935703
NC_010445.4	92697858	92698146	+	1.46	1600	100624629	FBXO11	92696402	92792623
NC_010443.5	149856644	149856946	+	1.05	763	100516237	FBXO15	149856032	149897309
NC_010443.5	184667465	184667753	+	1.46	1749	100511360	FBXO34	184665860	184689953
NC_010458.4	26708115	26708489	+	0.78	832	100522958	FBXO4	26707470	26723765

NC_010444.4	82100934	82101165	+	-1.12	-245	100514094	FCHO2	82101295	82248410
NC_010459.5	15137893	15138242	-	2.68	-534	100157961	FERMT1	15080585	15137533
NC_010443.5	182608746	182609024	-	-1.27	-1995	100517989	FERMT2	182519681	182606890
NC_010453.5	70858022	70858253	-	0.14	1279	100156844	FGF14	70251374	70859417
NC_010445.4	49417757	49418051	+	2.46	2173	100513532	FHL2	49415731	49448459
NC_010444.4	33327124	33327355	-	1.05	4512	100620302	FIBIN	33328250	33331752
NC_010444.4	33331731	33332039	-	2.27	-133	100620302	FIBIN	33328250	33331752
NC_010444.4	6440296	6440584	+	1.05	-43	100525557	FIBP	6440483	6445927
NC_010443.5	75694291	75694689	+	2.31	301	100511669	FIG4	75694189	75792976
NC_010449.5	31548139	31548427	-	1.78	1707	100155423	FKBP5	31441765	31549990
NC_010457.5	84182310	84182857	-	1.20	286	100144630	FKBP7	84168483	84182870
NC_010459.5	22953938	22954379	-	2.63	1117	100158214	FLRT3	22940491	22955276
NC_010453.5	5427323	5427611	-	3.20	2631	100515445	FLT3	5370475	5430098
NC_010446.5	123775814	123776045	-	-0.35	3710	100157977	FNBP1L	123675062	123779640
NC_010445.4	110841152	110841440	-	2.05	1420	100623777	FOSL2	110820403	110842716
NC_010449.5	62475653	62475975	+	2.78	4581	100156502	FOXA1	62471233	62479638
NC_010459.5	30002484	30003029	-	0.73	-1945	100513828	FOXA2	29997397	30000811
NC_010454.4	5308035	5308335	+	3.78	815	100623071	FOXJ1	5307370	5312117
NC_010447.5	63062643	63062931	+	1.20	809	100625827	FOXJ2	63061978	63087689
NC_010461.5	42609374	42609662	+	2.46	-42	100431100	FTSJ1	42609560	42620157
NC_010448.4	135282298	135283081	+	-1.80	1215	100525995	FUBP1	135281474	135318715
NW_018085072.1	117172	117421	-	0.46	-920	100625870	FUT10	34820	116376
NC_010451.4	20721048	20721336	-	3.46	268	100520319	FZD4	20714995	20721460
NC_010446.5	33846803	33847150	-	3.37	-2420	100157360	FZD6	33811159	33844556
NC_010452.4	58093033	58093321	-	0.88	3613	110255708	FZD8	58093980	58096790
NC_010452.4	58096734	58097022	-	2.46	-88	110255708	FZD8	58093980	58096790
NC_010443.5	121413557	121413871	+	2.68	3438	100157164	GABPB1	121410276	121488165
NC_010443.5	121413972	121414260	+	1.46	3840	100157164	GABPB1	121410276	121488165
NC_010456.5	60139069	60139665	-	1.53	1814	100153028	GALNT2	59961279	60141181
NC_010455.5	144457909	144458197	-	2.05	83	100623423	GAP43	144364314	144458136
NC_010456.5	14886358	14886593	+	-1.12	2822	397555	GATA4	14883653	14938156
NC_010456.5	14887702	14888017	+	1.46	4206	397555	GATA4	14883653	14938156
NC_010459.5	46705495	46705783	+	2.78	-3621	100625236	GDAP1L1	46709260	46733275
NC_010447.5	21189854	21190193	+	2.05	-1364	397254	GDF11	21191388	21199024

NC_010452.4	65163907	65164237	+	-1.86	3372	414427	GDI2	65160700	65196447
NC_010458.4	68279187	68279475	+	1.78	110	100524914	GEMIN5	68279221	68330029
NC_010458.4	68280168	68280456	+	2.46	1091	100524914	GEMIN5	68279221	68330029
NC_010445.4	73030200	73030488	+	2.78	1594	397209	GFPT1	73028750	73086191
NC_010445.4	73028316	73028604	+	-0.12	-290	397209	GFPT1	73028750	73086191
NC_010445.4	59202296	59202584	+	1.46	77	100620337	GGCX	59202363	59219807
NC_010448.4	20163184	20163501	+	-1.03	1078	100512472	GINS3	20162264	20170385
NC_010448.4	20158492	20158723	+	-2.18	-3656	100512472	GINS3	20162264	20170385
NC_010446.5	99722477	99722765	-	3.78	2322	100736742	GJA8	99721754	99724943
NC_010457.5	30581075	30581306	-	-1.71	-918	100520334	GLI2	30312543	30580272
NC_010447.5	38683541	38683838	+	-2.12	3206	100737573	GLIPR1L2	38680483	38710487
NC_010457.5	95514822	95515335	+	2.05	1664	399525	GLS	95513414	95606029
NC_010456.5	88006022	88006356	-	0.24	-814	100157162	GLUD1	87968850	88005375
NC_010445.4	72619897	72620380	-	-0.41	-442	100511872	GMCL1	72570426	72619696
NC_010448.4	85657117	85657348	+	-0.12	-886	110261126	GMEB1	85658119	85701211
NC_010449.5	19585475	19585763	+	2.46	-358	100515035	GMNN	19585977	19595346
NC_010449.5	19585962	19586250	+	1.46	129	100515035	GMNN	19585977	19595346
NC_010451.4	100270065	100270482	-	2.46	2000	100144419	GNAI1	100187878	100272274
NC_010446.5	110578940	110579228	-	1.46	247	100144421	GNAI3	110527363	110579331
NC_010455.5	32672125	32672413	+	2.20	892	100157237	GNAT1	32671377	32678394
NC_010448.4	63952518	63952806	-	2.46	-66	110261346	GNB1	63867155	63952596
NC_010448.4	129248853	129249084	+	-1.35	1644	102166994	GNG5	129247324	129254973
NC_010456.5	59239878	59240350	-	3.27	189	100156253	GNPAT	59203519	59240303
NC_010456.5	22689299	22689587	+	1.14	103	100155657	GOLGA3	22689340	22738544
NC_010455.5	138750237	138750468	+	-2.08	-4450	100620515	GOLGB1	138754803	138847254
NC_010455.5	106892060	106892348	-	-0.27	3005	100517915	GOLIM4	106814825	106895209
NC_010455.5	106892453	106892747	-	-1.60	2609	100517915	GOLIM4	106814825	106895209
NC_010447.5	51952286	51952544	-	-2.93	899	100154676	GOLT1B	51939480	51953314
NC_010443.5	44238910	44239198	+	0.14	-310	100520524	GOPC	44239364	44285886
NC_010449.5	99326313	99326544	+	-2.95	844	100153141	GPATCH2L	99325584	99376291
NC_010458.4	36218610	36218841	+	-0.54	1490	100521344	GPBP1	36217235	36298003
NC_010458.4	36218854	36219318	+	2.70	1851	100521344	GPBP1	36217235	36298003
NC_010447.5	16007689	16008035	+	1.29	-317	100153250	GPD1	16008179	16021676
NC_010457.5	80461273	80461663	-	1.29	603	100511231	GPR155	80417247	80462071

NC_010445.4	49539644	49539875	-	-1.71	-3723	100736943	GPR45	49531595	49536036
NC_010443.5	63973445	63973882	-	3.27	751	100152431	GPR63	63911304	63974415
NC_010445.4	87339995	87340226	+	-1.71	-1509	100517153	GPR75	87341620	87350664
NC_010447.5	59537752	59538044	+	-0.12	2985	100739328	GPRC5D	59534913	59545417
NC_010446.5	111164762	111165050	-	1.73	996	100518248	GPSM2	111100912	111165902
NC_010446.5	111161073	111161362	-	1.46	4684	100518248	GPSM2	111100912	111165902
NC_010446.5	111164338	111164626	-	2.20	1420	100518248	GPSM2	111100912	111165902
NC_010455.5	31921078	31921414	-	2.20	-3909	397403	GPX1	31916269	31917337
NC_010455.5	146043877	146044173	-	-0.95	21	100513240	GRAMD1C	145997738	146044046
NC_010454.4	5940876	5941255	+	2.20	342	100192436	GRB2	5940723	6009095
NC_010456.5	87227128	87227416	-	2.20	-228	100153116	GRID1	86539896	87227044
NC_010443.5	68050505	68050793	+	2.20	-2389	100516526	GRIK2	68053038	68679088
NC_010450.4	67608206	67608437	-	0.14	-899	100516986	GRSF1	67586935	67607422
NC_010449.5	46657693	46657990	-	1.46	-1350	100152951	GSTA4	46635903	46656491
NC_010449.5	46656361	46656696	-	2.88	-37	100152951	GSTA4	46635903	46656491
NC_010443.5	112371554	112371878	+	3.27	424	100521701	GTF2A2	112371292	112399541
NC_010457.5	54445228	54445459	+	-2.54	863	100513628	GTF2E2	54444480	54525566
NC_010444.4	40899049	40899337	-	2.78	1398	100525504	GTF2H1	40857720	40900591
NC_010445.4	19369028	19369260	+	0.46	747	100519247	GTF3C1	19368397	19439640
NC_010447.5	9323133	9323421	-	2.78	-1085	100520611	GTPBP1	9294329	9322192
NC_010444.4	60355670	60355958	-	2.31	-411	110259323	GTPBP3	60352012	60355403
NC_010450.4	35167208	35167866	+	2.31	-78	100516681	GUF1	35167615	35190450
NC_010452.4	13941697	13941994	+	2.88	1907	396970	H3F3A	13939938	13947976
NC_010455.5	2841097	2841568	-	-0.86	-459	100518218	HACL1	2786007	2840873
NC_010455.5	2840459	2840747	-	1.88	270	100518218	HACL1	2786007	2840873
NC_010450.4	114004960	114005478	-	2.10	291	397604	HADH	113952561	114005510
NC_010444.4	60567792	60568080	+	1.88	361	100516543	HAUS8	60567575	60593378
NC_010444.4	142114928	142115471	-	3.05	-7	397564	HBEGF	142102455	142115192
NC_010445.4	41479531	41479819	-	0.88	477	100738328	HBQ1	41478282	41480152
NC_010443.5	28969279	28969510	+	-0.12	616	100158211	HBS1L	28968778	29052425
NC_010447.5	80409049	80409337	-	1.88	605	100514768	HCFC2	80366826	80409798
NC_010443.5	96724393	96724624	-	-2.44	4986	100521177	HDHD2	96682632	96729495
NC_010456.5	103095283	103095571	+	3.46	-265	100156944	HECTD2	103095692	103182111
NC_010456.5	105965783	105966229	-	1.98	997	100154547	HELLS	105923727	105967003

NC_010443.5	108207086	108207395	+	0.05	1055	100153537	HERC1	108206185	108404149
NC_010450.4	130666376	130666607	-	1.46	-901	100626657	HERC6	130594941	130665590
NC_010455.5	131136041	131136272	+	-1.71	186	100499567	HES1	131135970	131137865
NC_010444.4	83745709	83746046	+	2.20	614	396958	HEXB	83745263	83784762
NC_010450.4	83680390	83680621	-	-1.35	-2142	100525059	HHIP	83583735	83678363
NC_010455.5	151231294	151231734	-	-0.68	3315	102166962	HHLA2	151153982	151234829
NC_010443.5	22239518	22239869	+	3.46	-2891	100525369	HIVEP2	22242585	22436940
NC_010449.5	20998671	20998959	+	-0.12	-98	100157353	HMGN4	20998913	21003138
NC_010457.5	13768234	13768555	-	2.68	-288	100624677	HNMT	13733412	13768106
NC_010447.5	19488502	19488824	+	-1.12	3630	768103	HNRNPA1	19485033	19489559
NC_010447.5	19486015	19486357	+	-2.12	1153	768103	HNRNPA1	19485033	19489559
NC_010460.4	46205331	46205646	+	0.88	324	100521762	HNRNPA2B1	46205164	46215854
NC_010460.4	46206234	46206718	+	1.78	1312	100521762	HNRNPA2B1	46205164	46215854
NC_010457.5	82960144	82960406	+	-1.12	1946	100516520	HNRNPA3	82958329	82968740
NC_010456.5	71485099	71485332	+	-1.44	-1085	100155513	HNRNPH3	71486301	71498356
NC_010448.4	47654511	47654799	-	2.20	1201	102163392	HNRNPL	47643561	47655856
NC_010448.4	81238370	81238665	-	-2.29	1305	100620404	HNRNPR	81203079	81239823
NC_010448.4	49279610	49280079	+	3.27	-451	100622274	HNRNPUL1	49280296	49315967
NC_010444.4	9037283	9037514	+	-2.54	2729	100620658	HNRNPUL2	9034669	9048906
NC_010444.4	9036643	9036992	+	2.27	2148	100620658	HNRNPUL2	9034669	9048906
NC_010444.4	88244975	88245479	-	-3.65	778	100511070	HOMER1	88116664	88246005
NC_010448.4	109299025	109299348	+	3.46	-3077	397224	HRH4	109302264	109318019
NC_010443.5	40179194	40179560	-	2.85	931	100154234	HSF2	40147267	40180308
NC_010447.5	80527518	80527806	-	1.88	3354	397191	HSP90B1	80511513	80531016
NC_010449.5	88606571	88607277	+	2.09	-216	100621324	HSPA2	88607140	88609947
NC_010457.5	101375562	101375825	+	0.46	1674	397575	HSPE1	101374019	101376146
NC_010457.5	101374375	101374721	+	2.05	529	397575	HSPE1	101374019	101376146
NC_010453.5	7759943	7760327	-	1.05	-700	100048931	HSPH1	7733880	7759435
NC_010461.5	94051997	94052523	+	2.88	-1155	100524920	HTR2C	94053415	94313352
NC_010461.5	46443390	46443621	-	1.24	-1678	100517442	HUWE1	46281962	46441827
NC_010448.4	14311441	14311672	-	-1.86	-3766	100513208	HYDIN	13895276	14307790
NC_010448.4	14305557	14305845	-	1.68	2089	100513208	HYDIN	13895276	14307790
NC_010445.4	42022125	42022368	-	-1.08	340	100624746	IARS	41945846	42022587
NC_010452.4	31340377	31340996	-	2.46	94	100622692	IDNK	31329716	31340781

NC_010444.4	65912756	65913044	-	1.20	-409	100525209	IER2	65909638	65912491
NC_010443.5	96760194	96760575	-	3.05	3490	100521060	IER3IP1	96740718	96763875
NC_010448.4	134648041	134648329	-	1.20	1672	100525523	IFI44	134634679	134649857
NC_010448.4	134678684	134679023	-	2.05	358	100511267	IFI44L	134660230	134679212
NC_010457.5	68983635	68984014	-	-0.12	1320	100101927	IFIH1	68930998	68985145
NC_010456.5	101237408	101237675	+	-1.71	138	100153038	IFIT1	101237403	101249486
NC_010455.5	196802094	196802437	+	3.05	290	100533555	IFNAR2	196801975	196852640
NC_010443.5	201278204	201278592	-	2.27	-97	100153693	IFN-DELTA-8	201277798	201278301
NC_010443.5	201338489	201338720	-	-1.93	-3666	100156149	IFN-OMEGA-4	201334366	201334938
NC_010443.5	201313607	201313838	-	0.24	3838	100154075	IFN-OMEGA-7	201316989	201317561
NC_010443.5	201319420	201319651	-	-1.12	-1974	100154075	IFN-OMEGA-7	201316989	201317561
NC_010443.5	137692968	137693256	-	1.46	-3263	397350	IGF1R	137383623	137689849
NC_010443.5	137692179	137692493	-	1.61	-2487	397350	IGF1R	137383623	137689849
NC_010451.4	92275193	92275636	-	1.73	-3150	100621977	IGF2BP3	92104786	92272264
NC_010455.5	91682273	91682504	-	-2.35	-2894	100524956	IGSF10	91625421	91679494
NC_010455.5	91676160	91676448	-	1.14	3190	100524956	IGSF10	91625421	91679494
NC_010455.5	141145455	141145796	+	0.88	-2863	100511781	IGSF11	141148489	141289504
NC_010451.4	136375064	136375352	+	1.78	-149	100515333	IKZF1	136375357	136464942
NC_010457.5	115658028	115658454	-	1.95	-2728	100737978	IKZF2	115512160	115655513
NC_010450.4	101535784	101536015	+	-0.54	3157	403123	IL21	101532742	101540712
NC_010458.4	35150708	35150939	-	-1.67	1008	100037294	IL6ST	35101306	35151832
NC_010457.5	137764775	137765063	-	2.46	839	100518585	ILKAP	137740291	137765758
NC_010457.5	31974949	31975237	-	1.78	65	100621511	IMP4	31970179	31975158
NC_010456.5	114226478	114227016	+	2.46	-1251	100153400	INA	114227998	114240566
NC_010443.5	130989915	130990338	-	2.14	2465	106506757	INAFM2	130989737	130992592
NC_010444.4	9445935	9446166	-	0.46	-225	100516847	INCENP	9411942	9445825
NC_010457.5	30999303	30999610	-	1.05	4948	397490	INHBB	30999994	31004405
NC_010443.5	253396890	253397121	-	-1.12	-3119	100513906	INIP	253375193	253393886
NC_010457.5	94963740	94964028	+	3.05	-1359	100514878	INPP1	94965243	94987286
NC_010453.5	16374336	16374726	+	-0.12	-745	100156695	INTS6	16375276	16468246
NC_010450.4	97204164	97204732	-	2.39	211	100518740	INTU	97123062	97204659
NC_010443.5	241902435	241902666	+	-1.93	4825	100155489	INVS	241897725	242058121
NC_010453.5	67019971	67020371	+	-2.71	872	100152933	IPO5	67019299	67059375
NC_010455.5	71238469	71238757	+	2.68	4096	100622196	IQSEC1	71234517	71366017

NC_010449.5	47527972	47528263	-	0.20	1081	100153993	IREB2	47481539	47529199
NC_010449.5	47528602	47528971	-	2.46	412	100153993	IREB2	47481539	47529199
NC_010449.5	115352912	115353143	+	-0.12	-3591	100153902	ISG12(A)	115356619	115361537
NC_010459.5	21947515	21947803	+	2.78	-692	100154170	ISM1	21948351	22037023
NC_010457.5	91607106	91607449	+	-2.27	2600	397285	ITGAV	91604677	91711843
NC_010457.5	91603989	91604277	+	1.46	-544	397285	ITGAV	91604677	91711843
NC_010453.5	19374947	19375322	-	3.27	2796	595120	ITM2B	19349088	19377931
NC_010459.5	32474652	32475176	-	0.88	-87	100157088	ITPA	32458351	32474827
NC_010449.5	114518684	114518972	-	1.78	1977	100152339	ITPK1	114361976	114520805
NC_010451.4	126484852	126485109	-	-2.12	-4854	100302027	IVNS1ABP	126459542	126480126
NC_010448.4	147430046	147430596	+	1.82	1309	397202	JAK1	147429012	147567188
NC_010456.5	66729583	66729814	-	-1.35	-16	100157328	JMJD1C	66640845	66729682
NC_010446.5	61630097	61630421	+	1.53	1955	100155368	JPH1	61628304	61716550
NC_010446.5	61626742	61627030	+	0.98	-1418	100155368	JPH1	61628304	61716550
NC_010446.5	95713827	95714144	+	1.46	291	100145894	JTB	95713694	95716154
NC_010454.4	16936670	16937120	+	1.46	1890	100517913	KANSL1	16935005	17096906
NC_010455.5	6986662	6987153	+	1.68	1756	100626923	KAT2B	6985151	7099771
NC_010455.5	6984268	6984499	+	-0.86	-767	100626923	KAT2B	6985151	7099771
NC_010444.4	6571500	6571788	-	2.78	817	100511913	KAT5	6560544	6572461
NC_010443.5	16330681	16331093	+	3.05	33	100518101	KATNA1	16330854	16360801
NC_010444.4	15036013	15036728	+	2.31	-348	100511244	KBTBD4	15036719	15043825
NC_010460.4	26619947	26620178	-	-0.12	4147	100512382	KCND2	26139457	26624210
NC_010449.5	34654930	34655218	-	1.88	1631	100154866	KCNK5	34617948	34656705
NC_010444.4	59817789	59818098	-	3.46	-3128	100519132	KCNN1	59780010	59814815
NC_010444.4	118441547	118441778	+	-3.12	2334	100519705	KCNN2	118439328	118882299
NC_010445.4	18141227	18141570	-	3.46	190	100623749	KCTD13	18124628	18141589
NC_010455.5	40211065	40211353	+	1.46	434	100516225	KCTD6	40210775	40220091
NC_010448.4	84263550	84263859	-	2.78	1231	100511202	KDF1	84254493	84264936
NC_010444.4	5255033	5255264	-	-0.18	3438	100294703	KDM2A	5238690	5258587
NC_010448.4	167642371	167642707	-	2.46	-158	100621904	KDM4A	167597954	167642381
NC_010452.4	24831164	24831454	-	4.05	228	102163404	KDM5B	24739115	24831537
NC_010444.4	72658218	72658673	+	-2.00	1494	100516780	KHSRP	72656951	72668721
NC_010448.4	3786085	3786373	-	2.05	55	100515140	KIAA0513	3726752	3786284
NC_010450.4	101936510	101937192	-	1.63	301	100521104	KIAA1109	101737084	101937152

NC_010447.5	42297781	42298069	-	2.05	1670	100511327	KIAA1551	42263569	42299595
NC_010447.5	42298658	42298960	-	2.68	786	100511327	KIAA1551	42263569	42299595
NC_010443.5	216114645	216114934	+	-1.93	1867	100520352	KIAA2026	216112922	216226158
NC_010456.5	13098717	13099056	-	2.63	1556	100155514	KIF13B	12913615	13100443
NC_010452.4	42693040	42693328	-	2.20	367	595132	KIF5B	42637105	42693551
NC_010446.5	331866	332154	-	0.46	32	100514226	KIFC2	324623	332042
NC_010443.5	143538124	143538355	-	-1.71	1993	494563	KLF13	143499136	143540233
NC_010457.5	110378648	110378936	-	0.46	578	100038003	KLF7	110282404	110379370
NC_010460.4	18723511	18723758	-	-0.12	-544	100739403	KLHDC10	18667377	18723090
NC_010452.4	24900976	24901355	-	-1.86	1815	100526239	KLHL12	24869819	24902981
NC_010448.4	116612905	116613193	-	1.46	270	100518133	KLHL14	116510854	116613319
NC_010461.5	20128699	20128987	-	2.78	747	100525794	KLHL15	20088208	20129590
NC_010450.4	43870202	43870490	-	3.46	665	100511391	KLHL2	43749423	43871011
NC_010457.5	76029886	76030174	+	1.78	574	100520221	KLHL23	76029456	76051670
NC_010449.5	88292328	88293081	-	1.56	-746	100621005	KLHL25	88249009	88291958
NC_010449.5	88291740	88292098	-	3.05	39	100621005	KLHL25	88249009	88291958
NC_010444.4	59193359	59193647	-	2.78	445	100523486	KLHL26	59166118	59193948
NC_010457.5	75872625	75872996	+	2.46	-2883	100627572	KLHL41	75875694	75893789
NC_010447.5	62044183	62044622	-	3.20	453	100144592	KLRF1	62032559	62044856
NC_010448.4	59515219	59515507	+	0.46	-1695	110261054	KMT5C	59517058	59524551
NC_010453.5	17949392	17949623	+	-1.93	886	396715	KPNA3	17948621	18033818
NC_010453.5	17949017	17949381	+	2.46	578	396715	KPNA3	17948621	18033818
NC_010448.4	88610879	88611202	+	-0.22	-87	100621713	KPNA6	88611128	88656281
NC_010448.4	88612037	88612384	+	1.46	1082	100621713	KPNA6	88611128	88656281
NC_010445.4	57927822	57928110	+	2.05	-302	100515537	KRCC1	57928268	57946294
NC_010446.5	93975655	93975886	-	-0.54	-209	100156749	LAMTOR2	93972112	93975561
NC_010445.4	118153682	118153997	+	0.46	1973	100516188	LAPTM4A	118151866	118170279
NC_010452.4	68657670	68657930	+	-2.71	-1648	100522458	LARP4B	68659448	68707893
NC_010450.4	110089299	110089707	-	1.73	286	100511273	LARP7	110069565	110089789
NC_010450.4	110088942	110089239	-	0.05	698	100511273	LARP7	110069565	110089789
NC_010455.5	28524537	28524825	+	1.46	-97	100513625	LARS2	28524778	28692327
NW_018084833.1	1042156	1042812	+	4.08	-1506	100303611	LCN6	1043990	1048569
NC_010444.4	25140474	25141022	-	4.05	-2115	100511916	LDLRAD3	24875379	25138633
NC_010461.5	115582255	115582545	+	-0.71	1778	100525445	LDOC1	115580622	115583129

NC_010447.5	29597630	29597918	+	0.88	1440	100511508	LEMD3	29596334	29672704
NC_010457.5	54929455	54929743	-	2.46	259	100622960	LEPROTL1	54916037	54929858
NC_010450.4	927492	927780	-	0.88	-1136	100514302	LETM1	898178	926500
NW_018085018.1	328983	329271	-	3.05	-3966	110258454	LETMD1	313657	325161
NC_010449.5	114210185	114210563	-	1.88	-278	100154477	LGMN	114176217	114210096
NC_010457.5	59682373	59682604	+	-1.67	-2415	100517009	LIMS2	59684904	59709380
NC_010450.4	135330929	135331374	+	1.46	-732	100511027	LIN54	135331884	135398274
NC_010447.5	15135337	15135625	-	2.46	2144	100525991	LMBR1L	15121550	15137625
NC_010444.4	129809215	129809490	+	-1.71	2124	100513342	LMNB1	129807228	129862211
NC_010444.4	76120956	76121244	+	2.46	-4832	100620983	LMNB2	76125932	76146564
NC_010444.4	27042820	27043108	+	3.63	-1672	100512825	LMO2	27044636	27055457
NC_010453.5	48030480	48030730	+	-0.76	603	100523918	LMO7	48030002	48133449
NC_010444.4	103569935	103570166	+	-0.86	1684	100125826	LNPEP	103568366	103659589
NC_010446.5	91518795	91519185	+	0.61	1600	100152848	LOC100152848	91517390	91523759
NC_010449.5	23598761	23598992	+	-2.27	-409	100153163	LOC100153163	23599286	23610431
NC_010443.5	246105458	246105910	+	1.27	596	100154081	LOC100154081	246105088	246117913
NC_010443.5	221578679	221578967	+	4.05	1138	100155087	LOC100155087	221577685	221580452
NC_010443.5	246083226	246083457	+	-1.86	-2887	100155308	LOC100155308	246086229	246088291
NC_010455.5	120808694	120808982	+	0.46	4807	100155468	LOC100155468	120804031	120807604
NW_018084989.1	180936	181285	-	-1.44	656	100155553	LOC100155553	177244	181767
NC_010461.5	26309522	26309810	-	2.05	-652	100156010	LOC100156010	26308517	26309014
NC_010446.5	89736499	89737037	+	2.27	-785	100156074	LOC100156074	89737553	89751165
NC_010449.5	58624110	58624524	+	-2.73	1348	100156967	LOC100156967	58622969	58644024
NC_010449.5	58623666	58623954	+	1.05	841	100156967	LOC100156967	58622969	58644024
NC_010447.5	28235914	28236202	+	3.20	4934	100156985	LOC100156985	28231124	28231457
NC_010448.4	128007348	128007610	+	-0.95	1918	100157017	LOC100157017	128005561	128027959
NW_018085257.1	904758	904989	-	-1.12	-2540	100157267	LOC100157267	901358	902333
NC_010448.4	135054382	135054613	+	-0.44	-2484	100511460	LOC100511460	135056982	135114669
NC_010448.4	165270274	165270505	+	-2.44	2129	100511937	LOC100511937	165268260	165402668
NC_010448.4	46226628	46226916	-	2.78	2293	100512052	LOC100512052	46171184	46229065
NC_010457.5	17573773	17574061	+	1.88	-2935	100512195	LOC100512195	17576852	17578161
NW_018085237.1	409611	409842	-	-2.44	-1535	100512945	LOC100512945	407283	408191
NW_018085237.1	403466	403697	-	-1.35	4609	100512945	LOC100512945	407283	408191
NW_018084833.1	979942	980230	-	2.46	-2477	100513261	LOC100513261	965798	977609

NC_010444.4	56301740	56302025	+	-2.12	79	100513523	LOC100513523	56301803	56302714
NC_010449.5	79252144	79252375	+	-2.35	3798	100513553	LOC100513553	79248461	79249496
NC_010450.4	65877957	65878188	-	-0.44	-3584	100513671	LOC100513671	65861596	65874488
NC_010451.4	51034279	51034567	-	0.46	1450	100513751	LOC100513751	51032594	51035873
NC_010445.4	68626193	68626481	+	2.20	2226	100513982	LOC100513982	68624111	68630954
NW_018085316.1	44517	45793	-	-0.90	4256	100514433	LOC100514433	42555	49411
NW_018085316.1	46312	46861	-	-0.81	2824	100514433	LOC100514433	42555	49411
NW_018085316.1	48684	49151	-	-1.03	493	100514433	LOC100514433	42555	49411
NC_010448.4	62455693	62455981	-	1.78	720	100514469	LOC100514469	62440232	62456557
NC_010456.5	141613784	141614106	-	2.88	3592	100514734	LOC100514734	141616446	141617537
NC_010444.4	53454409	53454754	-	2.20	3251	100514828	LOC100514828	53456901	53457833
NC_010451.4	51231985	51232231	+	-2.12	993	100515044	LOC100515044	51231115	51232047
NW_018085302.1	52609	52897	-	0.88	710	100515430	LOC100515430	16214	53463
NC_010451.4	50874142	50874373	-	-0.35	-4174	100515746	LOC100515746	50869088	50870083
NC_010444.4	142952423	142953052	+	3.46	62	100515772	LOC100515772	142952675	142970336
NC_010451.4	5585787	5586075	-	1.46	3895	100516094	LOC100516094	5588354	5589826
NC_010444.4	131021001	131021232	-	-0.35	-4575	100516424	LOC100516424	130971706	131016541
NC_010461.5	61962643	61962980	+	0.05	428	100516480	LOC100516480	61962383	61969011
NC_010461.5	61962412	61962643	+	-1.71	144	100516480	LOC100516480	61962383	61969011
NC_010461.5	110623248	110623536	+	-0.12	1266	100516891	LOC100516891	110622126	110660592
NC_010451.4	5827555	5827843	-	1.78	-1422	100517357	LOC100517357	5825333	5826277
NW_018085302.1	466413	466701	-	2.46	-2203	100517394	LOC100517394	463428	464354
NC_010455.5	147507786	147508017	-	-2.44	880	100517427	LOC100517427	147485965	147508782
NC_010458.4	47796480	47796771	-	3.46	786	100517502	LOC100517502	47792699	47797412
NC_010454.4	55424979	55425210	-	-0.35	-974	100517855	LOC100517855	55406730	55424120
NW_018084852.1	94219	94758	-	4.20	1916	100518616	LOC100518616	95130	96405
NC_010444.4	54251316	54251548	+	0.14	4741	100518655	LOC100518655	54246691	54247623
NW_018085302.1	69944	70249	-	-1.86	4313	100518829	LOC100518829	73475	74410
NW_018085302.1	86183	86414	-	-2.12	1958	100519014	LOC100519014	87346	88257
NC_010444.4	64116562	64116793	-	-1.71	4912	100519297	LOC100519297	64119072	64121590
NC_010444.4	64126295	64126583	-	1.88	-4849	100519297	LOC100519297	64119072	64121590
NC_010444.4	12995769	12996089	-	-0.86	-2183	100519351	LOC100519351	12992794	12993746
NC_010443.5	236707979	236708267	-	3.46	3977	100519461	LOC100519461	236709468	236712100
NC_010451.4	51499608	51499839	-	-1.44	4300	100519506	LOC100519506	51503098	51504024

NC_010444.4	10142332	10142622	+	0.61	-193	100519643	LOC100519643	10142670	10158110
NW_018085302.1	187955	188186	-	-1.54	-572	100520237	LOC100520237	186005	187498
NC_010454.4	50156078	50156440	+	-0.12	774	100520452	LOC100520452	50155485	50171525
NW_018085302.1	169045	169276	+	-1.71	-1392	100520607	LOC100520607	170553	171917
NC_010451.4	5114757	5114988	+	-1.86	4552	100521140	LOC100521140	5110320	5116455
NC_010460.4	46047335	46047704	-	2.46	-3751	100521594	LOC100521594	45989918	46043768
NC_010444.4	55274944	55275232	-	2.20	3685	100521607	LOC100521607	55277835	55278773
NW_018085208.1	29616	29904	+	0.46	1929	100521715	LOC100521715	27831	28763
NC_010451.4	35184779	35185067	-	1.61	868	100522887	LOC100522887	35167567	35185791
NC_010447.5	17642891	17643179	-	1.88	-317	100523670	LOC100523670	17635567	17642718
NC_010451.4	51628272	51628560	-	1.68	-2394	100523744	LOC100523744	51624333	51626022
NC_010451.4	51623507	51623795	-	0.46	2371	100523744	LOC100523744	51624333	51626022
NC_010453.5	3305067	3305399	+	3.05	2339	100523747	LOC100523747	3302894	3793872
NC_010449.5	80046783	80047071	-	2.27	-4778	100524030	LOC100524030	80041087	80042149
NC_010447.5	20112905	20113241	+	1.78	441	100524142	LOC100524142	20112632	20113698
NC_010451.4	4107692	4107983	-	-2.22	3130	100524159	LOC100524159	4109979	4110968
NC_010447.5	6146501	6146901	+	1.88	471	100524254	LOC100524254	6146230	6170209
NC_010444.4	135187073	135187418	+	3.46	879	100524308	LOC100524308	135186366	135188237
NC_010452.4	44957650	44958205	-	3.34	96	100524391	LOC100524391	44885486	44958024
NC_010445.4	24227830	24228304	-	0.05	764	100524613	LOC100524613	24197743	24228831
NC_010444.4	124719450	124719681	+	0.65	1041	100524732	LOC100524732	124718524	124733892
NC_010448.4	107278634	107279138	-	2.78	3761	100525229	LOC100525229	107277789	107282647
NC_010449.5	93881952	93882267	-	3.20	485	100525232	LOC100525232	93861138	93882595
NC_010444.4	61664561	61664849	-	1.20	1123	100525329	LOC100525329	61647461	61665828
NW_018084993.1	224042	224330	+	1.88	3845	100526175	LOC100526175	220341	221780
NW_018085076.1	5963	6251	+	2.46	-2923	100620277	LOC100620277	9030	12375
NC_010454.4	32120262	32120664	+	3.05	-880	100621260	LOC100621260	32121343	32156021
NC_010455.5	199602027	199602258	+	-0.86	1374	100622246	LOC100622246	199600768	199606618
NC_010451.4	92953371	92953659	+	2.20	964	100623190	LOC100623190	92952551	92990833
NC_010444.4	54335415	54335646	+	-1.71	-3479	100623502	LOC100623502	54339010	54340093
NC_010444.4	142899099	142899387	+	0.56	-1316	100624174	LOC100624174	142900559	142907853
NC_010444.4	142903044	142903332	+	3.46	2629	100624174	LOC100624174	142900559	142907853
NC_010450.4	66745131	66745419	-	2.46	3486	100624541	LOC100624541	66727047	66748761
NC_010444.4	55812167	55812398	-	-2.57	-1310	100624683	LOC100624683	55810043	55810972

NC_010450.4	66641328	66641658	-	1.78	-856	100624700	LOC100624700	66602127	66640637
NC_010450.4	86258745	86259033	+	3.46	3861	100624892	LOC100624892	86255028	86256907
NC_010453.5	15631991	15632279	-	1.88	-3480	100625564	LOC100625564	15550458	15628655
NC_010451.4	5435063	5435303	+	-2.03	4085	100625684	LOC100625684	5431098	5434483
NC_010447.5	57777755	57778060	-	0.32	-234	100625850	LOC100625850	57777008	57777673
NC_010451.4	51174681	51174969	+	2.78	988	100625852	LOC100625852	51173837	51174796
NC_010457.5	16092934	16093462	+	0.73	-1079	100625897	LOC100625897	16094277	16154467
NC_010451.4	36619691	36619929	-	-0.54	705	100627195	LOC100627195	36554800	36620515
NC_010444.4	62629128	62629450	-	2.78	2940	100736663	LOC100736663	62630428	62632229
NC_010451.4	4701467	4702370	-	1.49	4533	100737110	LOC100737110	4705204	4706452
NC_010450.4	16900506	16900737	+	-1.35	3346	100737183	LOC100737183	16897275	17012886
NC_010444.4	56767013	56767301	+	2.05	-816	100737570	LOC100737570	56767973	56768920
NC_010448.4	50773008	50773293	+	-3.29	265	100737582	LOC100737582	50772885	50807359
NC_010444.4	8626918	8627338	-	1.46	1484	100737764	LOC100737764	8609119	8628612
NC_010444.4	28497235	28497523	+	3.46	365	100737821	LOC100737821	28497014	28818396
NC_010444.4	28497859	28498147	+	0.61	989	100737821	LOC100737821	28497014	28818396
NC_010455.5	27935677	27935965	+	2.27	-1762	100738134	LOC100738134	27937583	27946237
NC_010457.5	83295668	83296109	-	1.20	-37	100738403	LOC100738403	83292977	83295851
NC_010451.4	121511845	121512199	-	-1.71	757	100738642	LOC100738642	121499756	121512779
NC_010443.5	246066598	246067074	+	3.05	4789	100739563	LOC100739563	246062047	246063289
NC_010460.4	10333249	10333650	-	2.68	-259	102162486	LOC102162486	10268077	10333190
NC_010450.4	77971122	77971698	-	1.42	-1129	102163742	LOC102163742	77969154	77970281
NC_010453.5	6762892	6763193	+	3.05	-101	102165571	LOC102165571	6763144	6766736
NC_010453.5	6761478	6761814	+	0.14	-1498	102165571	LOC102165571	6763144	6766736
NC_010451.4	57016322	57016651	+	1.36	-294	102165987	LOC102165987	57016781	57020248
NC_010452.4	43018963	43019251	+	2.88	260	102166590	LOC102166590	43018847	43083304
NC_010448.4	50842898	50843186	+	2.05	-2875	102167273	LOC102167273	50845917	50851537
NC_010449.5	48872664	48872952	-	1.88	676	102167410	LOC102167410	48741698	48873484
NC_010448.4	170637159	170637484	-	-2.12	173	102167697	LOC102167697	170619406	170637495
NC_010451.4	104912841	104913132	-	1.68	-181	106504991	LOC106504991	104883513	104912805
NW_018085302.1	96282	96585	-	-1.73	1897	106507272	LOC106507272	97394	98331
NC_010447.5	61505109	61505518	+	1.46	1382	106507517	LOC106507517	61503931	61526167
NC_010445.4	6528561	6528849	+	1.88	2711	106508762	LOC106508762	6525994	6539895
NC_010444.4	141972397	141972696	+	2.20	1504	106509580	LOC106509580	141971042	141972967

NC_010446.5	72781828	72782298	-	2.14	2071	106510078	LOC106510078	72775536	72784134
NC_010448.4	45743897	45744218	+	0.88	215	106510528	LOC106510528	45743842	45763441
NC_010448.4	61217803	61218260	-	1.46	-1917	110255329	LOC110255329	61202808	61216114
NC_010451.4	120809780	120810086	-	2.05	682	110255567	LOC110255567	120809470	120810615
NC_010452.4	2865743	2865982	-	-2.12	1130	110255734	LOC110255734	2856170	2866993
NC_010454.4	10363009	10363345	+	2.05	823	110255979	LOC110255979	10362354	10364669
NC_010454.4	49576700	49576988	-	2.14	-4056	110256093	LOC110256093	49568837	49572788
NC_010455.5	117180470	117180758	+	2.05	1277	110256340	LOC110256340	117179337	117237380
NC_010455.5	117179836	117180067	+	-0.76	614	110256340	LOC110256340	117179337	117237380
NC_010455.5	117180123	117180411	+	2.05	930	110256340	LOC110256340	117179337	117237380
NC_010443.5	2829271	2829561	+	2.46	2550	110256592	LOC110256592	2826866	3040744
NC_010456.5	74297390	74297743	-	1.78	-2478	110256823	LOC110256823	74294341	74295088
NC_010443.5	92947892	92948124	-	-1.93	4045	110256969	LOC110256969	92951121	92952053
NC_010457.5	81937641	81937929	-	1.78	3598	110257186	LOC110257186	81935837	81941383
NC_010460.4	6633981	6634239	+	0.46	1128	110257570	LOC110257570	6632982	6719077
NW_018085022.1	62274	62562	-	1.46	-4151	110258461	LOC110258461	57374	58267
NW_018085205.1	242922	243210	+	3.05	-1964	110258782	LOC110258782	245030	245962
NW_018085257.1	1383625	1383913	-	1.88	2786	110258967	LOC110258967	1385312	1386555
NW_018085257.1	641342	641771	-	1.85	4707	110258976	LOC110258976	645284	646264
NC_010444.4	142541444	142541675	+	-1.86	4258	110259139	LOC110259139	142537301	142549469
NC_010444.4	10286115	10286643	-	1.88	3681	110259249	LOC110259249	10280973	10290060
NC_010444.4	67907142	67907373	-	-0.54	550	110259338	LOC110259338	67878961	67907808
NC_010443.5	80241203	80241434	-	-2.18	4347	110259440	LOC110259440	80223165	80245666
NC_010444.4	56082553	56082876	+	-0.54	2411	110259604	LOC110259604	56080303	56081294
NC_010444.4	57505886	57506117	+	-1.67	-780	110259612	LOC110259612	57506782	57507726
NC_010444.4	151551367	151551655	+	3.46	-1285	110259673	LOC110259673	151552796	151555861
NC_010443.5	263324324	263324612	-	2.05	-4945	110259694	LOC110259694	263318582	263319523
NC_010443.5	263564109	263564469	-	-1.86	4887	110259704	LOC110259704	263568235	263569176
NC_010444.4	61581477	61581798	+	1.20	-1653	110259754	LOC110259754	61583291	61584238
NC_010445.4	4002081	4002369	-	2.05	-2817	110260072	LOC110260072	3993681	3999408
NC_010446.5	103374908	103375145	+	-0.12	-1064	110260487	LOC110260487	103376091	103390577
NC_010447.5	21010059	21010425	+	2.46	-1648	110260575	LOC110260575	21011890	21012828
NC_010447.5	79248545	79248776	+	-1.93	4942	110260597	LOC110260597	79243718	79244681
NC_010447.5	16015824	16016112	+	1.88	-451	110260660	LOC110260660	16016419	16021676

NC_010447.5	21052617	21053004	-	2.20	-1324	110260850	LOC110260850	21049913	21051486
NC_010448.4	56524837	56525068	+	-0.86	1800	110261040	LOC110261040	56523152	56525114
NC_010448.4	75002885	75003177	-	1.05	-611	110261101	LOC110261101	75000213	75002420
NC_010448.4	57426073	57426361	+	2.46	4631	110261289	LOC110261289	57421586	57442325
NC_010448.4	57850332	57850563	-	-2.35	2962	110261301	LOC110261301	57852469	57853410
NC_010448.4	61810210	61810485	-	1.24	101	110261314	LOC110261314	61800668	61810449
NC_010448.4	56993688	56993977	+	3.78	1189	110261367	LOC110261367	56992643	56994111
NC_010449.5	79605661	79605901	-	-0.54	-3846	110261611	LOC110261611	79585433	79601935
NC_010449.5	21622017	21622568	-	1.33	353	110261661	LOC110261661	21621863	21622646
NC_010449.5	20907058	20907400	+	3.63	146	110261663	LOC110261663	20907083	20908336
NC_010449.5	20906309	20906700	-	4.58	305	110261665	LOC110261665	20906299	20906810
NC_010450.4	66870391	66870683	+	2.27	2110	110262014	LOC110262014	66868427	66874496
NC_010462.3	9174689	9174981	+	3.27	444	396706	LOC396706	9174391	9227673
NC_010458.4	23761816	23762080	-	-1.08	-1886	397451	LOC397451	23685565	23760062
NC_010449.5	20882011	20882399	-	3.27	102	595122	LOC595122	20881642	20882307
NC_010448.4	103423340	103423628	-	1.46	1306	733637	LOC733637	103413841	103424790
NC_010444.4	73266609	73266897	+	1.88	443	100511917	LONP1	73266310	73286774
NC_010443.5	251977645	251977933	-	3.46	2039	100512581	LPAR1	251811583	251979828
NC_010453.5	19219375	19219667	+	0.98	3216	100738399	LPAR6	19216305	19220913
NC_010447.5	63708449	63708737	+	2.46	919	100627653	LPCAT3	63707674	63746369
NC_010459.5	43988653	43988941	+	1.78	-4913	100170771	LPIN3	43993710	44006188
NC_010447.5	48703972	48704265	-	-1.12	2608	100626686	LRMP	48649067	48706727
NC_010457.5	75750543	75750831	-	4.05	3917	100519689	LRP2	75565299	75754604
NC_010457.5	46444606	46444844	-	0.29	933	100155710	LRP2BP	46383433	46445658
NC_010443.5	196130546	196131413	+	-2.12	1163	100157858	LRRC19	196129816	196142696
NC_010443.5	196129847	196130128	+	-1.27	171	100157858	LRRC19	196129816	196142696
NC_010448.4	165134881	165135334	+	1.14	425	100525052	LRRC41	165134682	165156915
NC_010448.4	158334723	158335011	-	1.46	3435	100621320	LRRC42	158316225	158338302
NC_010448.4	158334319	158334608	-	1.20	3838	100621320	LRRC42	158316225	158338302
NC_010451.4	6662234	6662562	+	2.20	-724	100520208	LRRC51	6663122	6673272
NC_010453.5	21126397	21126685	-	2.46	-930	100738930	LRRC63	21079430	21125611
NC_010450.4	39126228	39126516	-	2.11	-2449	100522021	LRRC66	39104727	39123923
NC_010446.5	51566204	51566435	-	-0.12	4081	100152599	LRRCC1	51525044	51570401
NC_010455.5	36885793	36886024	-	-2.12	-4322	106505617	LRTM1	36870269	36881586

NC_010458.4	65595727	65596027	-	2.88	4728	100522906	LSM11	65587502	65600605
NC_010448.4	43851929	43852330	+	2.10	4247	100511455	LSM14A	43847882	43901697
NC_010444.4	76245150	76245489	+	0.46	-1690	100515470	LSM7	76247010	76251824
NC_010448.4	44759523	44759811	+	2.20	576	100516738	LSR	44759091	44773378
NC_010447.5	87487717	87488123	+	0.78	1368	100154219	LTA4H	87486552	87518520
NC_010455.5	192357707	192357995	-	-0.54	911	106505847	LTN1	192295764	192358762
NC_010454.4	27005660	27006028	+	3.56	942	100625081	LUC7L3	27004902	27032156
NC_010443.5	209667735	209668023	-	1.10	1045	102165373	LURAP1L	209621712	209668924
NC_010461.5	94627185	94627476	+	3.05	-4159	100153362	LUZP4	94631490	94647712
NC_010446.5	1299328	1299616	-	3.05	713	106509930	LY6L	1296421	1300185
NC_010454.4	44278317	44278616	-	3.78	-191	100623359	LYRM9	44271407	44278275
NC_010459.5	32509087	32509488	+	2.68	399	100625861	LZTS3	32508888	32518524
NC_010453.5	11525147	11525530	-	-1.54	-3702	100155671	MAB21L1	11518398	11521636
NC_010459.5	22635272	22636071	+	4.37	566	102159435	MACROD2	22635105	24677199
NC_010448.4	71779284	71779572	-	3.78	-253	100736650	MAD2L2	71773680	71779175
NC_010444.4	15314013	15314301	-	2.78	1008	100514701	MADD	15271581	15315165
NC_010457.5	103578738	103578969	+	1.05	-72	100522576	MAIP1	103578926	103590719
NW_018085072.1	128649	128880	+	0.14	-1090	100511048	MAK16	129855	139558
NC_010443.5	42731256	42731590	+	-2.00	1701	396919	MAN1A1	42729722	42896290
NC_010446.5	103377578	103377924	-	2.27	-619	100154147	MAN1A2	103214929	103377132
NC_010443.5	62888005	62888293	+	1.46	460	100154996	MANEA	62887689	62945586
NC_010443.5	165458590	165458987	+	1.27	-21	106504229	MAP2K5	165458810	165705612
NC_010457.5	16915819	16916123	+	1.46	4854	100511286	MAP3K19	16911117	16976598
NC_010443.5	184402064	184402295	+	-1.12	846	100526089	MAPK1IP1L	184401333	184416383
NC_010443.5	119757539	119757890	-	-0.60	1926	396833	MAPK6	119725026	119759641
NC_010454.4	60148154	60148442	-	2.05	445	102164433	MAPK7	60144138	60148743
NC_010457.5	24503357	24503588	+	-0.35	537	100516298	MARCO	24502935	24541286
NC_010452.4	48856937	48857168	+	-1.67	1157	100514867	MASTL	48855895	48877504
NC_010458.4	60272385	60272864	-	0.46	1603	100217381	MAT2B	60255541	60274228
NC_010458.4	60270434	60270722	-	-1.71	3650	100217381	MAT2B	60255541	60274228
NC_010445.4	18043644	18043950	+	2.46	765	100622994	MAZ	18043032	18047531
NC_010461.5	108568226	108568650	-	2.10	379	100512149	MBNL3	108488224	108568817
NC_010449.5	46103538	46103900	-	1.78	706	100154824	MCM3	46084648	46104425
NC_010461.5	98729766	98730054	+	2.20	52	100511797	MCTS1	98729858	98742967

NC_010449.5	23247362	23247593	-	-1.67	261	100144453	MDC1	23229508	23247739
NC_010449.5	23246197	23246656	-	-2.86	1312	100144453	MDC1	23229508	23247739
NC_010443.5	100421757	100421988	+	-0.54	2468	100524659	ME2	100419404	100475778
NC_010454.4	22830058	22830394	+	-0.27	-179	100513621	MED1	22830405	22872420
NC_010454.4	52151832	52152120	-	1.46	390	100513494	MED11	52150535	52152366
NC_010458.4	66114925	66115213	+	2.05	202	733689	MED7	66114867	66121096
NC_010454.4	60933959	60934247	-	2.05	-104	100512681	MED9	60925161	60933999
NC_010444.4	96275261	96275612	-	-3.00	921	733590	MEF2C	96122407	96276358
NC_010452.4	46800352	46800640	-	3.05	105	100626847	MEIG1	46790501	46800601
NC_010457.5	78095289	78095520	+	-0.12	-1296	100522284	METAP1D	78096701	78182281
NC_010450.4	105052167	105052415	-	-2.35	1312	100525761	METTL14	105019539	105053603
NC_010445.4	34054265	34054496	-	-2.35	1093	100523174	METTL22	34035901	34055474
NC_010445.4	23718853	23719139	-	-0.12	1226	100628086	METTL9	23670063	23720222
NC_010449.5	50750785	50751073	-	0.88	2190	110261545	MEX3B	50747828	50753119
NC_010443.5	127741749	127742117	+	2.88	526	100157390	MFAP1	127741407	127756168
NC_010454.4	4798702	4798974	-	-1.86	1126	100622284	MFSD11	4774675	4799964
NC_010457.5	95016571	95016883	+	2.78	1446	100037960	MFSD6	95015281	95102228
NC_010460.4	7862597	7862876	-	-0.86	977	100623494	MGAM2	7767831	7863714
NC_010450.4	87389209	87389582	-	1.68	841	100622296	MGST2	87318180	87390237
NC_010456.5	75444444	75445007	-	3.22	902	100155980	MICU1	75203731	75445628
NC_010448.4	135511069	135511300	-	-0.54	1353	100625285	MIGA1	135423411	135512538
NC_010448.4	135511787	135512075	-	0.29	607	100625285	MIGA1	135423411	135512538
NC_010443.5	113257189	113257420	-	-1.93	1456	102161333	MINDY2	113171047	113258761
NC_010452.4	45979369	45980364	+	1.84	-223	100525772	MINDY3	45980090	46062700
NC_010448.4	78008917	78009168	+	0.29	-2443	100623381	MINOS1	78011486	78045919
NC_010456.5	99575146	99575389	+	-0.54	841	100152243	MINPP1	99574426	99628388
NC_010451.4	77852375	77852663	+	2.20	754	100337662	MIOS	77851765	77892417
NC_010459.5	19362041	19362387	-	2.29	793	100157552	MKKS	19341024	19363007
NC_010459.5	19362661	19362892	-	0.46	230	100157552	MKKS	19341024	19363007
NC_010448.4	164887519	164887972	+	1.05	25	100233194	MKNK1	164887720	164933526
NC_010455.5	68546326	68546614	+	1.05	-12	100626891	MKRN2	68546482	68584341
NC_010443.5	202314378	202314666	+	0.73	-2936	100524063	MLLT3	202317458	202604308
NC_010443.5	202313661	202314011	+	0.46	-3622	100524063	MLLT3	202317458	202604308
NC_010443.5	202318143	202318463	+	1.68	845	100524063	MLLT3	202317458	202604308

NC_010455.5	95023320	95023551	+	-0.54	1276	100511536	MME	95022159	95122977
NC_010451.4	33486077	33486393	-	2.46	-2605	100101475	MMP12	33473625	33483630
NC_010450.4	129172109	129172351	-	-2.71	1183	100516922	MMRN1	129113951	129173413
NC_010443.5	64460333	64460632	-	3.05	-528	100513056	MMS22L	64306078	64459954
NC_010443.5	189691662	189691894	+	-1.54	1434	100310799	MNAT1	189690344	189845924
NC_010450.4	75754500	75754788	-	1.20	1167	100517105	MND1	75689845	75755811
NC_010445.4	68853227	68853670	+	-0.80	979	100738341	MOB1A	68852469	68876706
NC_010457.5	101391712	101391949	+	-1.71	917	613127	MOBKL3	101390913	101420282
NC_010457.5	101391316	101391604	+	1.46	547	613127	MOBKL3	101390913	101420282
NW_018084979.1	1081257	1081549	-	0.88	282	100520316	MOK	1046000	1081685
NC_010448.4	10725686	10725974	-	3.78	-541	100520385	MON1B	10713721	10725289
NC_010447.5	27127191	27127877	+	-1.53	1413	100154568	MON2	27126121	27246725
NC_010461.5	110783583	110784094	-	2.46	2484	100359355	MOSPD1	110693538	110786323
NC_010454.4	52878823	52879111	+	0.98	43	733696	MPDU1	52878924	52882854
NC_010443.5	53272449	53272828	+	1.27	3702	100515980	MRAP2	53268936	53318111
NC_010455.5	79139442	79139741	+	1.46	-141	100621398	MRAS	79139733	79211139
NC_010454.4	47086883	47087171	-	2.20	366	100522640	MRM3	47077812	47087393
NC_010448.4	157736083	157736371	-	1.78	-1921	100513545	MROH7	157657492	157734306
NC_010459.5	40308098	40308444	-	1.78	-4372	100736939	MROH8	40289934	40303899
NC_010446.5	18609426	18609714	+	1.88	651	100153348	MRPL13	18608919	18671523
NC_010458.4	68277407	68277749	-	1.14	102	100739015	MRPL22	68229725	68277680
NC_010458.4	68276835	68277140	-	-1.86	692	100739015	MRPL22	68229725	68277680
NC_010454.4	26669366	26669941	-	2.46	467	100737651	MRPL27	26663884	26670121
NC_010445.4	58571386	58571674	-	1.20	3580	100517705	MRPL35	58559523	58575110
NC_010456.5	112069984	112070570	-	0.88	-51	100152229	MRPL43	112068678	112070226
NC_010454.4	23766020	23766662	-	0.14	135	100628029	MRPL45	23751921	23766476
NC_010444.4	74927279	74927567	-	2.20	773	100521888	MRPL54	74925064	74928196
NC_010443.5	191317697	191317985	-	3.78	4110	414415	MRPS11	191310547	191321951
NC_010455.5	80208017	80208248	+	-0.76	-230	100158122	MRPS22	80208363	80232107
NC_010454.4	34129771	34130167	-	2.78	-395	100511221	MRPS23	34123679	34129574
NC_010446.5	56607355	56607586	+	-0.71	-195	100152709	MRPS28	56607666	56706918
NC_010460.4	8883879	8884233	+	1.20	591	100624933	MRPS33	8883465	8894473
NC_010444.4	11192774	11193031	-	-0.35	826	100627859	MS4A12	11177406	11193729
NC_010444.4	11423449	11423754	-	1.88	-3287	397166	MS4A2	11400415	11420314

NC_010444.4	89251015	89251330	+	2.05	-4474	100512158	MSH3	89255647	89456102
NC_010455.5	77178605	77179035	-	2.20	2590	100155763	MSL2	77147823	77181410
NC_010446.5	124124583	124125025	-	0.42	-366	100156808	MTF2	124051586	124124438
NC_010451.4	27974345	27974995	-	0.27	-222	100518140	MTMR2	27864335	27974448
NC_010459.5	5505628	5505924	-	1.88	1436	100153650	MTUS1	5331668	5507212
NC_010448.4	166004683	166005017	+	3.05	1220	100513867	MUTYH	166003630	166012191
NC_010448.4	166003529	166003833	+	2.78	51	100513867	MUTYH	166003630	166012191
NC_010444.4	60275410	60275698	-	2.68	418	110259321	MVB12A	60271020	60275972
NC_010445.4	72543547	72543778	-	-1.08	1639	100625349	MXD1	72517829	72545302
NC_010444.4	80637203	80637529	+	2.05	588	100512157	MXD3	80636778	80641346
NC_010446.5	12461920	12462324	-	0.20	-1762	448810	MYC	12455141	12460360
NC_010455.5	22975543	22975999	+	1.78	-1180	396646	MYD88	22976951	22979568
NC_010455.5	29739465	29739759	-	1.16	-3996	100515755	MYL3	29730271	29735616
NC_010455.5	108436531	108436848	+	-2.03	1494	100625538	MYNN	108435195	108452699
NC_010458.4	6144839	6145127	-	2.05	502	396902	MYO10	5907111	6145485
NC_010453.5	45018736	45019024	-	3.05	129	100521289	MZT1	45000735	45019009
NC_010450.4	87691301	87691649	-	0.88	-162	100513554	NAA15	87608439	87691313
NC_010452.4	29319021	29319256	-	-3.12	3570	100517363	NAA35	29221531	29322709
NC_010447.5	21625973	21626261	+	-0.12	1401	100154321	NABP2	21624716	21631716
NC_010445.4	71513541	71513829	-	0.88	-696	100523729	NAGK	71496191	71512989
NC_010445.4	71511957	71512267	-	1.05	877	100523729	NAGK	71496191	71512989
NC_010456.5	128964086	128964317	+	-0.12	1890	100157783	NANOS1	128962311	128964207
NC_010443.5	106933629	106934202	-	0.29	-810	100513328	NARS	106911267	106933105
NC_010451.4	13011304	13011535	-	1.05	-522	100514245	NARS2	12869347	13010897
NC_010444.4	26827168	26827456	-	2.27	-252	100511365	NAT10	26786172	26827060
NC_010457.5	106515888	106516119	+	-2.35	-278	100514254	NBEAL1	106516282	106686685
NC_010454.4	19788301	19788781	-	2.05	467	100625110	NBR1	19755733	19789008
NC_010455.5	133173389	133173677	+	2.46	-3676	100156513	NCBP2	133177209	133192935
NC_010448.4	91748297	91748975	+	2.75	744	110261145	NCDN	91747892	91756812
NC_010457.5	88526878	88527109	-	-0.86	1423	100158033	NCKAP1	88425861	88528417
NC_010457.5	88531381	88531612	-	-1.12	-3079	100158033	NCKAP1	88425861	88528417
NC_010457.5	18411250	18411496	+	-1.71	280	100513689	NCKAP5	18411093	19462362
NC_010445.4	114136298	114136586	-	0.46	986	574068	NCOA1	113997218	114137428
NC_010443.5	142412999	142413287	+	3.05	455	100144475	NDN	142412688	142413665

NC_010444.4	151457603	151457942	+	3.27	-4548	110259541	NDST1	151462321	151525909
NC_010457.5	139031482	139031770	-	3.78	266	100521478	NDUFA10	138999174	139031892
NC_010459.5	22480865	22481251	+	2.05	713	100520522	NDUFAF5	22480345	22508804
NC_010455.5	139730035	139730578	-	0.24	185	100624335	NDUFB4	139724145	139730492
NC_010450.4	87695818	87696049	+	0.46	660	100037985	NDUFC1	87695273	87700024
NC_010457.5	109448820	109449051	-	-0.80	2877	100516402	NDUFS1	109413726	109451813
NC_010458.4	32874340	32874742	+	1.68	123	100516161	NDUFS4	32874418	32982661
NC_010459.5	37177672	37177960	-	2.05	-552	100620656	NECAB3	37160431	37177264
NC_010451.4	46892270	46892558	-	0.88	-850	397247	NECTIN1	46823552	46891564
NC_010447.5	86731931	86732219	-	1.46	1437	100514234	NEDD1	86693802	86733512
NC_010456.5	20142026	20142257	+	-2.35	-2522	100520589	NEK1	20144664	20332248
NC_010452.4	20968936	20969229	+	0.73	2422	100521742	NEK7	20966660	21124270
NC_010454.4	44965036	44965324	+	1.20	2085	100514006	NEK8	44963095	44975092
NC_010448.4	28667580	28667912	+	-1.71	1244	100739450	NFATC3	28666502	28806423
NC_010448.4	151228962	151229193	-	-1.12	695	100523676	NFIA	150834002	151229773
NC_010456.5	2601435	2601856	-	-0.37	1609	100153822	NFIL3	2588887	2603255
NC_010448.4	47703788	47704076	+	1.46	288	100621111	NFKBIB	47703644	47712661
NC_010455.5	157271245	157271836	-	2.49	1578	100520981	NFKBIZ	157261419	157273119
NC_010451.4	56655079	56655367	-	3.78	833	100624755	NFRKB	56616865	56656056
NC_010445.4	72982986	72983344	+	3.46	90	102165918	NFU1	72983075	73013227
NC_010458.4	27855359	27855667	+	1.68	-2595	100524404	NIM1K	27858108	27930365
NC_010454.4	20722412	20722700	-	2.05	585	100524706	NKIRAS2	20717545	20723141
NC_010445.4	41320255	41320543	-	1.78	603	100233195	NME4	41306167	41321002
NC_010444.4	140167269	140167557	-	2.78	1242	100621441	NME5	140132871	140168655
NC_010457.5	941027	941289	+	-0.95	-221	100621233	NMI	941379	966519
NC_010443.5	228140966	228141254	-	3.27	780	100518171	NMRK1	228113323	228141890
NC_010445.4	125866276	125866564	+	2.46	4998	100512777	NOL10	125861422	125956626
NC_010454.4	14035887	14036118	+	0.29	353	106504086	NOL11	14035649	14058421
NC_010459.5	36196569	36196947	-	2.20	4277	100511968	NOL4L	36070836	36201035
NC_010448.4	67395637	67395868	-	-1.35	689	100518312	NOL9	67375212	67396442
NC_010456.5	113158348	113158636	+	2.46	3873	100517974	NOLC1	113154619	113166922
NC_010457.5	105894752	105895040	+	1.05	1288	100156132	NOP58	105893608	105930458
NC_010449.5	74990474	74990762	-	2.20	1490	100154880	NOP9	74985748	74992108
NC_010448.4	54636272	54636638	-	0.24	-424	100518311	NOSIP	54618977	54636031

NC_010445.4	46378810	46379098	-	2.05	-675	100523176	NPHP1	46316864	46378279
NC_010455.5	73899850	73900138	-	1.20	1025	100521403	NPHP3	73857414	73901019
NC_010458.4	52780359	52780651	-	1.27	1266	100525313	NPM1	52767455	52781771
NC_010458.4	52780878	52781323	-	2.20	670	100525313	NPM1	52767455	52781771
NC_010456.5	112821655	112821995	-	0.88	419	100627227	NPM3	112819390	112822244
NC_010449.5	59771157	59771497	+	1.88	2703	100155307	NPTN	59768624	59840594
NC_010455.5	10755511	10755911	+	1.14	-1243	100621076	NR1D2	10756954	10791219
NC_010455.5	140404109	140404340	-	-1.44	1902	397228	NR1I2	140373571	140406127
NC_010444.4	100445908	100446466	+	1.73	-2361	100621341	NR2F1	100448548	100457510
NC_010444.4	100444610	100444902	+	1.88	-3792	100621341	NR2F1	100448548	100457510
NC_010446.5	105846484	105846715	+	-1.67	686	100739349	NRAS	105845913	105853769
NC_010449.5	19003650	19003881	+	-1.27	-4026	100154240	NRSN1	19007792	19027226
NC_010450.4	939581	939869	+	3.78	-4675	100515039	NSD2	944400	1018032
NC_010457.5	48137341	48137654	+	-0.54	1151	100520868	NSD3	48136346	48245673
NC_010457.5	48138511	48138869	+	-1.27	2344	100520868	NSD3	48136346	48245673
NC_010443.5	144474121	144474724	-	1.78	-396	100620354	NSMCE3	144469617	144474026
NC_010447.5	80621734	80622022	+	3.46	-3791	100521376	NT5DC3	80625669	80692481
NC_010445.4	39935487	39935850	+	2.46	40	100516255	NTHL1	39935628	39941718
NC_010460.4	5927841	5928129	-	-0.54	894	100517250	NUB1	5899566	5928879
NC_010451.4	66433964	66434276	-	2.46	1428	100626322	NUCKS1	66398845	66435548
NC_010444.4	109059252	109059540	-	3.78	325	100512459	NUDT12	109044946	109059721
NC_010456.5	75917609	75917859	+	1.05	-342	106504089	NUDT13	75918076	75958187
NC_010453.5	19583696	19583997	-	-0.93	718	100621855	NUDT15	19572963	19584565
NC_010448.4	29340234	29340481	+	-2.71	464	100625681	NUDT21	29339893	29359596
NC_010460.4	13777490	13777802	-	2.27	127	100523151	NUP205	13674301	13777773
NC_010446.5	95570883	95571114	+	-1.54	-2975	100157113	NUP210L	95573974	95697196
NC_010454.4	6104251	6104539	-	0.14	-558	100526070	NUP85	6072731	6103837
NC_010443.5	44185810	44186098	-	1.27	270	100154658	NUS1	44156771	44186224
NC_010443.5	44183056	44183298	-	-2.71	3047	100154658	NUS1	44156771	44186224
NC_010443.5	44184913	44185304	-	-0.71	1115	100154658	NUS1	44156771	44186224
NC_010443.5	130065576	130066235	-	2.46	-264	106509022	NUSAP1	129992797	130065641
NC_010456.5	38863131	38863419	-	1.20	4152	397570	OAS1	38851083	38867427
NC_010456.5	38846909	38847234	-	2.46	-917	595128	OAS2	38827667	38846154
NC_010443.5	130035973	130036284	+	2.68	-349	100516409	OIP5	130036478	130054058

NC_010449.5	22513015	22513303	+	3.78	-2516	100141414	OLF42-3	22515675	22516659
NC_010443.5	26803429	26803717	+	2.46	2902	100153431	OLIG3	26800671	26801489
NC_010448.4	153878240	153878813	+	2.27	-3908	100626843	OMA1	153882435	153950460
NC_010448.4	51963784	51964091	-	2.73	3686	106508274	OPA3	51917956	51967624
NC_010459.5	62906873	62907161	+	2.20	1317	397364	OPRL1	62905700	62913018
NC_010444.4	11876455	11876743	+	2.46	305	100515007	OSBP	11876294	11911660
NC_010444.4	11875559	11875886	+	2.68	-571	100515007	OSBP	11876294	11911660
NC_010448.4	109218593	109218966	-	1.27	-317	100511459	OSBPL1A	108997736	109218462
NC_010456.5	47247314	47247643	-	1.78	-642	100152038	OSM	47242767	47246836
NC_010446.5	37898035	37898456	-	2.01	-726	100155180	OSR2	37890200	37897519
NC_010448.4	78241753	78242151	+	3.05	340	110261108	OTUD3	78241612	78273544
NC_010458.4	26659180	26659468	-	1.27	415	396978	OXCT1	26514497	26659739
NC_010446.5	30931602	30931890	-	2.20	-2195	100153332	OXR1	30735844	30929551
NC_010456.5	31457343	31457591	-	0.46	2069	497623	P2RX7	31415188	31459536
NC_010456.5	75919008	75919478	-	1.46	-122	100037299	P4HA1	75827207	75919121
NC_010444.4	134535128	134535416	-	3.05	-1057	100623801	P4HA2	134502210	134534215
NC_010447.5	21496886	21497174	+	2.20	1022	100737962	PA2G4	21496008	21503297
NC_010444.4	15381909	15382197	+	2.78	-669	100512824	PACSIN3	15382722	15389893
NC_010451.4	44513261	44513603	+	2.46	318	100523683	PAFAH1B2	44513114	44534312
NC_010447.5	81386652	81386940	+	3.46	1395	100521900	PAH	81385401	81460569
NC_010459.5	31826420	31826764	-	3.46	-4396	100520054	PANK2	31797604	31822196
NC_010459.5	31826837	31827143	-	-1.35	-4794	100520054	PANK2	31797604	31822196
NC_010444.4	88329005	88329293	+	1.37	848	100512336	PAPD4	88328301	88388242
NC_010458.4	75254748	75255036	-	2.46	4913	100517864	PAPD7	75217212	75259805
NC_010449.5	117675492	117675746	+	-0.80	-464	100156323	PAPOLA	117676083	117740340
NC_010445.4	81024527	81025086	-	2.46	562	100523547	PAPOLG	80986395	81025369
NC_010455.5	83668551	83668909	-	0.88	792	100516760	PAQR9	83660387	83669522
NC_010459.5	52192016	52192304	+	1.88	-3200	100151957	PARD6B	52195360	52211504
NC_010447.5	66439447	66439678	+	-2.44	-4030	100524437	PARP11	66443593	66483630
NC_010458.4	30306937	30307225	+	1.78	1418	100511108	PARP8	30305663	30486416
NC_010448.4	150604576	150604908	-	3.27	823	100524203	PATJ	150248714	150605565
NC_010456.5	11563213	11563501	-	3.63	3540	100141310	PBK	11543657	11566897
NC_010449.5	24226701	24227239	-	2.29	-30	100144541	PBX2	24221565	24226940
NC_010449.5	24224790	24225110	-	3.78	1990	100144541	PBX2	24221565	24226940

NC_010446.5	92036972	92037506	-	0.73	3485	100038007	PCD1B	92035122	92040724
NC_010444.4	143498219	143498507	-	0.46	2841	100626940	PCDH1	143475931	143501204
NC_010444.4	142865639	142866040	+	2.27	-2301	102166091	PCDHB6	142868141	142872850
NC_010453.5	78555800	78556168	-	0.61	800	100518091	PCID2	78541278	78556784
NC_010455.5	133802073	133802361	+	0.61	1256	100512194	PCYT1A	133800961	133851004
NC_010455.5	19598054	19598416	+	-0.54	1171	100512811	PDCD6IP	19597064	19664047
NC_010447.5	52734000	52734288	-	1.88	3627	396555	PDE3A	52413503	52737771
NC_010450.4	104273187	104273573	+	-1.12	1445	397616	PDE5A	104271935	104420955
NC_010450.4	104271763	104272108	+	3.05	0	397616	PDE5A	104271935	104420955
NC_010450.4	123923904	123924192	-	2.05	3371	100514859	PDHA2	123926253	123927419
NC_010457.5	46543729	46544020	-	1.14	2708	414421	PDLIM3	46516178	46546583
NC_010459.5	35615711	35616208	-	2.88	107	100738964	PDRG1	35608805	35616067
NC_010453.5	9016098	9016386	+	3.05	1491	100154396	PDS5B	9014751	9222825
NC_010453.5	5300813	5301101	+	2.20	-2652	397288	PDX1	5303609	5309063
NC_010451.4	46401764	46402052	+	0.88	470	100519043	PDZD3	46401438	46406207
NC_010456.5	127500502	127500733	-	-0.71	1803	100153734	PDZD8	127395842	127502421
NC_010457.5	118334548	118334797	-	-1.44	1213	100312978	PECR	118296359	118335886
NC_010444.4	5959393	5959737	-	1.46	220	100519073	PELI3	5950552	5959785
NC_010454.4	39932119	39932407	+	1.78	583	100517306	PEX12	39931680	39936632
NC_010454.4	39926472	39926936	+	1.20	-4976	100517306	PEX12	39931680	39936632
NC_010446.5	59252534	59252837	+	-2.29	353	100152963	PEX2	59252332	59270117
NC_010459.5	55155810	55156163	+	0.88	214	100144532	PFDN4	55155772	55168024
NC_010456.5	22779560	22779791	-	-1.35	1228	106508765	PGAM5	22773135	22780904
NC_010461.5	62186424	62186712	+	3.46	-904	407608	PGK1	62187472	62210321
NC_010461.5	97738365	97738653	+	2.78	3107	396946	PGRMC1	97735402	97743424
NC_010449.5	9341255	9341597	+	2.37	-2505	100153737	PHACTR1	9343931	9666886
NC_010443.5	21726323	21726611	-	1.63	-3212	100524350	PHACTR2	21438744	21723255
NC_010448.4	85411618	85411946	+	2.46	-211	100622686	PHACTR4	85411993	85529441
NC_010446.5	8468880	8469168	-	1.05	-715	100153657	PHF20L1	8394874	8468309
NC_010443.5	45216284	45216515	+	-2.35	-3025	110260283	PHF3	45219425	45295074
NC_010445.4	17656390	17656703	-	1.68	217	100310801	PHKG2	17640790	17656764
NC_010443.5	158860341	158860629	-	1.46	3158	100154633	PHLPP1	158642810	158863643
NC_010444.4	340784	341225	+	2.27	-367	100512701	PHRF1	341372	371699
NC_010446.5	106808442	106808948	+	-3.71	-231	100154428	PHTF1	106808926	106885954

NC_010446.5	97917513	97917744	+	-0.80	-606	100156778	PI4KB	97918235	97949929
NC_010451.4	19945912	19946172	-	0.46	1197	100519857	PICALM	19835934	19947239
NC_010451.4	19944856	19945116	-	-0.54	2253	100519857	PICALM	19835934	19947239
NC_010451.4	19945282	19945591	-	-1.35	1802	100519857	PICALM	19835934	19947239
NC_010446.5	109031667	109031958	-	2.27	1902	102160142	PIFO	109021109	109033715
NC_010443.5	116517775	116518063	-	0.78	1732	100515767	PIGB	116492910	116519651
NC_010443.5	159318310	159318598	+	2.29	589	100522054	PIGN	159317865	159417335
NC_010456.5	48312827	48313115	-	3.46	4573	100153506	PISD	48303459	48317544
NC_010454.4	47784252	47784578	-	2.68	972	100623388	PITPNA	47739631	47785387
NC_010445.4	98266449	98266781	-	3.05	1924	100516968	PKDCC	98258456	98268539
NC_010446.5	57876418	57876706	-	2.78	2622	397408	PKIA	57872114	57879184
NC_010446.5	127708492	127708760	-	-2.67	649	100154766	PKN2	127579097	127709275
NC_010451.4	52302982	52303270	+	1.20	104	100525656	PKNOX2	52303022	52608505
NC_010451.4	128000117	128000385	+	0.17	-147	100520687	PLA2G4A	128000398	128164825
NC_010447.5	9714120	9714408	+	2.78	410	100514711	PLA2G6	9713854	9777921
NC_010443.5	196177336	196177700	+	2.56	1509	100511733	PLAA	196176009	196219614
NC_010447.5	54259793	54260047	+	-0.93	2211	397632	PLCZ1	54257709	54452557
NC_010446.5	41533965	41534196	-	0.65	851	110260255	PLEKHF2	41512109	41534932
NC_010444.4	76404957	76405245	-	0.88	69	100514627	PLEKHJ1	76402453	76405170
NC_010451.4	39950019	39950307	-	2.20	-454	396570	PLET1	39936355	39949709
NC_010443.5	203684044	203684332	+	1.78	182	397402	PLIN2	203684006	203694677
NC_010443.5	203685072	203685360	+	2.05	1210	397402	PLIN2	203684006	203694677
NC_010445.4	22548090	22548378	-	1.05	2667	396953	PLK1	22537814	22550901
NC_010446.5	118754810	118755098	-	2.63	-2455	100158146	PLPPR4	118713987	118752499
NC_010455.5	86867484	86868288	-	1.14	1683	100620055	PLSCR1	86836288	86869569
NC_010455.5	73102632	73102920	-	2.46	-3990	100624579	PLXNA1	73054434	73098786
NC_010443.5	57129792	57130163	+	-2.08	1877	106508885	PNRC1	57128100	57132071
NC_010461.5	68577760	68578016	-	0.46	2029	100523581	POF1B	68503170	68579917
NC_010451.4	8906886	8907174	+	2.46	539	100626052	POLD3	8906491	8956845
NC_010447.5	6174899	6175187	+	1.46	-3735	100525164	POLDIP3	6178778	6201940
NC_010443.5	179528894	179529182	-	0.05	853	100521651	POLE2	179495278	179529891
NC_010443.5	238193859	238194147	+	2.05	-3656	100156445	POLR1E	238197659	238219475
NC_010457.5	59479080	59479543	+	1.10	-319	100737579	POLR2D	59479631	59495975
NC_010447.5	9917218	9917608	-	0.78	308	100737214	POLR2F	9905359	9917721

NC_010456.5	6521399	6521697	+	-0.12	535	100154035	POLR3D	6521013	6529526
NC_010459.5	26719256	26719563	+	1.20	70	100738796	POLR3F	26719339	26737635
NC_010449.5	100391557	100391850	-	1.46	460	100155173	POMT2	100350451	100392164
NC_010460.4	22885304	22885535	+	-0.86	-288	100192443	POT1	22885708	22981893
NC_010456.5	73166782	73167070	-	1.61	762	100155201	PPA1	73132368	73167688
NC_010450.4	116249396	116249684	+	2.05	9	100521105	PPA2	116249531	116333847
NC_010459.5	62537499	62537787	+	2.20	133	110257398	PPDPF	62537510	62539012
NC_010450.4	71550840	71551128	-	2.46	4993	100511942	PPEF2	71519873	71555977
NC_010451.4	113730852	113731140	+	1.20	3139	100512361	PPFIA4	113727857	113777772
NC_010451.4	113727491	113727779	+	0.46	-222	100512361	PPFIA4	113727857	113777772
NC_010457.5	75942601	75942832	+	1.46	2317	100155786	PPIG	75940399	75979168
NC_010457.5	75941498	75941982	+	0.00	1341	100155786	PPIG	75940399	75979168
NC_010449.5	32549965	32550253	-	0.05	-2235	100524445	PPIL1	32522736	32547874
NC_010457.5	104583214	104583639	-	1.31	325	100156964	PPIL3	104569138	104583752
NC_010445.4	111748949	111749257	+	-1.44	1091	100739632	PPM1G	111748012	111770868
NC_010449.5	23164621	23164912	-	1.46	1104	100144450	PPP1R10	23148843	23165871
NC_010449.5	22634157	22634388	+	-1.12	49	100141423	PPP1R11	22634223	22636987
NC_010447.5	101491630	101492255	+	-2.18	1660	397190	PPP1R12A	101490282	101639456
NC_010451.4	65067202	65067490	-	0.46	1737	100512360	PPP1R15B	65056495	65069083
NC_010454.4	22689852	22690140	-	2.78	975	100736966	PPP1R1B	22681244	22690971
NC_010455.5	132487369	132488282	-	1.19	1664	100156975	PPP1R2	132460186	132489490
NC_010444.4	136519094	136519499	-	3.27	3333	397656	PPP2CA	136497780	136522630
NC_010451.4	131074369	131074731	-	0.46	1253	100101925	PPP2R5A	131003903	131075803
NC_010445.4	85652914	85653145	+	-0.93	2965	100512224	PPP4R3B	85650064	85708808
NC_010451.4	56750179	56750410	-	-2.54	-807	100517360	PRDM10	56657270	56749487
NC_010447.5	12639527	12639815	+	1.46	1281	100155716	PRDM4	12638390	12668890
NC_010450.4	103193285	103193595	+	1.46	-356	100522885	PRDM5	103193796	103383955
NC_010450.4	137615161	137615449	-	1.88	-3916	100514243	PRDM8	137604805	137611389
NC_010448.4	165844558	165844846	+	2.20	-2688	100512476	PRDX1	165847390	165859918
NC_010447.5	15045030	15045261	-	-0.86	4530	414426	PRKAG1	15033051	15049676
NC_010450.4	136756249	136756564	+	2.27	1092	100512796	PRKG2	136755314	136854589
NC_010457.5	84156435	84156764	-	3.05	-3	100155653	PRKRA	84134273	84156596
NC_010457.5	84155453	84155741	-	4.05	999	100155653	PRKRA	84134273	84156596
NC_010445.4	73707393	73707681	-	2.78	3873	100737690	PROKR1	73689583	73711410

NC_010446.5	111345745	111346135	-	2.05	1565	100154667	PRPF38B	111338988	111347505
NC_010457.5	60440166	60440540	-	1.68	125	100157937	PRPF40A	60391451	60440478
NC_010454.4	24209303	24209711	-	2.78	-775	100626612	PRR15L	24203811	24208732
NC_010444.4	77649461	77650003	+	2.46	-9	102159474	PRSS57	77649741	77657627
NC_010456.5	74769486	74769774	-	2.88	33	100153167	PSAP	74734185	74769663
NC_010459.5	12649310	12649598	+	2.46	-1229	100623956	PSD3	12650683	13267176
NC_010449.5	96480118	96480507	+	2.31	2133	780411	PSEN1	96478179	96567884
NC_010444.4	44740606	44741022	+	2.88	712	100516779	PSMA1	44740102	44755296
NC_010443.5	187342686	187342974	+	2.42	599	100154408	PSMA3	187342231	187371149
NC_010448.4	91829689	91829977	-	1.46	109	100622444	PSMB2	91792055	91829942
NC_010451.4	103362041	103362607	+	0.16	-360	100514865	PSMC2	103362684	103376048
NC_010448.4	48407413	48407701	+	3.46	1320	110260986	PSMC4	48406237	48414470
NC_010457.5	131807840	131808128	+	4.05	390	100153828	PSMD1	131807594	131912194
NC_010448.4	17046870	17047370	+	2.73	-300	100626274	PSMD7	17047420	17057170
NC_010443.5	253090985	253091713	-	2.74	1292	768100	PTBP3	252986716	253092641
NC_010458.4	48604438	48605104	+	1.34	-243	100519399	PTCD2	48605014	48635954
NC_010458.4	48601053	48601492	+	2.78	-3741	100519399	PTCD2	48605014	48635954
NC_010452.4	38938432	38938845	-	2.46	-1155	100620580	PTCHD3	38927543	38937483
NC_010456.5	99932520	99932886	+	1.46	2647	100156264	PTEN	99930056	100021619
NC_010456.5	99930470	99930701	+	0.29	529	100156264	PTEN	99930056	100021619
NC_010458.4	25674454	25674893	+	2.46	3841	100625072	PTGER4	25670832	25686556
NC_010454.4	19909200	19909431	+	-0.71	278	110255999	PTGES3L	19909037	19915852
NC_010451.4	102561794	102562039	-	-1.71	2052	100625763	PTPN12	102480914	102563969
NC_010451.4	102562365	102562846	-	0.73	1363	100625763	PTPN12	102480914	102563969
NC_010449.5	110453855	110454143	-	2.78	-179	100152076	PTPN21	110374915	110453820
NC_010449.5	110452385	110452773	-	0.14	1241	100152076	PTPN21	110374915	110453820
NC_010452.4	21484181	21484469	+	2.46	1341	100522631	PTPRC	21482984	21601627
NC_010443.5	34695465	34695803	+	-1.86	2262	100153609	PTPRK	34693372	35258699
NC_010447.5	57260193	57260491	-	1.14	3053	100524563	PTPRO	57021819	57263395
NC_010454.4	35932355	35932753	-	2.95	672	100519562	PTRH2	35924030	35933226
NC_010458.4	63109219	63109450	-	-1.76	3005	397015	PTTG1	63104698	63112340
NC_010448.4	87686212	87686500	-	2.37	1127	100517038	PUM1	87546388	87687483
NC_010451.4	53028679	53028967	-	1.78	-302	100512802	PUS3	53018878	53028521
NC_010447.5	74836332	74836573	-	-0.54	-1921	100155187	PUS7L	74805634	74834531

NC_010448.4	63593874	63594162	+	2.46	479	100524091	PUSL1	63593539	63596921
NC_010458.4	63409425	63409713	+	3.27	-329	100518878	PWWP2A	63409898	63449997
NC_010452.4	13845545	13845833	-	2.78	211	100524702	PYCR2	13841755	13845900
NC_010443.5	4880196	4880513	-	0.46	3031	492277	QKI	4732691	4883386
NC_010445.4	102823112	102823400	-	2.20	-166	397424	QPCT	102795199	102823090
NC_010455.5	31713202	31713684	-	3.78	1080	100525663	QRICH1	31663812	31714523
NC_010447.5	22688317	22688548	-	-2.71	1493	100627213	R3HDM2	22558645	22689926
NC_010445.4	112924210	112924541	-	3.05	-187	100526104	RAB10	112842190	112924188
NC_010445.4	112922951	112923245	-	1.05	1090	100526104	RAB10	112842190	112924188
NC_010445.4	112921705	112922209	-	0.10	2231	100526104	RAB10	112842190	112924188
NC_010443.5	163859248	163859650	+	2.46	1195	595117	RAB11A	163858254	163887741
NC_010444.4	70893406	70893694	-	1.20	2619	100513782	RAB11B	70884457	70896169
NC_010444.4	70895189	70895635	-	0.68	757	100513782	RAB11B	70884457	70896169
NC_010457.5	48528974	48529205	+	-2.35	-2168	100625212	RAB11FIP1	48531258	48561408
NC_010448.4	99242542	99242830	-	2.46	1941	110261168	RAB12	99216225	99244627
NC_010443.5	261255801	261256097	-	0.78	964	595112	RAB14	261228112	261256913
NC_010456.5	40140059	40140406	-	3.78	1028	100151805	RAB35	40124318	40141261
NC_010455.5	6906192	6906505	+	-2.12	1046	100144499	RAB5A	6905302	6940165
NC_010454.4	20613978	20614327	+	0.78	161	100523862	RAB5C	20613991	20635952
NC_010451.4	116960797	116961299	+	1.88	1884	100519968	RABGAP1L	116959164	117060057
NC_010452.4	24866708	24867201	-	2.05	186	100511035	RABIF	24859382	24867141
NC_010458.4	47500127	47500559	+	2.20	674	100515763	RAD17	47499669	47529703
NC_010455.5	65358954	65359242	-	0.61	408	100217383	RAD18	65261270	65359506
NC_010443.5	248450097	248450328	+	-0.35	2221	100153668	RAD23B	248447991	248493651
NC_010449.5	91592044	91592467	+	1.46	-157	100519785	RAD51B	91592413	91812676
NC_010454.4	60771027	60771443	-	2.68	-3390	110256137	RAI1	60751159	60767845
NC_010448.4	98597442	98597853	-	2.14	1297	100519155	RALBP1	98550925	98598945
NC_010456.5	24316347	24316600	-	-2.54	1550	397655	RAN	24312810	24318024
NC_010449.5	10055771	10056002	-	-0.76	1870	100153339	RANBP9	9976094	10057757
NC_010449.5	10057665	10057953	-	3.05	-52	100153339	RANBP9	9976094	10057757
NC_010454.4	53484947	53485178	+	0.65	478	100628074	RANGRF	53484584	53486716
NC_010447.5	32912792	32913059	+	-1.71	2744	100152555	RAP1B	32910181	32961972
NC_010444.4	133950313	133950552	-	-2.27	986	100521255	RAPGEF6	133708265	133951419
NC_010450.4	69829083	69829649	-	1.82	2792	100525351	RASSF6	69773526	69832158

NC_010453.5	19316283	19316515	-	-1.35	1236	100151828	RB1	19186754	19317635
NC_010456.5	38085443	38085731	+	1.20	3545	100156794	RBM19	38082042	38202548
NC_010449.5	13122360	13122684	+	-2.29	-3996	100156013	RBM24	13126518	13139504
NC_010461.5	87574465	87575034	-	0.14	368	100516772	RBM41	87512887	87575118
NC_010455.5	14973733	14973964	+	-2.12	4920	100739397	RBMS3	14968928	16458470
NC_010457.5	54724038	54724402	-	3.27	289	100514545	RBPMS	54538585	54724509
NC_010447.5	7514961	7515249	-	1.88	283	110260638	RBX1	7500492	7515388
NW_018084979.1	1332584	1332872	+	2.56	1901	100155804	RCOR1	1330827	1469644
NC_010448.4	59293751	59294086	-	2.46	-779	110261049	RDH13	59274383	59293139
NC_010448.4	59293066	59293297	-	0.88	-42	110261049	RDH13	59274383	59293139
NC_010447.5	21168612	21168900	+	2.46	1855	100517586	RDH5	21166901	21170913
NC_010456.5	66962563	66963021	+	0.65	-3276	100158143	REEP3	66966068	67071200
NC_010445.4	80910929	80911301	-	3.73	1118	100525104	REL	80861862	80912233
NC_010450.4	29185986	29186274	-	1.20	4461	100522085	RELL1	29118511	29190591
NC_010447.5	46031920	46032234	-	2.10	-514	100514348	REP15	46029302	46031563
NC_010447.5	54936859	54937147	+	1.78	-2381	100153016	RERGL	54939384	54952059
NC_010458.4	5776668	5777096	-	3.46	-7	100625174	RETREG1	5726501	5776875
NC_010445.4	59400670	59400965	+	1.68	-343	100519138	RETSAT	59401161	59416829
NC_010445.4	25239290	25239578	-	0.78	-472	100512042	REXO5	25187759	25238962
NC_010456.5	34641982	34642270	-	3.46	472	100153965	RFC5	34630374	34642598
NC_010454.4	40085815	40086103	+	1.88	-671	100627054	RFFL	40086630	40151034
NC_010451.4	118112832	118113110	-	-2.12	1297	100520856	RFWD2	117913504	118114268
NC_010443.5	115843975	115844348	+	2.46	-46	100152054	RFX7	115844208	115871951
NC_010443.5	13338486	13338717	+	-1.12	-2399	100514020	RGS17	13341001	13442058
NC_010456.5	46411183	46411471	-	1.31	-110	100158098	RHBDD3	46403146	46411217
NC_010456.5	64399720	64399951	-	0.73	47	100153330	RHOBTB1	64326461	64399883
NC_010447.5	3974048	3974400	-	-1.86	1800	100519482	RIBC2	3966339	3976024
NC_010456.5	29494927	29495215	+	2.05	111	100626388	RILPL2	29494960	29522923
NC_010451.4	105392899	105393280	+	2.29	236	100517297	RINT1	105392853	105430198
NC_010455.5	162330649	162331095	+	0.46	331	110256447	RIOX2	162330541	162354420
NC_010455.5	205152832	205153120	-	2.46	24	100518276	RIPK4	205127239	205153000
NC_010446.5	94119007	94119295	+	0.65	862	100155905	RIT1	94118289	94127882
NC_010446.5	50344900	50345259	+	1.68	190	100156087	RMDN1	50344889	50386187
NC_010444.4	80247762	80248107	-	1.88	-933	100510951	RMND5B	80226047	80247001

NC_010453.5	16911744	16912063	-	2.20	51	100625239	RNASEH2B	16825799	16911955
NC_010443.5	2146060	2146348	+	1.78	-503	100157985	RNASET2	2146707	2164768
NC_010445.4	58162136	58162882	+	-2.56	1422	100515888	RNF103	58161087	58183750
NC_010448.4	160910478	160911107	-	1.84	920	100736575	RNF11	160875868	160911713
NC_010455.5	90033992	90034576	+	0.34	-300	100621829	RNF13	90034584	90178421
NC_010444.4	48985222	48985510	+	0.20	-175	100620525	RNF141	48985541	49028891
NC_010456.5	47947500	47947788	+	3.05	1260	100157569	RNF185	47946384	47979702
NC_010449.5	75831772	75832325	-	3.14	-593	100516275	RNF212B	75798895	75831455
NC_010456.5	47360034	47360322	-	2.46	-2987	100739143	RNF215	47351296	47357191
NC_010443.5	37909780	37910201	-	1.36	2131	100738102	RNF217	37784359	37912122
NC_010454.4	34694583	34694871	-	0.88	294	100517305	RNF43	34631574	34695021
NC_010444.4	81379421	81379709	+	2.78	610	100523538	RNF44	81378955	81390227
NC_010443.5	57062126	57062467	-	2.05	-311	100520874	RNGTT	56852864	57061985
NC_010456.5	100604100	100604388	-	1.78	648	100155046	RNLS	100312592	100604892
NC_010448.4	96362008	96362239	-	-0.54	690	100621823	RNMT	96338283	96362814
NC_010445.4	125354215	125354661	+	2.05	1319	397445	ROCK2	125353119	125493368
NC_010443.5	227575148	227575379	+	0.20	1291	100739111	RORB	227573972	227776806
NC_010443.5	44410986	44411381	+	1.53	1305	100156931	ROS1	44409878	44535784
NC_010443.5	44412101	44412473	+	2.22	2409	100156931	ROS1	44409878	44535784
NC_010447.5	78065985	78066273	-	1.46	102	100623899	RPAP3	78023796	78066231
NC_010443.5	99047676	99048229	-	-0.97	424	100625001	RPL17	99044511	99048377
NC_010443.5	179476578	179476866	-	3.05	1098	396952	RPL36AL	179476638	179477820
NC_010455.5	71891909	71892140	+	-1.12	-575	397606	RPN1	71892600	71908772
NC_010455.5	71891131	71891419	+	0.78	-1325	397606	RPN1	71892600	71908772
NC_010456.5	102566364	102566652	+	1.95	706	100156540	RPP30	102565802	102606046
NC_010446.5	98734596	98734897	-	2.24	-140	100620276	RPRD2	98640925	98734606
NC_010448.4	50010328	50010616	-	0.88	-444	100518254	RPS19	50003198	50010028
NC_010443.5	203445998	203446254	+	-2.35	1675	100038023	RPS6	203444451	203447643
NC_010443.5	203443367	203443736	+	0.85	-899	100038023	RPS6	203444451	203447643
NC_010445.4	7572344	7572791	-	-1.73	-577	100624003	RRN3	7537131	7571990
NC_010446.5	93362778	93363066	-	1.46	3102	100152334	RRNAD1	93358141	93366024
NC_010451.4	12274131	12274419	-	0.68	3845	100217384	RSF1	12121824	12278120
NC_010443.5	116612105	116612698	+	2.78	66	100623328	RSL24D1	116612335	116629161
NC_010443.5	36070305	36070578	-	-2.03	-4009	100155208	RSPO3	35985985	36066432

NC_010452.4	45079792	45080159	+	-0.12	961	100524759	RSU1	45079014	45289423
NC_010446.5	117874600	117874888	-	2.05	886	100154102	RTCA	117852776	117875630
NC_010447.5	12579756	12580100	+	-0.44	-365	733658	RTCB	12580293	12605722
NC_010443.5	129941929	129942217	-	0.88	772	100524183	RTF1	129886798	129942845
NC_010445.4	68599454	68599742	+	1.61	-541	100518548	RTKN	68600139	68613633
NC_010445.4	86178097	86178328	+	-2.86	-1809	100170118	RTN4	86180022	86252126
NC_010444.4	79020722	79020953	-	0.05	-3655	100519354	RUFY1	78974361	79017182
NC_010456.5	71550090	71550464	-	1.88	-2540	100154292	RUFY2	71497232	71547737
NC_010448.4	54250892	54251233	+	2.05	556	100511637	RUVBL2	54250506	54263492
NC_010446.5	97260930	97261744	+	2.01	-497	100515138	S100A10	97261834	97272517
NC_010446.5	97235809	97236097	+	1.78	2120	445534	S100A11	97233833	97239081
NC_010454.4	26315316	26315605	-	3.27	87	102159512	SAMD14	26295159	26315548
NC_010449.5	100451378	100451609	+	-0.67	1935	100152746	SAMD15	100449558	100463889
NC_010456.5	77680300	77680611	+	-0.71	1168	100151793	SAMD8	77679287	77737657
NC_010456.5	16680950	16681299	-	1.78	799	100620430	SAP30	16674835	16681924
NC_010443.5	180432462	180432757	-	-0.41	1848	100156165	SAV1	180404871	180434458
NC_010444.4	49241109	49241397	+	2.05	-1488	100521123	SBF2	49242741	49691769
NC_010444.4	49243462	49243693	+	0.20	836	100521123	SBF2	49242741	49691769
NC_010448.4	54686433	54686721	+	1.61	1240	100521729	SCAF1	54685337	54700400
NC_010448.4	54686796	54687084	+	1.46	1603	100521729	SCAF1	54685337	54700400
NC_010447.5	76875596	76875974	-	2.05	866	100525109	SCAF11	76809441	76876651
NC_010449.5	58673279	58673567	+	0.20	784	100154517	SCAMP2	58672639	58699145
NC_010452.4	15263657	15263945	-	0.46	615	100512485	SCCPDH	15205644	15264416
NC_010449.5	68851345	68851647	-	2.14	-441	100157840	SCFD1	68742126	68851055
NC_010449.5	68850978	68851294	-	1.30	-81	100157840	SCFD1	68742126	68851055
NC_010444.4	16997	17664	+	3.18	-221	100517148	SCGB1C1	17552	20717
NC_010444.4	149119243	149119531	+	1.68	-1265	100622733	SCGB3A2	149120652	149124614
NC_010457.5	72916369	72916657	-	1.20	-4161	100516701	SCN9A	72745706	72912352
NC_010446.5	825713	826001	+	2.78	932	110260417	SCRIB	824925	852360
NC_010459.5	34561389	34561677	+	1.78	3286	100620262	SCRT2	34558247	34570434
NC_010448.4	87537239	87537745	-	2.14	-1803	100516859	SDC3	87495041	87535689
NC_010452.4	13897071	13897395	-	1.68	614	100524888	SDE2	13879482	13897847
NC_010448.4	75676967	75677271	-	1.05	992	414412	SDHB	75648362	75678111
NC_010444.4	136885270	136885512	+	0.46	1516	100511986	SEC24A	136883875	136952181

NC_010444.4	136884763	136885051	+	3.20	1032	100511986	SEC24A	136883875	136952181
NC_010450.4	112762665	112763077	-	1.68	-1	100622653	SEC24B	112670529	112762870
NC_010450.4	104560806	104561094	+	0.20	227	100739491	SEC24D	104560723	105005400
NC_010450.4	135434868	135435099	+	-0.67	2866	100511210	SEC31A	135432117	135495124
NC_010443.5	74077494	74077759	-	-1.35	978	100152304	SEC63	74003428	74078605
NC_010458.4	27546800	27547096	-	-2.58	915	100037964	SELENOP	27537885	27547863
NC_010449.5	55766716	55767004	+	4.05	1215	110261555	SEMA4B	55765645	55793982
NC_010455.5	133268293	133268581	-	3.46	763	100526077	SENP5	133196061	133269200
NC_010448.4	145188448	145188819	+	1.14	2082	100518613	SERBP1	145186551	145201781
NC_010443.5	40098767	40099492	+	2.63	-193	102158514	SERINC1	40099323	40141552
NC_010455.5	106560210	106560501	+	2.82	1029	100154352	SERPINI1	106559326	106636930
NC_010448.4	85327504	85327792	+	2.78	4325	100620966	SESN2	85323323	85348044
NC_010451.4	27277696	27278041	-	0.53	1336	100526065	SESN3	27207239	27279205
NC_010455.5	199588341	199588629	-	1.46	1439	100622154	SETD4	199564048	199589924
NC_010450.4	87479383	87479671	+	2.46	629	100626704	SETD7	87478898	87527190
NC_010456.5	129075010	129075429	-	1.46	578	100154966	SFXN4	129047831	129075798
NC_010458.4	67484171	67484463	-	-2.71	-2608	100240724	SGCD	66447995	67481709
NC_010451.4	74471077	74471509	-	-0.86	2609	100240725	SGCE	74402714	74473902
NC_010455.5	7153605	7153905	-	-0.12	-553	100520101	SGO1	7137891	7153202
NC_010444.4	72393176	72393613	+	2.20	-906	100521544	SH2D3A	72394301	72406242
NC_010445.4	59322796	59323084	-	0.46	4118	100520186	SH2D6	59319980	59327058
NC_010455.5	203046049	203046369	+	2.88	-366	100626262	SH3BGR	203046575	203108638
NC_010455.5	203046875	203047167	+	0.46	446	100626262	SH3BGR	203046575	203108638
NC_010447.5	10187815	10188117	-	1.68	-83	100623659	SH3BP1	10172303	10187883
NC_010448.4	48809067	48809355	+	3.78	-3952	110260987	SHKBP1	48813163	48826461
NC_010447.5	22539414	22539702	+	1.20	3778	100626911	SHMT2	22535780	22540322
NC_010446.5	105779529	105779760	+	-0.86	-151	100156436	SIKE1	105779796	105791241
NC_010459.5	33612382	33612670	-	3.46	-1151	494566	SIRPA	33566270	33611375
NC_010444.4	53791	54022	-	-1.86	1122	100125971	SIRT3	38465	55029
NW_018084979.1	3028127	3028415	+	1.78	-1511	110258343	SIVA1	3029782	3034647
NC_010448.4	52100528	52100816	-	2.46	-1214	100626906	SIX5	52095332	52099458
NC_010444.4	136474240	136474599	-	0.82	1944	110259483	SKP1	136458772	136476364
NC_010444.4	136474875	136475106	-	0.46	1373	110259483	SKP1	136458772	136476364
NC_010450.4	82114815	82115086	+	-0.44	-107	100192449	SLC10A7	82115058	82164221

NC_010449.5	80192295	80192572	+	0.05	2057	100156993	SLC12A6	80190376	80266330
NC_010444.4	10797632	10797975	+	2.27	-2123	100513135	SLC15A3	10799927	10814363
NC_010449.5	20487614	20487869	+	-1.95	-2551	106504136	SLC17A4	20490293	20521425
NC_010456.5	4276294	4276628	-	2.20	2876	100154435	SLC18A1	4243564	4279337
NC_010443.5	30923929	30924226	+	-0.71	360	100155211	SLC18B1	30923717	30956898
NC_010444.4	7567486	7567846	-	1.88	-1866	100520540	SLC22A12	7557822	7565800
NC_010449.5	2131036	2131324	-	2.78	598	100152838	SLC22A23	1988690	2131778
NC_010449.5	38329662	38329950	+	2.05	1332	733693	SLC22A7	38328474	38334879
NC_010443.5	163626885	163627136	+	-0.35	1174	102160209	SLC24A1	163625836	163662071
NC_010443.5	203058082	203058329	+	-1.54	-1357	100625506	SLC24A2	203059563	203336442
NC_010454.4	51971259	51971547	+	2.20	609	397390	SLC25A11	51970794	51973668
NC_010447.5	85291156	85291517	-	-1.18	999	100302698	SLC25A3	85284405	85292336
NC_010455.5	81751880	81752307	+	-0.44	1581	100513238	SLC25A36	81750512	81791869
NC_010444.4	115530186	115530553	+	2.68	707	100516066	SLC25A46	115529662	115555441
NC_010444.4	115529115	115529408	+	1.40	-400	100516066	SLC25A46	115529662	115555441
NC_010445.4	107422589	107422987	-	1.61	3542	100170144	SLC30A6	107387876	107426330
NC_010460.4	14881373	14882004	+	1.00	1366	100525844	SLC35B4	14880322	14916406
NC_010443.5	186954400	186954701	-	1.46	-2258	100152395	SLC35F4	186687797	186952292
NC_010445.4	112296445	112296733	-	1.46	224	100513653	SLC35F6	112280654	112296813
NC_010457.5	99656167	99656455	+	0.88	1321	110257100	SLC39A10	99654990	99724619
NC_010457.5	99656583	99656871	+	1.46	1737	110257100	SLC39A10	99654990	99724619
NC_010450.4	118604442	118604673	+	-2.76	-294	100524695	SLC39A8	118604852	118678113
NC_010454.4	47838722	47839139	-	1.53	196	100623009	SLC43A2	47791911	47839127
NC_010454.4	47839192	47839691	-	2.63	-314	100623009	SLC43A2	47791911	47839127
NC_010443.5	246544765	246545053	+	0.63	-3552	100152140	SLC44A1	246548461	246720968
NC_010443.5	163081295	163081738	+	2.27	371	100525144	SLC51B	163081145	163089491
NW_018084874.1	551441	551753	-	2.88	4661	100126275	SLC5A11	476405	556258
NC_010447.5	82976522	82976810	+	2.05	3487	100524807	SLC5A8	82973179	83088542
NC_010447.5	67575982	67576270	-	3.05	-2268	100512716	SLC6A12	67550286	67573858
NC_010448.4	28837484	28837790	+	1.46	2840	110260947	SLC7A6	28834797	28876154
NC_010445.4	51745652	51745940	-	1.88	4200	396786	SLC9A4	51686001	51749996
NC_010456.5	114899721	114900009	+	2.46	902	100156642	SLK	114898963	114957494
NC_010455.5	39574841	39575110	+	-1.39	3045	100514321	SLMAP	39571930	39720229
NC_010458.4	63121190	63121483	+	0.65	2910	100517791	SLU7	63118426	63145655

NC_010443.5	97509854	97510085	-	-0.12	1392	100155304	SMAD2	97415360	97511362
NC_010443.5	100576028	100576316	+	1.20	1592	397142	SMAD4	100574580	100633501
NC_010443.5	164656057	164656571	+	0.68	3712	100152069	SMAD6	164652602	164735084
NC_010443.5	98545111	98545343	-	-1.12	-1305	100521305	SMAD7	98512800	98543922
NC_010443.5	219814677	219815151	-	0.14	-1546	100157302	SMARCA2	219624820	219813368
NC_010443.5	219815337	219815652	-	1.78	-2126	100157302	SMARCA2	219624820	219813368
NC_010450.4	84222532	84222861	-	1.56	2246	100188903	SMARCA5	84184572	84224943
NC_010450.4	125484538	125484925	-	1.46	3561	100515330	SMARCAD1	125410221	125488293
NC_010457.5	118628310	118628705	+	1.07	773	100620418	SMARCAL1	118627734	118688005
NC_010447.5	6570486	6570898	-	1.73	678	100737924	SMDT1	6568430	6571370
NC_010459.5	11549634	11550022	+	1.85	-109	100152445	SMIM19	11549937	11566898
NC_010458.4	47776406	47776930	-	3.41	711	100170853	SMN1	47738776	47777379
NC_010451.4	3328190	3328602	-	1.20	97	100518980	SMPD1	3324091	3328493
NC_010454.4	14367852	14368140	+	1.29	-687	100627676	SMURF2	14368683	14492129
NC_010454.4	14369453	14369741	+	3.05	914	100627676	SMURF2	14368683	14492129
NC_010445.4	46930217	46930477	+	-2.12	1178	100739577	SNRNP200	46929169	46960420
NC_010443.5	145708315	145708603	+	0.46	1066	100156573	SNRPA1	145707393	145719287
NC_010448.4	106802459	106802747	+	1.88	4402	100524872	SNRPD1	106798201	106810178
NC_010445.4	72199808	72200039	+	-1.12	1201	100525867	SNRPG	72198722	72208053
NC_010448.4	17773143	17773599	-	0.14	1179	100620497	SNTB2	17674587	17774550
NC_010443.5	54546022	54546358	-	3.27	1014	100156800	SNX14	54460645	54547204
NC_010443.5	54547771	54548125	-	3.05	-744	100156800	SNX14	54460645	54547204
NC_010443.5	74347988	74348338	-	0.20	1572	100627262	SNX3	74306450	74349735
NC_010443.5	152635445	152635765	-	2.46	1040	100155619	SOCS6	152593647	152636645
NC_010454.4	23723003	23723340	-	1.68	253	100514187	SOCS7	23687115	23723425
NC_010443.5	180010947	180011235	-	2.78	3850	100156602	SOS2	179907578	180014941
NC_010445.4	46549500	46549738	+	-1.18	2393	110260118	SOWAHC	46547226	46552659
NC_010457.5	79982036	79982354	-	1.68	4119	100153372	SP3	79935986	79986314
NC_010457.5	76899641	76899929	+	1.29	-353	100621662	SP5	76900138	76905024
NC_010454.4	27370954	27371242	-	1.78	-834	100518147	SPAG9	27225377	27370264
NC_010445.4	107507174	107507405	-	-2.03	1625	396584	SPAST	107439810	107508915
NC_010454.4	49610783	49611071	-	1.24	-1695	100415924	SPATA22	49573050	49609232
NC_010443.5	217300327	217300558	+	-1.71	-1595	100522592	SPATA6L	217302038	217353093
NC_010449.5	110307920	110308221	+	2.78	-1344	100153278	SPATA7	110309415	110368370

NC_010456.5	49333636	49333924	+	2.46	530	100519740	SPECC1L	49333250	49463965
NC_010448.4	75016504	75016792	+	0.29	1083	100620266	SPEN	75015565	75107468
NC_010443.5	126888296	126888527	+	-1.12	-2058	100522412	SPG11	126890470	126998014
NC_010448.4	438838	439126	-	0.29	224	100627469	SPG7	414930	439206
NC_010448.4	53987105	53987393	+	2.05	4338	100738292	SPHK2	53982911	53986779
NC_010457.5	13207616	13208091	-	3.85	-186	100156406	SPOPL	13134221	13207667
NC_010450.4	100860116	100860387	-	-0.44	3526	100623283	SPRY1	100854941	100863778
NC_010453.5	52155970	52156292	-	-0.80	2635	100157266	SPRY2	52153695	52158766
NC_010444.4	143886468	143886894	-	1.14	4141	100512891	SPRY4	143877334	143890822
NC_010447.5	18338921	18339180	-	-1.61	-359	100514838	SPRYD3	18321006	18338691
NC_010453.5	17849814	17850045	+	-0.86	918	100155063	SPRYD7	17849011	17870090
NC_010449.5	100637768	100638071	-	2.27	1053	100158010	SPTLC2	100545360	100638973
NC_010449.5	100639115	100639605	-	-0.35	-387	100158010	SPTLC2	100545360	100638973
NC_010449.5	100637367	100637715	-	-1.12	1432	100158010	SPTLC2	100545360	100638973
NC_010449.5	65303625	65303985	+	0.65	918	100156815	SPTSSA	65302887	65325877
NC_010455.5	101057990	101058267	-	-0.35	432	100623291	SPTSSB	101027944	101058561
NC_010443.5	126118384	126118805	-	3.46	976	100154990	SQOR	126078340	126119571
NC_010458.4	43345775	43346262	-	1.29	233	100620947	SREK1IP1	43297364	43346252
NC_010455.5	65758268	65758556	-	1.37	1480	102167373	SRGAP3	65367596	65759892
NC_010450.4	55724566	55724854	+	3.05	-4492	100520920	SRP72	55729202	55762298
NC_010450.4	55728119	55728454	+	2.24	-915	100520920	SRP72	55729202	55762298
NC_010445.4	101597105	101597397	+	0.88	-2178	100518189	SRSF7	101599429	101608786
NC_010455.5	26109808	26110039	+	-0.54	1636	100523140	SS18L2	26108287	26112625
NC_010454.4	46089752	46090040	-	2.78	-4176	100520864	SSH2	45818103	46085720
NC_010444.4	13619950	13620238	+	3.46	428	100624907	SSRP1	13619666	13629233
NC_010445.4	58861974	58862205	+	1.73	-905	100625751	ST3GAL5	58862995	58919544
NC_010447.5	51209012	51209345	+	2.05	-2133	100625270	ST8SIA1	51211312	51364226
NC_010452.4	43947644	43947949	+	-1.71	806	100523271	STAM	43946990	44016064
NC_010457.5	118616	118904	+	3.78	3462	100157042	STAM2	115298	168659
NC_010451.4	108631544	108631942	+	0.88	504	100529117	STARD3NL	108631239	108675617
NC_010445.4	46993414	46993702	+	1.73	850	100525865	STARD7	46992708	47017668
NC_010445.4	46992123	46992488	+	2.78	-402	100525865	STARD7	46992708	47017668
NC_010448.4	164199358	164199589	+	-0.44	1947	100522443	STIL	164197526	164266615
NC_010448.4	164197271	164197884	+	2.60	51	100522443	STIL	164197526	164266615

NC_010457.5	121604004	121604329	+	2.68	91	110256991	STK11IP	121604075	121619066
NC_010446.5	38012447	38012678	+	-2.35	760	100524741	STK3	38011802	38306777
NC_010448.4	92482851	92483407	-	2.05	1234	100625215	STK40	92441231	92484363
NC_010457.5	105056941	105057229	+	2.20	1838	100511661	STRADB	105055247	105083429
NC_010451.4	52726264	52726515	+	0.24	-404	100511525	STT3A	52726794	52754671
NC_010455.5	17535549	17535837	+	-1.08	3352	100526009	STT3B	17532341	17635039
NC_010444.4	11740399	11740630	-	-0.27	-668	100520886	STX3	11694791	11739846
NC_010446.5	111293695	111293983	-	1.05	228	100519481	STXBP3	111234154	111294067
NC_010443.5	18511580	18511811	-	-2.12	1054	100152912	STXBP5	18343826	18512750
NC_010443.5	182358058	182358488	+	0.88	676	100517509	STYX	182357597	182407790
NC_010445.4	60142430	60142782	+	1.68	197	399539	SUCLG1	60142409	60177497
NC_010455.5	49090280	49090579	-	1.46	91	397026	SUCLG2	48824366	49090521
NC_010455.5	92114425	92114656	+	1.46	-2269	100739158	SUCNR1	92116810	92130683
NC_010444.4	58574579	58574886	+	1.46	298	110259310	SUGP1	58574434	58615054
NC_010453.5	12865119	12865532	-	2.78	-1143	100739486	SUPT20H	12825932	12864182
NC_010443.5	272987933	272988221	-	3.78	606	100623620	SURF4	272975164	272988683
NC_010446.5	130701119	130701407	+	2.20	-1077	100154622	SYDE2	130702340	130749742
NC_010443.5	54597274	54597562	-	0.46	157	100154370	SYNCRIP	54563112	54597575
NC_010455.5	196329330	196329618	-	3.37	1037	100525664	SYNJ1	196230101	196330511
NC_010454.4	39070135	39070440	-	2.05	-445	100624012	SYNRG	38974567	39069842
NC_010451.4	105981843	105982074	-	-0.93	3213	100518030	SYPL1	105952898	105985172
NC_010459.5	47686711	47686999	+	1.88	671	102162899	SYS1	47686184	47694045
NC_010443.5	16615606	16615894	-	0.20	-3871	100156182	TAB2	16533364	16611879
NC_010451.4	77196189	77196420	+	1.24	-1104	100525179	TAC1	77197409	77206529
NC_010450.4	117661443	117661731	+	0.88	-535	100521983	TACR3	117662122	117718317
NC_010451.4	3144127	3144724	+	2.27	163	414401	TAF10	3144262	3145702
NC_010452.4	63573760	63574012	-	-0.12	2514	100620508	TAF3	63422184	63576400
NC_010456.5	114304114	114304379	+	2.14	-316	100152210	TAF5	114304563	114321624
NC_010444.4	9003397	9003685	-	1.20	-3551	100620136	TAF6L	8983823	8999990
NC_010446.5	90566767	90567055	+	1.88	639	100515893	TAGLN2	90566272	90574762
NC_010443.5	246929523	246929811	+	3.46	-3308	100154895	TAL2	246932975	246944722
NC_010454.4	45598184	45598415	+	-0.27	1232	100622172	TAOK1	45597067	45750117
NC_010456.5	34329484	34329772	+	4.05	-1634	100154763	TAOK3	34331262	34514234
NC_010449.5	29672368	29672656	-	2.78	941	100155428	TAPBP	29663491	29673453

NC_010458.4	19454400	19454995	+	2.20	-596	100526191	TARS	19455294	19486735
NC_010460.4	44906919	44907207	-	2.20	3202	100516165	TAX1BP1	44827908	44910265
NC_010455.5	4606010	4606257	-	-1.35	2549	100626058	TBC1D5	4191544	4608683
NC_010447.5	28881072	28881674	+	-0.98	1163	100125828	TBK1	28880210	28929964
NC_010455.5	115486039	115486326	-	-0.37	932	100522831	TBL1XR1	115312445	115487115
NC_010455.5	115486796	115487059	-	-0.18	187	100522831	TBL1XR1	115312445	115487115
NC_010445.4	39995712	39996000	-	1.46	-257	100623969	TBL3	39989739	39995599
NC_010460.4	9886455	9886743	-	3.05	100	397112	TBXAS1	9717291	9886699
NC_010455.5	27647953	27648305	+	1.88	59	100627345	TCAIM	27648070	27703489
NC_010444.4	147790141	147790530	+	2.73	1110	100621703	TCERG1	147789225	147852461
NC_010456.5	47496428	47496716	+	2.20	318	100152846	TCN2	47496254	47515966
NC_010444.4	27903245	27903476	-	-1.08	-194	100621866	TCP11L1	27854394	27903166
NC_010449.5	111769451	111769739	+	2.20	2044	100153247	TDP1	111767551	111815292
NC_010456.5	124524622	124524901	+	-1.54	-1251	100157555	TDRD1	124526013	124575166
NC_010446.5	62833423	62833711	-	2.05	-2108	100516798	TERF1	62797244	62831459
NC_010460.4	29934696	29935116	-	-1.56	1342	100125376	TES	29895878	29936248
NC_010454.4	714875	715106	+	-0.93	1811	100624315	TEX19	713179	715704
NC_010455.5	33893513	33893864	+	-0.35	410	102161929	TEX264	33893278	33924175
NC_010455.5	74864142	74864407	+	-1.93	1772	396845	TF	74862502	74907059
NC_010449.5	44641911	44642203	+	4.05	1776	100155785	TFAP2D	44640281	44702561
NC_010443.5	128194239	128194506	+	-0.12	2725	100158178	TGM5	128191647	128217249
NC_010445.4	96872364	96872652	+	2.05	330	100516259	THADA	96872178	97197051
NC_010459.5	11771897	11772216	-	1.46	1264	100621925	THAP1	11765383	11773321
NC_010448.4	28465723	28466190	+	3.27	498	100520959	THAP11	28465458	28466387
NC_010447.5	35671794	35672025	+	-1.27	629	100152778	THAP2	35671280	35687298
NC_010456.5	50594723	50595011	-	3.46	-1089	100526013	THAP7	50591412	50593778
NC_010458.4	65609887	65610175	-	2.46	588	100521164	THG1L	65602637	65610619
NC_010458.4	65610243	65610670	-	1.46	162	100521164	THG1L	65602637	65610619
NC_010445.4	100556581	100556869	+	0.88	-443	100517336	THUMPD2	100557168	100597664
NC_010456.5	129467704	129468021	-	3.46	-790	100158186	TIAL1	129441210	129467072
NC_010450.4	110378281	110378569	+	1.88	608	100513485	TIFA	110377817	110394190
NC_010444.4	76147080	76147368	+	1.05	278	100620885	TIMM13	76146946	76148163
NC_010455.5	140757813	140758260	-	1.46	661	110256419	TIMMDC1	140728559	140758698
NC_010443.5	164369168	164369399	-	-0.86	-24	100153268	TIPIN	164349196	164369259

NC_010446.5	82794016	82794620	-	3.46	84	100155988	TIPRL	82759309	82794402
NC_010454.4	47914529	47914817	-	2.46	3582	110256072	TLCD2	47904150	47918255
NC_010443.5	236452747	236452978	-	-1.12	-1884	100157220	TLN1	236416041	236450978
NC_010457.5	47601469	47602023	+	0.88	-194	100154457	TM2D2	47601940	47611802
NC_010455.5	89703966	89704350	+	0.32	2987	100522400	TM4SF4	89701171	89730488
NC_010447.5	15675277	15675572	+	-1.12	1217	396907	TMBIM6	15674207	15692765
NC_010446.5	84966499	84966730	+	-1.12	4702	100151809	TMCO1	84961912	85011139
NC_010444.4	69578789	69579077	-	3.37	-2512	100627642	TMED1	69573128	69576421
NC_010456.5	29397887	29398118	-	0.88	2451	100625917	TMED2	29390903	29400454
NC_010443.5	242214143	242214464	+	2.46	-651	102166244	TMEFF1	242214955	242309878
NC_010454.4	19755038	19755423	-	3.05	291	100624959	TMEM106A	19747352	19755522
NC_010444.4	10836780	10837088	-	1.46	190	100511116	TMEM109	10828644	10837124
NC_010451.4	19544826	19545114	+	0.46	-2694	100626990	TMEM126B	19547664	19555247
NC_010460.4	6973648	6973880	-	-2.35	364	100521303	TMEM139	6970887	6974128
NC_010450.4	54753502	54753844	+	1.46	2103	100523623	TMEM165	54751570	54796352
NC_010447.5	35691629	35691917	+	2.46	-224	100519775	TMEM19	35691997	35717916
NC_010447.5	35693014	35693388	+	3.46	1204	100519775	TMEM19	35691997	35717916
NC_010450.4	43960160	43960448	+	2.46	-404	100512847	TMEM192	43960708	43994286
NC_010448.4	86037192	86037423	-	-2.71	-3960	100515438	TMEM200B	86027778	86033347
NC_010448.4	69973877	69974165	+	3.05	643	100517949	TMEM201	69973378	69998548
NC_010460.4	18615942	18616173	+	-0.12	799	100516476	TMEM209	18615258	18644708
NC_010460.4	23526526	23526757	+	-0.86	-4856	100524656	TMEM229A	23531498	23533619
NC_010459.5	14135412	14135643	-	-1.35	878	100625411	TMEM230	14125728	14136406
NC_010444.4	115523726	115524014	-	2.05	-3717	100515882	TMEM232	115387770	115520153
NC_010447.5	13396105	13396499	-	1.73	1110	100620181	TMEM263	13379490	13397412
NC_010447.5	13397798	13398086	-	2.78	-530	100620181	TMEM263	13379490	13397412
NC_010443.5	90634441	90634672	+	-1.54	-4714	100154255	TMEM30A	90639271	90688004
NC_010443.5	246973737	246974029	+	0.20	436	100153669	TMEM38B	246973447	247024019
NC_010455.5	140798003	140798294	+	0.56	1050	100151958	TMEM39A	140797098	140830732
NC_010451.4	224678	225287	+	1.11	1005	100519436	TMEM41B	223977	249813
NC_010448.4	82860995	82861283	+	2.20	-654	100626872	TMEM50A	82861793	82879061
NC_010448.4	158262090	158262416	+	1.73	317	733610	TMEM59	158261936	158290465
NC_010443.5	119946651	119946962	-	3.37	1988	397556	TMOD3	119864878	119948795
NC_010454.4	19023801	19024418	-	2.29	-101	100514791	TMUB2	19019641	19024008

NC_010443.5	159187020	159187425	-	1.05	253	100521706	TNFRSF11A	159128039	159187476
NC_010443.5	159189481	159189879	-	3.46	-2204	100521706	TNFRSF11A	159128039	159187476
NC_010447.5	64316881	64317169	+	2.46	-2324	397020	TNFRSF1A	64319349	64331876
NC_010447.5	64320756	64321044	+	1.88	1551	397020	TNFRSF1A	64319349	64331876
NC_010444.4	1303087	1303572	+	2.46	3966	414906	TNNT3	1299363	1316474
NC_010460.4	19573638	19573869	+	-1.12	1198	100515455	TNPO3	19572555	19673165
NC_010445.4	3993537	3993829	-	3.05	2515	100515017	TNRC18	3901710	3996198
NW_018084874.1	688266	688497	-	0.46	919	110258158	TNRC6A	578944	689301
NC_010447.5	7085890	7086183	+	2.46	-360	110260627	TOB2	7086397	7098643
NC_010456.5	56113852	56114372	+	2.56	1025	100152814	TOMM20	56113087	56127686
NC_010449.5	36977332	36977563	+	-1.71	969	106504412	TOMM6	36976478	36976878
NC_010455.5	158578406	158578673	+	-1.44	1513	100523641	TOMM70	158577026	158629878
NC_010459.5	43711478	43711709	+	-2.71	1323	100518587	TOP1	43710270	43799700
NC_010454.4	60444499	60444787	+	2.78	460	110256129	TOP3A	60444183	60468968
NC_010446.5	73832220	73832451	+	-1.93	-3089	100155888	TOX	73835425	74115352
NC_010448.4	32495985	32496273	+	3.05	3443	100519713	TOX3	32492686	32609923
NC_010448.4	120667284	120667572	-	1.29	2806	100621913	TPGS2	120605480	120670234
NC_010443.5	236406667	236406956	-	3.27	3578	396693	TPM2	236402249	236410390
NC_010445.4	59804474	59804798	+	2.05	-1834	100524432	TRABD2A	59806470	59857941
NC_010444.4	24603589	24603877	+	2.05	984	396629	TRAF6	24602749	24628263
NC_010449.5	46406526	46407089	-	1.78	175	100157796	TRAM2	46323073	46406983
NC_010445.4	38537724	38537955	+	-0.86	1129	100515536	TRAP1	38536710	38595273
NC_010448.4	115785322	115785804	-	1.68	1105	100626409	TRAPPC8	115692509	115786668
NC_010448.4	115786337	115786679	-	2.10	160	100626409	TRAPPC8	115692509	115786668
NC_010450.4	75873181	75873488	-	3.05	2739	100627416	TRIM2	75770344	75876074
NC_010460.4	11151255	11151777	-	3.46	170	100620590	TRIM24	11055211	11151686
NC_010449.5	22768819	22769316	-	1.73	-1405	100144460	TRIM26	22757794	22767662
NC_010444.4	119354432	119354720	-	1.88	-2185	100519890	TRIM36	119304476	119352391
NC_010444.4	57295131	57295569	-	3.20	364	100626621	TRIM41	57285401	57295714
NC_010446.5	43709456	43709882	+	2.46	1830	110260258	TRIQK	43707839	43797108
NC_010450.4	120881534	120881822	+	1.46	2210	100512243	TRMT10A	120879468	120917436
NC_010450.4	120879322	120879843	+	1.01	114	100512243	TRMT10A	120879468	120917436
NC_010443.5	37002648	37002999	-	-0.54	-744	100152782	TRMT11	36930857	37002079
NC_010444.4	7801939	7802337	+	1.61	-46	100520885	TRMT112	7802184	7803556

NC_010446.5	15196996	15197347	-	1.31	-29	100155406	TRMT12	15158866	15197142
NC_010445.4	110383991	110384283	+	2.05	714	100520017	TRMT61B	110383423	110400272
NC_010447.5	3223295	3223583	-	3.05	-1410	100521087	TRMU	3208122	3222029
NC_010453.5	13521331	13521619	-	1.14	-2474	100157635	TRPC4	13300288	13519001
NC_010459.5	38467530	38467864	-	4.27	593	100156677	TRPC4AP	38385650	38468290
NC_010455.5	90639803	90640091	+	2.05	-548	100155608	TSC22D2	90640495	90697671
NC_010455.5	90636389	90636677	+	0.88	-3962	100155608	TSC22D2	90640495	90697671
NC_010455.5	68477657	68478335	+	2.35	-2746	100525362	TSEN2	68480742	68520726
NC_010444.4	115830736	115830992	+	-1.12	-1288	100515191	TSLP	115832152	115837572
NC_010449.5	57186571	57186868	-	1.88	795	100157865	TSPAN3	57162080	57187515
NC_010454.4	34602713	34603001	-	2.05	3766	100514918	TSPOAP1	34578698	34606623
NC_010461.5	47188011	47188242	+	-0.54	1516	100157624	TSR2	47186610	47192465
NC_010458.4	25740929	25741234	-	1.88	700	100625161	TTC33	25695932	25741782
NC_010446.5	103675881	103676261	-	2.14	407	100156166	TTF2	103632377	103676478
NW_018085072.1	148762	149075	-	2.78	-1134	100526252	TTI2	136338	147784
NC_010445.4	44022133	44022421	-	3.46	334	445530	TTL	43980634	44022611
NC_010454.4	6880630	6880918	-	2.46	750	100516219	TTYH2	6838420	6881524
NC_010448.4	97237036	97237326	-	1.68	3511	100623583	TUBB6	97233639	97240692
NC_010451.4	88784475	88784903	-	-1.54	1113	100517907	TWISTNB	88760394	88785802
NC_010451.4	88785026	88785412	-	1.46	583	100517907	TWISTNB	88760394	88785802
NC_010445.4	54935415	54935703	+	0.14	3798	100520954	TXNDC9	54931761	54944153
NC_010446.5	87994247	87994584	-	2.27	-383	100156649	UAP1	87960056	87994032
NC_010448.4	44106621	44106909	+	1.46	3615	100739612	UBA2	44103150	44141396
NC_010452.4	32625276	32625595	-	0.78	598	100739464	UBAP1	32552243	32626034
NC_010457.5	86475170	86475401	+	-0.71	4373	100520938	UBE2E3	86470912	86562297
NC_010460.4	18821146	18821434	+	0.56	632	100626356	UBE2H	18820658	18932909
NC_010449.5	56084963	56085194	+	-0.71	1058	100621770	UBE2Q2	56084020	56144915
NC_010446.5	62051268	62051527	+	-1.86	4829	100152196	UBE2W	62046568	62084679
NC_010448.4	70345953	70346241	+	2.05	284	100737278	UBE4B	70345813	70462434
NC_010458.4	64205487	64205899	-	2.20	-9	100519748	UBLCP1	64182918	64205684
NC_010445.4	37553878	37554166	-	3.05	1233	100512403	UBN1	37512306	37555255
NC_010455.5	19274950	19275285	-	-0.27	1274	100512254	UBP1	19203965	19276392
NC_010455.5	19276656	19276944	-	2.46	-408	100512254	UBP1	19203965	19276392
NC_010449.5	37655453	37655754	+	0.65	-204	100522688	UBR2	37655808	37785301

NC_010457.5	76135461	76135749	+	1.78	149	100521299	UBR3	76135456	76360819
NC_010448.4	77707758	77708046	-	3.05	89	102161705	UBR4	77568409	77707991
NC_010457.5	59265163	59265451	-	1.85	-254	100155117	UGGT1	59141582	59265053
NC_010455.5	135649792	135650238	-	2.85	641	100516340	UMPS	135611857	135650656
NC_010453.5	5359853	5360084	-	-1.35	3385	100515273	URAD	5353524	5363354
NC_010448.4	40043627	40043915	+	1.46	451	100627684	URI1	40043320	40110540
NC_010448.4	157219922	157220210	+	2.05	-455	100620405	USP24	157220521	157385329
NC_010448.4	157219470	157219701	+	-2.93	-935	100620405	USP24	157220521	157385329
NC_010448.4	135525302	135525766	+	1.75	1162	100625972	USP33	135524372	135589742
NC_010447.5	87865447	87865678	+	-0.54	1151	100516911	USP44	87864411	87890913
NC_010448.4	79810133	79810421	-	2.46	-36	100625904	USP48	79744942	79810241
NC_010447.5	63857702	63858318	-	0.88	765	100155342	USP5	63843742	63858775
NC_010443.5	121375673	121375961	-	1.78	337	100157546	USP8	121307690	121376154
NC_010445.4	48872128	48872570	+	2.88	526	100513145	UXS1	48871823	48940722
NC_010454.4	53386590	53386878	-	1.46	-4491	100620549	VAMP2	53378407	53382243
NC_010448.4	98258061	98258292	-	-0.60	-3904	100623614	VAPA	98212335	98254272
NC_010446.5	68072615	68073038	+	0.73	-576	100621816	VCPIP1	68073403	68109221
NC_010446.5	68076847	68077216	+	0.59	3628	100621816	VCPIP1	68073403	68109221
NC_010445.4	17386587	17386875	+	2.46	501	100522924	VKORC1	17386230	17389162
NC_010443.5	230070369	230070812	+	2.11	1251	100153747	VPS13A	230069339	230331343
NC_010456.5	72212337	72212740	+	3.01	1760	100155086	VPS26A	72210778	72241441
NC_010456.5	31865608	31865839	-	-0.12	3890	100737886	VPS29	31860661	31869614
NC_010443.5	129144797	129145060	+	-1.86	590	100513254	VPS39	129144338	129198760
NC_010445.4	77857521	77857752	+	-0.97	1531	100621212	VPS54	77856105	77978194
NC_010445.4	77854828	77855116	+	1.78	-1133	100621212	VPS54	77856105	77978194
NC_010445.4	77856575	77857037	+	2.46	701	100621212	VPS54	77856105	77978194
NC_010445.4	83536260	83536548	-	0.61	617	100511127	VRK2	83435250	83537021
NC_010448.4	55034528	55034816	-	1.05	-3543	100523004	VRK3	54973734	55031129
NC_010445.4	120291928	120292216	-	1.88	-3915	100517707	VSNL1	120177897	120288157
NC_010443.5	22923032	22923320	-	0.20	685	100155445	VTA1	22862628	22923861
NC_010456.5	124574657	124574888	+	-2.12	-2647	100152718	VWA2	124577420	124631760
NC_010445.4	24093057	24093345	-	2.20	932	100524076	VWA3A	24028836	24094133
NC_010456.5	87588919	87589262	-	0.61	-412	100157544	WAPL	87492452	87588678
NC_010449.5	121237017	121237482	-	0.73	827	100515390	WARS	121212981	121238077

NC_010443.5	184378521	184379075	-	1.75	176	100152808	WDHD1	184300291	184378974
NC_010456.5	130619414	130619702	+	2.14	634	100156416	WDR11	130618924	130674536
NW_018084979.1	978516	978804	+	3.78	252	100516626	WDR20	978408	1039635
NC_010446.5	102877670	102877988	-	3.78	735	100154556	WDR3	102841459	102878564
NC_010455.5	138109818	138110106	+	1.05	114	100627452	WDR5B	138109848	138113638
NC_010448.4	45420088	45420469	+	1.46	1312	100144885	WDR62	45418966	45477619
NC_010443.5	117946462	117946873	+	0.31	575	100153251	WDR72	117946092	118124772
NC_010457.5	93980517	93980805	+	0.88	-393	100511538	WDR75	93981054	94026479
NC_010455.5	34411675	34411963	-	2.78	371	100522703	WDR82	34387799	34412190
NC_010455.5	34410001	34410232	-	-0.93	2073	100522703	WDR82	34387799	34412190
NC_010444.4	66312447	66312860	-	2.68	185	100519823	WDR83	66307652	66312839
NC_010444.4	66313072	66313405	+	-1.54	314	414435	WDR83OS	66312924	66328000
NC_010443.5	194438970	194439538	+	2.28	-754	100739573	WDR89	194440008	194475876
NC_010449.5	52140491	52140856	-	-1.44	-340	100153988	WHAMM	52108867	52140333
NC_010448.4	144247301	144247648	+	-0.54	4835	100516916	WLS	144242639	144529659
NC_010444.4	51150861	51151346	+	4.05	-2874	100514950	WNT3A	51153978	51203678
NC_010454.4	52955355	52955734	+	1.27	870	100522033	WRAP53	52954674	52968371
NC_010448.4	17230094	17230325	-	-1.12	-2518	100626361	WWP2	17068783	17227691
NC_010446.5	82433310	82433616	-	1.20	2687	100153155	XCL1	82432845	82436150
NC_010455.5	23878989	23879220	-	-1.71	-2254	100134978	XIRP1	23867476	23876850
NC_010447.5	7580382	7580670	-	2.46	2437	110260639	XPNPEP3	7530993	7582963
NC_010445.4	80256869	80257168	+	0.27	1948	397330	XPO1	80255070	80300260
NC_010444.4	91358413	91358749	+	2.27	146	100514895	XRCC4	91358435	91560528
NC_010451.4	9275686	9276037	-	1.46	1733	100518139	XRRA1	9173666	9277595
NC_010447.5	73425101	73425389	-	1.78	-589	110260764	YAF2	73411029	73424656
NC_010447.5	41561271	41561581	+	0.61	-137	100624597	YARS2	41561563	41571829
NC_010448.4	47234532	47234820	-	2.46	-339	100514844	YIF1B	47224345	47234337
NC_010460.4	50970497	50970785	-	2.46	743	100513964	YKT6	50961451	50971384
NC_010451.4	67680367	67680618	-	-1.35	1228	100520400	YOD1	67672640	67681721
NC_010450.4	66088199	66088488	+	0.14	1804	110262114	YTHDC1	66086539	66122717
NC_010454.4	47643306	47643556	-	-0.71	1896	100620600	YWHAE	47601133	47645327
NC_010455.5	157421568	157421856	+	2.46	-312	100521154	ZBTB11	157422024	157460216
NC_010448.4	101756792	101757080	+	3.46	-131	110261173	ZBTB14	101757067	101764531
NC_010443.5	267438397	267438685	+	-0.12	1286	100628020	ZBTB34	267437255	267465619

NC_010448.4	88922489	88922748	+	-1.29	372	100620574	ZBTB8A	88922246	88962855
NC_010446.5	57817083	57817314	-	-2.03	2692	100154167	ZC2HC1A	57761249	57819891
NC_010460.4	10531009	10531240	+	-0.86	1319	100415778	ZC3HAV1	10529805	10588913
NC_010461.5	97380596	97380884	+	2.20	832	100517990	ZCCHC12	97379908	97384384
NC_010443.5	159019541	159019879	-	0.46	2433	100625613	ZCCHC2	158964816	159022143
NC_010443.5	159018073	159018359	-	-2.35	3927	100625613	ZCCHC2	158964816	159022143
NC_010452.4	28996181	28996705	+	1.61	719	100516638	ZCCHC6	28995724	29061179
NC_010444.4	40312861	40313149	-	0.46	-2550	100736787	ZDHHC13	40251511	40310455
NC_010443.5	207837941	207838238	+	1.98	1404	100738488	ZDHHC21	207836685	207913463
NC_010443.5	225711715	225712008	-	2.73	2589	100048933	ZFAND5	225703196	225714451
NC_010449.5	92498066	92498430	-	1.46	-2384	100624279	ZFP36L1	92490069	92495864
NC_010445.4	97203039	97203393	+	-0.71	2821	100513591	ZFP36L2	97200395	97203094
NC_010443.5	253740284	253740572	-	1.78	2186	100517074	ZFP37	253702590	253742614
NC_010448.4	18335078	18335366	-	2.78	236	100620865	ZFP90	18312931	18335458
NC_010450.4	110090019	110090357	+	2.88	268	102167653	ZGRF1	110089920	110168086
NC_010446.5	16102047	16102376	+	-2.35	1438	100154564	ZHX1	16100773	16126674
NC_010449.5	22095913	22096415	+	1.88	3699	100156123	ZKSCAN4	22092465	22105666
NC_010445.4	6443447	6443735	+	1.46	686	100522312	ZKSCAN5	6442905	6464718
NC_010445.4	6441629	6441947	+	3.05	-1117	100522312	ZKSCAN5	6442905	6464718
NC_010444.4	142410484	142410772	+	2.85	-342	100621095	ZMAT2	142410970	142417012
NC_010448.4	96007688	96007976	+	4.05	-475	106507628	ZMPSTE24	96008307	96057131
NC_010443.5	128896037	128896325	+	2.78	2216	100620097	ZNF106	128893965	128957334
NC_010458.4	27814787	27815075	+	3.46	949	102159255	ZNF131	27813982	27846872
NC_010456.5	61048334	61048738	+	1.73	450	100155460	ZNF248	61048086	61065531
NC_010448.4	45859549	45859853	+	1.68	1627	106510533	ZNF260	45858074	45880194
NC_010454.4	58961246	58961534	+	2.46	-3023	106504195	ZNF286A	58964413	58981107
NC_010445.4	7981161	7981449	-	0.88	1388	100518654	ZNF3	7971917	7982693
NC_010444.4	67312413	67312795	+	0.31	456	100510891	ZNF317	67312148	67336323
NC_010449.5	38386043	38386431	-	0.88	2493	100153069	ZNF318	38356128	38388730
NC_010450.4	86034317	86034605	-	3.78	2277	100511765	ZNF330	86019111	86036738
NC_010449.5	21389320	21389608	+	2.68	344	100155322	ZNF391	21389120	21400090
NC_010443.5	149512643	149512931	-	1.46	4497	100152080	ZNF407	149090502	149517284
NC_010444.4	15834341	15834629	-	2.05	64	100515769	ZNF408	15829489	15834549
NC_010455.5	27782626	27782857	-	0.88	588	100626643	ZNF445	27748437	27783330

NC_010448.4	63051292	63051580	+	1.20	2035	110261336	ZNF446	63049401	63057863
NC_010448.4	46302355	46302643	+	0.88	-1762	102165184	ZNF527	46304261	46328232
NC_010444.4	70605897	70606185	-	0.88	-595	100514703	ZNF558	70578256	70605446
NC_010448.4	46443039	46443327	+	1.88	626	100739461	ZNF570	46442557	46468679
NC_010448.4	59746639	59746870	+	0.46	560	100620473	ZNF581	59746194	59767421
NC_010444.4	128157092	128157382	-	-0.54	-468	100512589	ZNF608	128048247	128156769
NC_010446.5	939734	940070	-	3.78	656	110260414	ZNF623	928786	940558
NC_010454.4	58986128	58986485	+	-0.60	-290	100626993	ZNF624	58986597	59006958
NC_010452.4	38817710	38817998	-	1.78	605	110255680	ZNF658	38797314	38818459
NC_010452.4	38818277	38818565	-	-0.95	38	110255680	ZNF658	38797314	38818459
NC_010445.4	17794685	17795066	+	2.88	3442	100515603	ZNF688	17791433	17795431
NC_010460.4	55934638	55934926	+	1.20	2445	110257534	ZNF775	55932337	55954153
NC_010445.4	6482739	6483027	-	1.78	-1427	100519827	ZNF789	6468709	6481456
NC_010460.4	42651016	42651304	-	2.05	754	100514021	ZNRF2	42550571	42651914
NC_010449.5	22077767	22078073	+	-2.71	-4155	100154531	ZSCAN9	22082075	22089724
NC_010448.4	166134064	166134352	+	2.46	-3919	100514968	ZSWIM5	166138127	166326002
NC_010448.4	159479004	159479235	-	0.14	1497	100739208	ZYG11B	159407921	159480617
NC_010460.4	6870080	6870368	-	2.05	10	100524720	ZYX	6861072	6870234

Table S10. Gene Ontology analyses for genes associated with H3K4me3 modifications.

Term type	GO accession	Description	P Value	Gene ID
Biological process	GO:0010769	regulation of cell morphogenesis involved in differentiation	0.00076207	100381266/780428/100144501/595117/445530
Biological process	GO:0010720	positive regulation of cell development	0.00472879	100145887/100381266/780428/100519689/595117
Biological process	GO:0010770	positive regulation of cell morphogenesis involved in differentiation	0.00538142	100381266/780428/595117
Biological process	GO:0016482	cytosolic transport	0.00538142	733576/595112/100144499
Biological process	GO:0007399	nervous system development	0.00715471	100145887/100381266/397674/780428/100144501/100038004/595120/100519689/397247/595117/396584/396629/445530/100144885
Biological process	GO:0007409	axonogenesis	0.00793462	100144501/100038004/595117/445530
Biological process	GO:0032872	regulation of stress-activated MAPK cascade	0.00793462	100145887/397350/448810/396629
Biological process	GO:0051403	stress-activated MAPK cascade	0.00793462	100145887/397350/448810/396629
Biological process	GO:0070302	regulation of stress-activated protein kinase signaling cascade	0.00793462	100145887/397350/448810/396629
Biological process	GO:0000904	cell morphogenesis involved in differentiation	0.00845775	100381266/780428/100144501/100038004/595117/445530
Cellular component	GO:0044440	endosomal part	0.00448365	733576/595120/100519689/595117/595112/100144499/733610
Cellular component	GO:0005819	spindle	0.00841647	100145887/780428/448810/397656/595117/396584/100144885
Molecular function	GO:0050839	cell adhesion molecule binding	0.00699647	100381266/403163/397247/445534/100125376
